# Supplementary material for: Investigation of the feasibility and acceptability of a school-based intervention for children with traits of ADHD: protocol for an iterative case-series study
Source: BMJ Open. 2023 Feb 14;13(2):e065176. doi: 10.1136/bmjopen-2022-065176 (PMC9930561; doi:10.1136/bmjopen-2022-065176)
Supplement: Supplementary data [file bmjopen-2022-065176supp001.pdf]

08/03/2022 Version 4

Protocol for an iterative case-series study to investigate the feasibility and acceptability of a school-based intervention for children with traits of ADHD: Supplementary Material

Full intervention description

The Tools for Schools *FLEX* toolkit will comprise a digital resource of training and resource packages and behavioural strategies organised within 'modules' that cover different classroom-based problems common to ADHD. See the main article for information on the key goals of the toolkit. Supplementary Figure 1 illustrates the participant structure and roles within a school for delivery of the toolkit.

Supplementary Figure 1. Structure of individuals within schools and participant roles in case series study

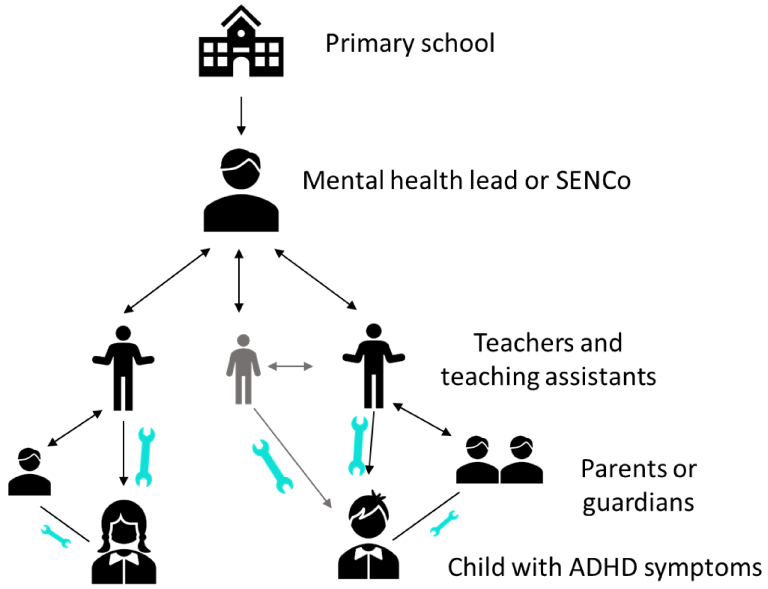

Delivery of the intervention comprises a series of steps, as indicated in the toolkit outline in Supplementary Figure 2, and described below.

Tools for Schools

08/03/2022 Version 4

Supplementary Figure 2. Outline of the *FLEX* Toolkit

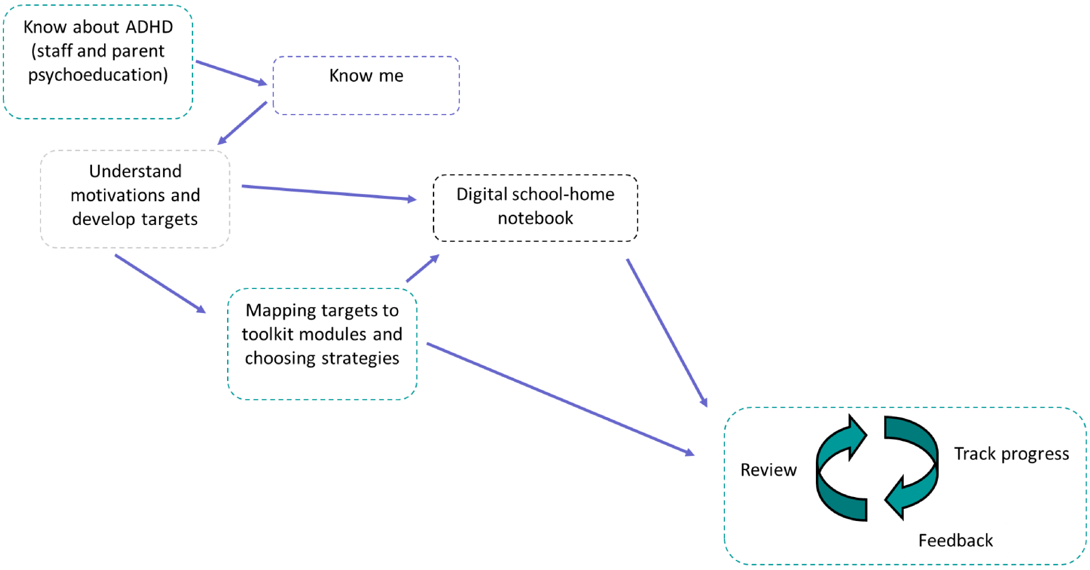

Step 1: Know ADHD

Purpose: Tailored knowledge of ADHD and relevant theory, psychoeducation. Putting staff and parents on equal footing, helping to address power dynamics between school and home and recognise expertise of each party

The toolkit will begin with online psychoeducation training for school staff and parents. This will cover ADHD as well as training on implementing the toolkit itself. Videos from people with ADHD, parents and school staff about their experiences will be included.

Key things the Know ADHD training will cover:

- ADHD is not an issue of morality or simply a deficit in attention
- Involvement of dopamine in the developing brain
- Executive functions and ADHD
- Delay aversion and ADHD
- Other theories about how ADHD traits come about
- Gender differences

- Sensory processing
- The variation between children with ADHD: every child with ADHD is different and unique, and their traits will affect them in different ways
- Growing up with traits of ADHD

Schools will also be asked to plan and deliver activities to promote inclusivity and the concepts of embracing individual differences and diversity at the outset of the study, through a participatory action research approach. Senior leadership and the SENCo will be asked to reflect on their school culture and inclusivity of school policies, make changes to these with the aim of improving flexibility around children with ADHD and their individual needs, and reflect on and evaluate these changes [23]. This might involve participating in national events such as Neurodiversity Celebration Week, having a school assembly or applying for the ADHD Foundation “ADHD friendly schools award” for example. Each school (senior leadership team [SLT] and SENCo) will decide on whether they want to implement any whole-school measures, and what form these will take. They will be supported by the research team but this is not an essential component of the toolkit, rather an exploratory component that will need to be led by each school themselves to be implementable or useful in future versions of the toolkit. The toolkit provides step-by-step instructions for the SLT to conduct an “Inclusion challenge” and makes suggestions as to what systems and processes within the school they may consider changing as part of this.

We will use the Participatory Action Research cycle of Plan, Act, Evaluate, Reflect to structure this. We ask the SLT to consider how their school could be more inclusive for neurodivergent children (including asking for input from children and others in the school) and plan how they could change this. Then they would implement the new ideas (act) for a given period of time; evaluate the changes by seeking staff and student views, and reflect on whether they were successful before implementing further changes. No data will be collected at awareness events or other activities the SLT decide to enact in this step, unless they collect it for their own evaluative purposes. Staff will be

asked to feedback about this during qualitative data collection instances. We anticipate researching the mechanisms of this potential component in further depth in separate research if participants report using this, implementing it and finding this component potentially useful.

Given the aim of this study is to assess feasibility and acceptability of implementation in a real-world context, we have made the decision not to make any component of the flex toolkit mandatory (although we strongly suggest that staff complete the ADHD training and the SLT do the inclusion challenge); the current UK school context is facing severe pressures and resource constraints, and we anticipate that uptake of components will be better and more representative of naturalistic use of the toolkit if they are not mandated. Whether or not to mandate this component in a future trial will be considered based on the findings of this study.

An additional consideration is how to present this information to parents whose child has traits of ADHD but is not diagnosed. The Step 1 learning is titled "know traits of ADHD" and uses minimal language about ADHD as a disorder, talking more about traits of poor attention or concentration, impulsive behaviour or acting without thinking, and hyperactivity. In the learning materials we include a segment on how this relates to the label or diagnosable disorder of ADHD. We are interested to obtain feedback from parents about this.

All of the parents in the study have had contact with their child's SENCo about the child's difficulties or perceived need for additional support in school prior to the study. When selecting and approaching parents, this is discussed expressly with the SENCo and they have a discussion with parents prior to the research team becoming involved. The study information sheet also talks about traits of ADHD, and parents are therefore aware of the nature of the toolkit before they get to Step 1.

Step 2: Know me (the child)

Purpose: Children with ADHD are more able to engage with something if it interests them. They often feel misunderstood by others and this activity is intended to allow staff to understand and empathise with the child, seeing things from their perspective. Children may not be able to verbalise their emotions and feelings and understanding how they behave when happy, sad, angry or excited is an important component of functional behaviour analysis and behaviour change approaches. This activity aims to draw these out for each individual.

Children will complete a strengths-focussed activity with the teacher or TA to identify strengths or hobbies that could be used to personalise the intervention and to help school staff understand the child from their perspective. This activity will be bespoke depending on what the child wants and likes to do and could be delivered to a whole class, but might include making a film or animation, creating a story or photo board, or building a virtual version of oneself on a computer game.

Step 3: Understand motivations and develop targets

Purpose of step: Sharing knowledge and expertise between school and home, focussing on understanding antecedents and consequences of behaviour in order to understand how best to modify. Mutually deciding on which targets are most important (and the relationships between potential targets)

At the end of the baseline term and the beginning of the intervention term, SENCos, teachers and other relevant staff will work alongside the child and family to complete a functional behaviour analysis of two problem areas where the child is currently struggling. We have chosen to constrain this to two areas in order to provide focus during the use of the toolkit in one school term for research purposes, and to support those testing the toolkit to identify the key areas for improvement and therefore observable change. Understanding the antecedents and consequences of behaviour will be crucial to identify where to intervene and to understand which modules and strategies from the toolkit are likely to be useful. The meetings will result in identifying two core

behaviours that will be transformed into the intervention "toolkit targets". The core problems will be operationalised into tangible and achievable goals (e.g. stay in seat for 5 minutes, with the number of minutes increasing as the child meets the target; having more than one friend to play with at lunchtime, starting with one day per week and increasing over time; having three adults at school they can go to if they have a problem, starting with one adult and knowing what and how to communicate to them if there is a problem) over the course of two meetings.

The form of these meetings may vary depending on parent engagement and preferences. One option that will be piloted is inviting families and children to join these meetings via videoconference. This would allow the child to feel involved in the process if they wish to engage, but also provides them with the option to withdraw from the conversation, and parents will make the decision as to whether they wish for their child to be involved in these meetings. Through discussions with the planning group, parents identified that they wish to be consulted and to bring their perspective to these meetings, however they may also find such meetings are difficult emotionally, as the focus will be on their child's difficulties. As such, parents may feel more empowered or comfortable joining from their home environment. Alternately, parents and children may wish to attend these meetings in person at school. Parents will make the decision as to where/through what medium the meeting takes place.

The purpose of this step is to encourage school staff and parents to become aware of the layers of context around the child, highlighting potential points for intervention that do not rely on intrinsic changes to the child themselves. This step of the intervention focusses on what achievements it is hoped the child will make, however the strategies that will be implemented to achieve these are primarily designed to adapt the environment around the child, for example scaffolding them with classroom tools and activities to improve organisation through making it clearer to the child where their possessions can be found or visual aids to demonstrate what will be needed for the next activity; introduction of whole-class games on identifying and providing positive feedback to peers

where all classmates are reinforced for "picking up the positives" to improve self-esteem; or incorporation of movement-based learning strategies throughout the school day to improve ability to concentrate, and the teacher changing the format of the work that the child is asked to complete.

Step 4: Digital school-home notebook

Purpose: Establishing and maintaining positive communication between teachers, TAs and parents. Research shows that conflictual and negative relationships between school and home are common for children with ADHD. Breaking negative communication cycles and establishing positive communication will improve sharing of information and ideas between home and school, and better support the child across contexts.

A communication platform for regular contact between staff and parents will be established once the child's toolkit targets have been agreed. This was initially intended to take the form of a digital daily report card (dDRC) with staff updating home daily on the child's progress with their targets, and parents reinforcing this at home. The purpose of this component is to develop habits of positive communication about progress and child strengths, rather than to function as a "behaviour log" or to report incidents during school.

The frequency and nature of communication using the notebook will be flexible to suit the needs of the individual child and participants surrounding them; the DRC format will be proposed as the suggested model as this has the most existing evidence for effectiveness [24 25]. However, other forms of successful communication may already be in place between school and home, or daily communication may be considered burdensome and not beneficial to both parents and teachers for a wide variety of reasons. As such, staff and parents will be asked to mutually agree on frequency of communication using the digital school-home notebook, and this will be reviewed and modified as required during the intervention period.

Step 5. Mapping targets to toolkit modules and choosing strategies

Purpose: identifying strategies within modules that are most likely to be effective based on functional behaviour analysis and understanding of the child

The SENCo and teacher will link the child's targets to the most pertinent modules and outcomes in the toolkit using a graphical map (currently called the behaviour web). This may be initially discussed in the two meetings to understand behaviour and develop targets, or may be decided following these meetings. The functional behaviour analysis will be drawn upon with a focus on the current consequences relating to the target behaviour, and alternate consequences that could encourage more adaptive behaviour.

The toolkit modules are based on individual ADHD-related outcomes selected by consensus in a Delphi study. They are:

1. Organising, planning and reminding (describe to differentiate from organising)->means self-monitoring and self-regulating
2. Paying attention
3. Feeling good (self-esteem, self-worth)
4. Belonging and relating (relationships)
5. Pausing for thought (inhibiting impulsive behaviour)
6. Channelling energy (hyperactivity)

Modules will contain a bank of approximately 10 non-pharmacological strategies that can be used at individual or classroom level to target the outcome, organised into domains such as "Behaviour management" "Training tools" and "Changing the classroom" through the co-creation process. The planning group have indicated that the focus of the toolkit should be on inclusivity: supporting children to remain in the classroom and allowing the child to access the same education and

opportunities as their classmates. Strategies will be developed with this goal in mind. In addition, modules will contain resources on maintaining strategies once implemented.

Step 6. Implementing and reflecting on strategies

Purpose: produce improvement in target behaviours that can be maintained

Once teachers and SENCos have determined the modules to implement through consideration of the functional behaviour analysis and the child's agreed targets, the SENCo will support the teacher and TAs to select and implement strategies. Strategies have been designed to be primarily group-based games or activities, or reminders and support for teachers to incorporate new techniques to adapt around the child, with the aim that this will lead to improved outcomes for the child. None of the strategies require the child to be removed from the classroom, and although some could be used 1:1 with the child, they are primarily presented as group-level activities to promote inclusion. A significant number of strategies focus on "belonging and relating", specifically aiming to promote peer inclusion, celebrate individual differences, and destigmatise neurodevelopmental (and other) differences.

We anticipate that one module will be implemented in each half-term of the intervention term, however there will not be a restriction on this, nor how many strategies within a module can be attempted; we anticipate that some strategies will have already been tried by a school or teacher, or it may be apparent that other strategies do not suit the child and so different strategies may be selected. Repeated measures of ADHD symptoms, classroom functioning, and measures specific to the outcomes relevant for each module will be collected from teachers, TAs and children as appropriate (Table 3 shows the data collection schedule relative to the stage within the study).

Co-production of the toolkit with the planning group

Parents, children with ADHD, adults with ADHD, SENCos, educational psychologists and teachers (the planning group) were initially informally interviewed about their experiences, and what they

perceive works for them. These findings were narratively synthesised and written up in one document.

They contributed to an exhaustive list of the kinds of behaviours that may form targets for the intervention, and the modules that these would fall within (e.g. being 'easily led by others' mapping to the 'pausing for thought' module); with some behaviours mapping to multiple modules depending on the underlying reason (e.g. 'risky or dangerous behaviour' mapping to either 'paying attention and engaging' or 'pausing for thought' depending on whether the primary reason is impulsivity or inattentiveness).

The intervention logic model of change was reviewed and refined with the planning group, who then had input into the performance objectives (the smallest steps of behaviour change required to meet each behavioural goal for each person e.g. teacher, child, senior leadership) through reviewing the lists in an 'activity workbook' format, adding thoughts and attending discussion meetings.

These performance objectives were also categorised by the determinants of behaviour (whether the change in behaviour required aligned with skills, values, beliefs, attitudes, knowledge or experience). Initial strategy ideas were then drafted by AR to match performance objectives with input from behaviour change taxonomies to select relevant delivery methods.

These draft strategy ideas were then reviewed by the planning group, and changed entirely or improved based on their input. Strategy instructions were then written by AR, reviewed by the planning group once more and refined and the prototype toolkit was produced. PDF resources to support teachers in delivering each strategy were generated by the research team, with the planning group suggesting resource ideas and providing further input and feedback at the 'alpha testing' stage of the prototype. Resources and strategies were then refined and finalised, prior to the beta version (to be used in the feasibility study) being rolled out.

Example questions for Toolkit iteration feedback interviews

1. *Could you tell me about how you have used the toolkit?*
2. *What have your experiences been of using the toolkit? Let's talk about it step-by-step so you can give me your reflections on each component. First, overall did you think the toolkit was useable in Primary school? Why/why not? And do you think other schools and families would find it acceptable? Why/why not? Do you think other schools and families would be able to implement it? Why/why not?*
3. *What did you like most about the toolkit? What did you like least? Could you tell me some more about that?*
4. *How was the Know ADHD component? Did you use it, what was good about it and what was difficult or challenging? What would help you to use it better in the future? Was there anything missing that you think is important for people to know about ADHD?*
5. *How was the Know Me component? What did you do with the child to complete this activity? What format did the child choose (e.g. video, storybook)? How did you feel about it, and how do you think the child felt about doing the activity? Did you find the product of this Know Me step was useful for the rest of the toolkit? Could you tell me why you think that? Would you make any changes to the Know Me step or instructions, and what would they be?*
6. *Who set up the meetings with school staff to assess and understand the two main problems? How did those meetings go and who was there? When did you have the meetings? What was good and bad about the meetings? Did you manage to define two target goals for the child? Were parents and the child involved (if so how)? Would you do anything differently if you did it again? Should the toolkit have different instructions? How did you understand the antecedents, behaviours and consequences of each behaviour? Who helped to figure those out, how and how long did it take? Was that too much time for you?*

Table of quantitative measures

Outcome	Measure
ADHD symptoms	Strengths and Weaknesses of ADHD and Normal Behavior Scale (SWAN)
Classroom functioning	Problem behaviour subscale of the Social Skills Improvement System (SISS)
Dimensional psychopathology	Strengths and Difficulties Questionniare
School functioning	Academic and social skills subscales of the SISS
Child-reported satisfaction with school	How I Feel About My School questionnaire
Teacher wellbeing	Warwick-Edinburgh mental wellbeing scale 14-item Teacher survey; the Relationship with Work survey from the Maslach Burnout Inventory-General Survey; the Teacher Sense of Efficacy Scale;
Health and education resource use	Bespoke questionnaire available from the authors on request
Child quality of life	Child and parent report Child Health Utility-9D
Additional module measures (where not captured by the above)	
Child self-esteem	Harter Self-Perception Profile for Children
Belonging and relating	PROMIS Pediatric Peer Relationships - Short Form 8
Organising, planning and reminding	Children's Organizational Skills Scale

Risks and mitigation plans

Risk 1. Schools are open but due to periods of Covid-related closure, child development and socialisation has been impacted.

Mitigation: as part of the toolkit development process we are liaising closely with primary schools, parents and teachers to monitor ongoing changes to school environments and cultures. We anticipate that, if anything, there will be greater demand for the toolkit if more pupils are struggling with relevant interpersonal and developmental challenges. There may be the need to adapt the focus of modules, for example, to support school attendance explicitly. Potential adaptations will be discussed with the academic advisory team and planning group before implementation.

Risk 2. Schools are closed due to ongoing Covid-19

Mitigation. The planning group has advised that many of the children meeting eligibility criteria for the study have attended school through prior lockdowns and closure periods due to their vulnerable status and additional needs. We will explore whether sufficient children and staff are attending school at the time of recruitment and data collection, and if so will continue as planned with implementation of the intervention. Data collection will be completed remotely through digital questionnaires and online video conferencing software meeting data protection requirements if external (i.e. research) staff are unable to access schools due to covid-19 lockdowns.

Risk 3. Unanticipated adverse effects of intervention

Mitigation: adverse effects will be monitored through qualitative data collection during the use of the intervention, and participating school staff will follow a defined procedure for reporting of negative impacts as soon as possible after they come to light. Should a child or other participant of the study report severe worsening of ADHD symptoms or distress, the PI will consult with clinically-trained members of the academic advisory board to decide on the most appropriate course of action. This may include: the clinical academic speaking directly to staff and families to ascertain whether the adverse effects are caused by the intervention or research study or are external to this, the child and family withdrawing from the study, or bespoke plans being put in place to monitor and manage the situation. Should a child or family withdraw from the study due to this, their existing data will not be used unless express consent is given at the time of withdrawal, and they will be able to continue to access the prototype intervention (as long as the study team are confident that this is not the cause of adverse effects) if they wish.

Limitations

This study is a feasibility study, and due to the iterative case-series design not all participants will get the same version of the study prototype. Therefore we will not be able to quantitatively measure or compare changes in our outcome measures across all study participants, instead we are restricted to examining whether individual trends of change are replicated across individuals.

In addition, due to the modular nature of the toolkit, the sample size for a future cluster randomised controlled trial may have to be large in order to obtain sufficient numbers of participants using each module.

08/03/2022 Version 4

Model consent form

Tools for Schools: a prototype toolkit for ADHD interventions to be used in primary school teaching.
Consent form for parents or guardians on behalf of children –Version 3: January 2022

I have read the provided information sheet, Version 4, concerning Tools for Schools project and I understand what it is about.
I fully understand what is expected of me and all my questions have been answered to my satisfaction.
I understand I am free to request further information at any stage.

As a **parent or guardian on behalf of my child**, I am aware that:

(please circle Yes or No)

1. My child's participation is entirely voluntary. Yes / No
2. I am able to withdraw my child's participation from the project without consequence or disadvantage. Yes / No
3. I understand that relevant sections of the data collected during the study may be looked at by members of the research team, and individuals from the University of Exeter where it is relevant to my taking part in this research. I give my permission for these individuals to have access to my records. Yes / No
4. The researchers do not anticipate any of the participants to experience any discomfort or harm during the project. Yes / No
5. I can contact the researchers with any questions, queries or worries at any point during the study (using the contact details provided in the information sheet). Yes / No
6. My child's data will be anonymous in any published works relating to the project. Yes / No
7. In the future, my child's anonymous data may be used in other related research. Yes / No
8. Recordings can be taken of interviews and class groups my child takes part in. Yes / No

08/03/2022 Version 4

9. My child will take part in lessons that may ask them to do things differently from other classmates. Wherever possible, this will be done in the most subtle way so as not to draw attention to my child, but I understand this may not always be possible.
Yes / No

10. My child will be asked to complete questions that other children will not do. Yes / No

11. My child might be asked to leave the classroom to do different learning/lessons, for example daily report cards. Yes / No

12. My child might take part in an 'active interview' (such as playing together or doing an activity) at home or another suitable location. I may be asked to join.
Yes / No

13. Records (e.g. photographs) may be taken of my child's work. Yes / No

14. In the future, researchers may contact me again to follow up on my child's progress.
Yes / No

By signing below, I agree/consent to take part in this research project.

………………………………………..	………………………………………..	………………
(Printed name of child)	(signature of child)	(Date)
………………………………………..	………………………………………..	………………
(Printed name of parent/guardian)	(signature of parent/guardian)	(Date)
………………………………………..	………………………………………..	………………
(Printed name of researcher)	(signature of researcher)	(Date)

Tools for Schools

08/03/2022 Version 4

Model information sheet

Tools for Schools: Development and early testing of a school-based toolkit to support children in school

Information Sheet for Parents, Guardians or Carers: Version 4 Jan 2022

18

08/03/2022 Version 4

Contents

Glossary 2
Background and aims of this research study 3
Overview of the Tools for School toolkit 4
Your role in the research 5
What does your child need to know? 6
Potential Unintended Consequences 7
Withdrawing your consent 8
Confidentiality and data protection 8
Funding 9
Ethical approval 9
Contact details 10
Complaints 10

UNIVERSITY OF EXETER | MEDICAL SCHOOL

FUNDED BY NIHR | National Institute for Health Research

http://blogs.exeter.ac.uk/toolsforschools/

Glossary

ADHD = Attention Deficit/Hyperactivity Disorder

SEN(D)Co = Special Educational Needs (and Disabilities) Coordinator

TA = Teaching Assistant (also known as Learning Support Assistant, LSA)

Tools for Schools

Thank you for showing an interest in this research. It focuses on early testing of a school-based toolkit of behavioural strategies and adjustments. These will improve the school experience of students who are hyperactive, impulsive or have problems with attention, causing difficulties for the child at school.

You have received this information sheet after your child's school have approached you because they think your child might benefit from taking part in this research. Please take time to carefully read this leaflet so that you have the information you need or listen to the audio/video version here. You can also request a meeting with the research team or your school's SENCo to discuss what will happen in the study and if you would like further clarification on the overview of the toolkit and study protocols.

Do ask the research team any questions you have before signing the consent form for you or your child. It is important that you are comfortable for both you and your child to take part in this study. Any other parents, guardians or main carers of the child who might like to take part would also be welcome.

If you decide to participate, we thank you. If you choose not to, there is no disadvantage to you, and we thank you for considering our project.

Background and aims of this research study

Every classroom has at least one child (on average) who will struggle to sit still, pay attention and resist impulses to do things like jumping out of their seat. Strategies are needed to help teachers support these students, but current advice is often complicated, and no child is the same. This can lead to teachers struggling to develop the right strategies for each child.

In this project, we have worked closely alongside experts, teachers and parents to develop a prototype (early version) of a toolkit which includes a range of strategies for teachers to use to support school children. Your child's school has agreed to take part in this research already and think your child would suit this toolkit and research study, which is why we are contacting you. The toolkit will be used with your child for one school term, but we will be collecting information from you, your child and their teachers for one term before this and for one school term after the toolkit is tried.

Tools for Schools

The aim of this current step of the study is to see if the toolkit is acceptable and practical to school staff, children and their parents. It is important to know that we are not trying to diagnose or "treat" your child. The toolkit is at an early stage, and we are not yet sure how much it will help. But we hope that in the future this toolkit will help children to have a better primary school experience.

Overview of the Tools for School toolkit

The Tools for Schools toolkit will be mainly digital (a website), with several key steps as shown below. We want the toolkit to support your child to remain in the classroom and allow them to access the same education and opportunities as their classmates. Your child's SENCo, class teacher and teaching assistants will use the toolkit with your child in school. To keep you as parents 'in the loop' and to make the most of your knowledge of your child, you will be asked to join in for several parts of the toolkit. You can find out more information about the parts of the toolkit through the videos and descriptions on our website.

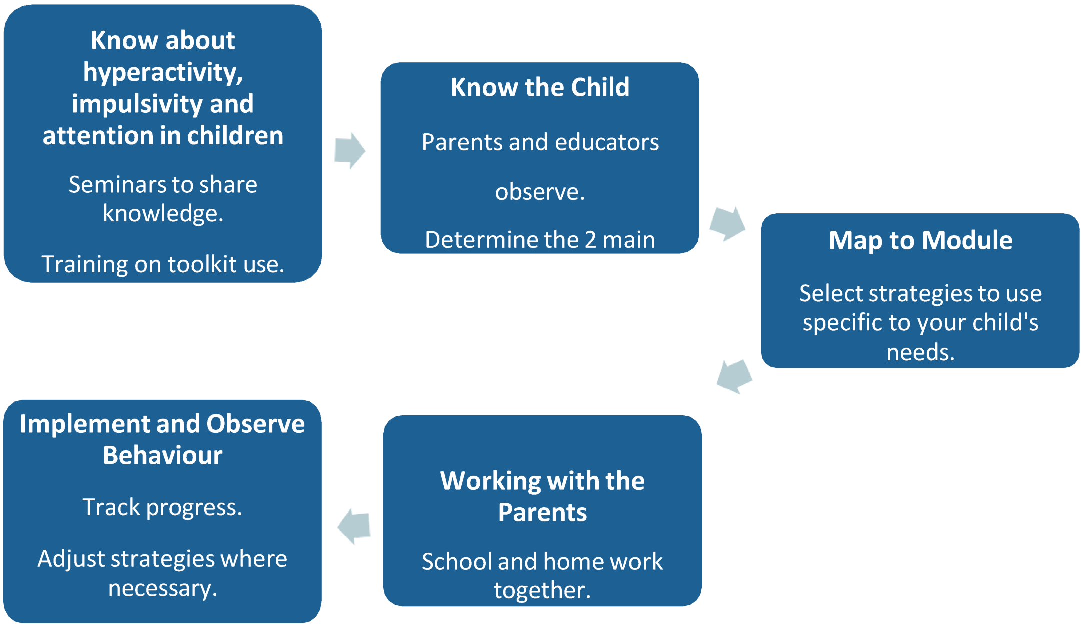

If you would like to find out more about any of these stages/the toolkit, please visit: http://blogs.exeter.ac.uk/toolsforschools/

Your role in the research

As a parent, guardian or carer of a child using the Tools for Schools intervention, your role will include:

- Possibly completing a brief questionnaire to see if your child is eligible to take part.
- Watching an introductory video and completing training provided by the Tools for Schools research team.
- Completing questionnaires about the healthcare, education and social services you use to support your child, and your child's wellbeing and quality of life (both before and after the toolkit is used).
- Two or three interview conversations with research staff. It is up to you where these take place (e.g. over the phone/zoom, at home or another convenient location for you). These may be recorded
- Taking part in two meetings with your child's teacher and SENDCo to help identify what the toolkit should target to best support your child (you could join these online or in person).
- Working with your child and school staff to use strategies selected in the toolkit.
- Chatting to the research team over the phone about any good and bad things you are experiencing using the toolkit so we can make changes if need be. These may be recorded.
- Using a digital home-school notebook – this will allow you and your child's teacher to share information on your child's progress and ensure strategies are reinforced at home.
- Give the research team permission to collect information about your child from you, school staff and your child.

To show our gratitude, we will provide £20 to your family (£10 for you and £10 for your child) for participating in the interviews.

Tools for Schools

We would also like you to give the following permissions on behalf of your child:

- Permission for your child to participate in the research and try the strategies in the toolkit with school staff support.
- Permission for one of the research team to observe your child in school to see how their behaviour changes with different strategies (this will be done subtly without making your child stand out from their classmates and will not disrupt your child's school day).
- Permission for us to ask your child to complete two short age-appropriate questionnaires; one about how they feel about their school, and one about their quality of life (twice during the research study).
- Permission to keep records/photographs of your child's work
- Permission for the research team to conduct an 'active interview' (such as playing together or doing an activity) at home or another suitable location with your child, finding out more about whether they liked or disliked the toolkit strategies. You, another parent/guardian or a sibling can join the child for this if they want you to.

What does your child need to know?

Your child should understand that they are participating in research but it is up to you how much you want to tell them. The toolkit is being tried as part of the usual school day, with SENCos and teachers working to support children in school as usual, so your child need not know that they are doing something new or different if you think this will make them uncomfortable.

We will need your consent for your child to participate, and their verbal agreement that they are happy to take part in a research study and to complete questionnaires. We want to ensure we are mindful of your child's "emotional thermometer" and will not be making your child do something they do not want to do. To achieve this, we will discuss with you the best way to get permission from your child to take part in certain strategies in a way that you and your child are happy with.

Potential Benefits

Benefits for your child include improving their relationship with their teacher and making school a more positive experience for them through:

- School staff and parents becoming better informed about how to work with children who struggle with attention, impulsiveness, or hyperactivity and what it is like for families and children living with children who have issues with this (sometimes diagnosed as ADHD).
- Improvement in teachers' awareness about attention, impulsiveness, or hyperactivity in the classroom.
- Giving teachers easy-to-use strategies to better identify and support students with their attention, impulsiveness, or hyperactivity

Benefits for you include:

- Picking up some tips from the strategies that may help you and your family at home
- Improve your communication with your child's school through the home-school notebook

We hope that access to the Tools for Schools toolkit will help your child's school experience and relationships with school staff. However, the main aim of this study is to test a prototype before it is tried with a wider range of schools and children. In the long run, we hope this will improve support (mostly at school, but also at home) for many children in education.

Potential Unintended Consequences

It is possible that the toolkit may lead to children feeling singled-out from their peers due to being treated differently. We aim to avoid this and any other negative impacts wherever possible so please talk to us about any concerns you may have. Via the toolkit children in the study will hopefully develop social skills and greater self-esteem which may help them build stronger relationships with other children. However, you and your child are free to withdraw at any time if you or they wish to.

Possible negative impacts of the toolkit will be monitored throughout the study and school staff will follow a defined procedure for reporting any negative impacts as soon as possible after they come to light. The research team will abide by any existing safeguarding protocols your child's school has as well.

Should a child or other participant of the study report severe worsening of symptoms or distress, you will be informed and the use of the toolkit with your child will be temporarily stopped. From there the lead researcher (Dr Abby Russell) will consult with members on the study advisory board to decide on the most appropriate course of action. This can include: a clinician speaking directly to you and the school to see whether the negative effects are caused by the toolkit or research study or are external to this; your child and family withdrawing from the study; or individual plans being put in place to monitor and manage the situation.

Withdrawing your consent

- You (and anyone else involved) may withdraw from participation in the study without needing to provide a reason at any point.
- You can choose to withdraw you or your child's participation and/or any data you and they have provided.
- Data can only be removed so long as the withdrawal is done before the data is anonymised, as at this point, we will not know which information is linked to your individual child.
- Should you withdraw from the study due to negative effects, your existing data will not be used unless you give us consent at the time that you withdraw.
- Your child will be able to continue to access the prototype intervention (as long as the study team are confident that this is not the cause of adverse effects) if they wish.

Confidentiality and data protection

The University of Exeter processes personal data for the purposes of carrying out research in the public interest. The University will endeavour to be transparent about its processing of your personal data and this information sheet should provide a clear explanation of this. If you do have any queries about the University's processing of your personal data that cannot be resolved by the research team, further information may be obtained from the University's Data Protection Officer by emailing informationgovernance@exeter.ac.uk. or at http://www.exeter.ac.uk/ig/

The data we will collect from you includes recordings of your interviews with the research team, notes taken by the interviewer at the time and any questionnaires that we ask you to complete. The data we will collect from your child includes a recording of their active interview, any questionnaires they complete and information from observing them at school.

Recordings will be written down word for word and anonymised before being analysed along with other interviews we are conducting. The data collected will be securely stored in such a way that only the researchers working on the project can access it. In the interview if you feel uncomfortable, you will be reminded of your right to not answer any particular question(s).

The results of this project may be published, but any data included will not be individually identifiable – so nobody will know which schools or children took part. A summary of the study findings will be sent to you. You will be provided with a copy of your interviews as well if you wish. Additionally, the anonymised data you provide may be used other related research – however this is optional.

Funding

This research is funded by the National Institute of Health Research (NIHR): the research arm of the NHS. Specifically, it is funded by an Advanced Fellowship – Stage 2 <details>. The funding currently covers the project for 5 years, which began in July 2020.

Ethical approval

This study has received ethical approval from the University of Exeter College of Medicine and Health Research Ethics Committee (reference Jan22/B/300).

08/03/2022 Version 4

Contact details

If you have any questions about the Tools for Schools project, either now or in the future, please feel free visit our website for further information, or to contact [removed for review] directly, who leads the research team:

[Researcher name}

Children and Young people's Mental health research collaboration (ChYMe)
University of Exeter College of Medicine and Health

[phone numbers]

ToolsForSchools@exeter.ac.uk

[researcher email and twitter

handle]

Complaints

If you have any complaints about the way in which this study has been carried out, please contact the Chair of the University of Exeter Medical School Research Ethics Committee:

cmhethics@exeter.ac.uk

Russell AE, *et al. BMJ Open* 2023; 13:e065176. doi: 10.1136/bmjopen-2022-065176
