# Supplementary material for: Investigation of the feasibility and acceptability of a school-based intervention for children with traits of ADHD: protocol for an iterative case-series study
Source: BMJ Open. 2023 Feb 14;13(2):e065176. doi: 10.1136/bmjopen-2022-065176 (PMC9930561; doi:10.1136/bmjopen-2022-065176)
Supplement: Author's manuscript [file bmjopen-2022-065176.draft_revisions.pdf]

BMJ Open is committed to open peer review. As part of this commitment we make the peer review history of every article we publish publicly available.

When an article is published we post the peer reviewers' comments and the authors' responses online. We also post the versions of the paper that were used during peer review. These are the versions that the peer review comments apply to.

The versions of the paper that follow are the versions that were submitted during the peer review process. They are not the versions of record or the final published versions. They should not be cited or distributed as the published version of this manuscript.

BMJ Open is an open access journal and the full, final, typeset and author-corrected version of record of the manuscript is available on our site with no access controls, subscription charges or pay-per-view fees (http://bmjopen.bmj.com).

If you have any questions on BMJ Open's open peer review process please email info.bmjopen@bmj.com

BMJ Open

Protocol for an iterative case-series study to investigate the feasibility and acceptability of a school-based intervention for children with traits of ADHD

Journal:	*BMJ Open*
Manuscript ID	bmjopen-2022-065176
Article Type:	Protocol
Date Submitted by the Author:	27-May-2022
Complete List of Authors:	Russell, Abigail; University of Exeter, Children and Young People's Mental Health Research Collaboration (ChYMe), College of Medicine and Health<br>Dunn, Barney ; University of Exeter, Department of Psychology<br>Hayes, Rachel; University of Exeter, Children and Young People's Mental Health Research Collaboration (ChYMe), College of Medicine and Health<br>Moore, Darren; University of Exeter, Graduate School of Education<br>Kidger, Judi; University of Bristol, Population Health Sciences, Bristol Medical School<br>Sonuga-Barke, Edmund; King's College London, Institute of Psychiatry, Psychology and Neuroscience<br>Pfiffner, Linda; University of California San Francisco, Department of Psychiatry<br>Ford, Tamsin; University of Cambridge, Department of Psychiatry
Keywords:	Developmental neurology & neurodisability < PAEDIATRICS, Community child health < PAEDIATRICS, Child & adolescent psychiatry < PSYCHIATRY

SCHOLARONE™
Manuscripts

Protocol for an iterative case-series study to investigate the feasibility and acceptability of a school-based intervention for children with traits of ADHD

For peer review only

Abigail Emma Russell[1], Barney Dunn[2], Rachel Hayes[1], Darren Moore[3], Judi Kidger[4], Edmund Sonuga-Barke[5], Linda Pfiffner[6], and Tamsin Ford[7]

[1]Children and Young People's Mental Health Research Collaboration (ChYMe), College of Medicine and Health, University of Exeter, Exeter, UK

[2]Department of Psychology, University of Exeter, Exeter, UK

[3]Graduate School of Education, University of Exeter, Exeter, UK

[4]Population Health Sciences, Bristol Medical School, University of Bristol, Bristol, UK

[5]Institute of Psychiatry, Psychology and Neuroscience, King's College London, London, UK

[6]Department of Psychiatry, University of California San Francisco, San Francisco, USA

[7]Department of Psychiatry, University of Cambridge, Cambridge, UK

Corresponding author: Abigail E Russell, University of Exeter, College of Medicine and Health, South Cloisters, St Luke's Campus, Exeter, UK EX1 2LU. E-mail: a.e.russell@exeter.ac.uk

Word count: 6023

For peer review only - http://bmjopen.bmj.com/site/about/guidelines.xhtml

Abstract

Introduction: Attention deficit/hyperactivity disorder (ADHD) is a prevalent and impairing cluster of traits affecting 2-5% of children. These children are at risk of negative health, social and educational outcomes and often experience severe difficulties at school: effective psychosocial interventions are needed. There is mixed evidence for existing school-based interventions for ADHD, which are complex and resource-intensive, contradicting teachers' preferences for short, flexible strategies that suit a range of ADHD-related classroom-based problems. They are also poorly evaluated. In this study, a prototype intervention comprising a digital 'toolkit' of behavioural strategies will be tested and refined. We aim to refine the prototype so that its use is feasible and acceptable within school settings, and to establish whether a future definitive, appropriately powered, trial of efficacy is feasible. This novel iterative study aims to pre-emptively address implementation and evaluation challenges that have hampered previous randomised controlled trials of non-pharmacological interventions. **Methods and analysis**: A randomised iterative mixed methods case-series design will be used. Schools will be randomised to the time (school term) they implement the toolkit. Eight Primary schools and 16-32 children with impairing traits of ADHD will participate, along with school staff and parents. The toolkit will be refined after each term, or more frequently if needed. Small, theory-based and data driven changes hypothesised as relevant across school contexts will be made, as well as reactive changes addressing implementation barriers. Feasibility and acceptability will be assessed through quantitative and qualitative data collection and analyses in relation to study continuation criteria, and ADHD symptoms and classroom functioning will be tracked and visually evaluated to assess whether there are early indications of toolkit utility. **Ethics and dissemination**: Ethical approval has been obtained. Results will be presented in journal articles, conferences and through varied forms of media to reach policymakers, stakeholders and the public.

Keywords: ADHD; school-based intervention; behavioural strategies; case-series; feasibility; acceptability

For peer review only - http://bmjopen.bmj.com/site/about/guidelines.xhtml

Article Summary

Strengths and limitations of this study

- This study uses an iterative staggered design in order to refine an intervention throughout the research process, maximising chances of it being acceptable and feasible to further evaluate.
- Measures of acceptability and feasibility of the intervention and of the ability to evaluate it in a rigorous trial setting will be reviewed against continuation criteria throughout the study.
- Data capture will be nuanced and detailed: multiple informants will provide relevant data, including school staff and parents. Data on both positive and adverse outcomes will be captured.
- However, the iterative nature of the study means that different participants will not receive the same intervention, therefore group-level effects cannot be delineated. Alternate analyses will be utilised to ascertain whether there are patterns of change in symptoms and functioning that replicate across individuals instead.
- The modular nature of the toolkit may mean that the sample size required for a future powered trial is very large; this study will allow calculation and specification of this and consider potential trial designs to overcome such a limitation.

Introduction

Children and young people's mental health and neurodevelopment (including ADHD) is a government priority. In the 2017 green paper, the pivotal role of schools in supporting good mental health was acknowledged. By the end of 2023, >20% of schools across the UK will have a designated mental health lead with the aim of improving access to evidence-based treatments.[1] Cost-effective, implementable, sustainable evidence-based mental health interventions that can be delivered in schools are therefore urgently needed.

For peer review only

Attention deficit/hyperactivity disorder (ADHD) is a neurodevelopmental disorder affecting 2-5% of children, characterised by impairing levels of impulsivity, hyperactivity and/or inattention.[2] Multiple negative life course outcomes are associated with childhood ADHD including high rates of co-occurring mental disorders, accidents, poor educational and occupational attainment and antisocial behaviour.[3-5] Other neuropsychiatric disorders and unintentional injury are strongly associated with ADHD, which are the leading cause of years lost due to disability worldwide in 10-24 year olds.[6] Costs to the health, education and judicial systems, social services and economic loss are substantial; 24% of the mean annual cost of £5,493 is attributable to National Health Service (NHS) resources.[7] Pharmacological treatment is available and effective for some, but not appropriate, acceptable or tolerable for all children. Response is often partial, with tolerance taking over 2-3 years to develop. Even with drug treatment, ADHD causes particular problems in school, resulting in the outcomes described above.[8] Licensed ADHD medications are controlled drugs and in order to access these families must receive a clinical diagnosis of ADHD, waiting lists for which in the UK are currently backlogged. Medication is not appropriate or preferable for all children with ADHD or as a first-line treatment for children with traits that are not severe; and non-pharmacological intervention options that can be used as a precursor, alternative or supplement to pharmacological treatments are important.

ADHD causes problems in the classroom for the child, the teacher and for other children.[9] Symptoms of ADHD (which are also traits present to varying degrees in all children) make it challenging for a child to sit still, be attentive for sustained periods of time, listen to and follow instructions, or to resist impulses to shout out. Children with ADHD traits may also wander around the classroom or school building, struggle to complete school-work and need reminders to know what they are doing. This combination of characteristics is challenging for the child themselves; as they are not consciously able to control their behaviour without learning self-monitoring and regulatory strategies and are often criticised by adults and peers for their involuntary behaviour. It can also be disruptive to the usual mainstream classroom context, and there is a poor fit between the UK

For peer review only - http://bmjopen.bmj.com/site/about/guidelines.xhtml

mainstream Primary classroom environment and the behavioural characteristics and needs of children with impairing traits of ADHD. Development of an effective school-based intervention for ADHD that overcomes the limitations of previous interventions would be likely to improve health and social outcomes. The limitations of existing school-based non-pharmacological interventions are that they usually require delivery by trained clinicians, are complex and multi-component: targeting many potential deficits in every child regardless of their individual strengths and weaknesses. ADHD encompasses a wide range of core associated symptoms and functional issues that are seen in the classroom, and existing evidence is unclear about what works for whom, or which aspects of interventions are effective. In combination, this means existing interventions are not feasible or affordable for schools to routinely deliver, they are rarely rolled out in schools and are not followed with high fidelity. There are barriers to school staff identifying evidence-based practices and implementing these with high fidelity as well as adapting to the needs of the individual, and sustaining this over time. Reviews of existing school-based interventions for ADHD have highlighted the potential efficacy of such interventions (e.g.[10-13]), and so overcoming the limitations of existing interventions is crucial to provide an implementable, effective school-based intervention for ADHD.

We are in the process of developing an intervention from high-quality evidence, based on theory around behaviour change and ADHD and from a programme of development work including a Delphi survey of key stakeholders. We are following the Intervention Mapping approach to co-create the intervention with key stakeholders in order to adapt the format of evidence-based strategies, targeting both symptoms and associated functional impairments, to overcome key implementation issues in schools.[14] The result will be a 'toolkit' of strategies in a modular format, with some core components then a selection of optional modules focussing on a different core outcome, that is designed for Primary school staff to use when working with children with traits of ADHD: the Tools for Schools *FLEX* toolkit. The toolkit will have several modules targeting different outcomes that were shown to be of importance to key stakeholders in a Delphi survey;[15] including conflict with teachers and peers, paying attention, building self-esteem and improving planning and organisation.

For peer review only - http://bmjopen.bmj.com/site/about/guidelines.xhtml

Given the limitations of existing school-based interventions for ADHD, including limited evidence of effectiveness and difficulty evaluating them in methodologically-rigorous RCTs, it is clear that the toolkit will need to be developed in a way that ensures it is acceptable, useful and feasible for school staff. In addition to this, we must address key features that will allow successful trials of the toolkit in future powered trials so that the strength of evidence for its effectiveness is comparable to other treatments such as medication. Intervention development frameworks such as the Medical Research Council (MRC) complex intervention guidelines highlight the importance of the development and feasibility phases (rather than prematurely evaluating interventions in large randomised trials); carefully optimising protocols, working closely with the target populations, planning and testing implementation, and assessing the acceptability and feasibility of the toolkit are essential to develop an intervention that can later be tested for effectiveness. The development of the toolkit will therefore be iterative, trialling and modifying successive versions of the prototype in order to meet the study objectives.

In this protocol we outline plans to conduct the initial evaluation and further development of the Tools for Schools *FLEX* toolkit. As the toolkit is at an early stage of development, we plan to assess the acceptability of the toolkit, and the feasibility of implementing it within the Primary school context in the UK.

Objectives

The main aims of this feasibility study are to:

1. Refine the prototype *Tools for Schools FLEX* toolkit so that it is feasible and acceptable to implement in the school setting.
2. Establish whether a future definitive trial of efficacy is feasible.

Secondary aims are to:

For peer review only - http://bmjopen.bmj.com/site/about/guidelines.xhtml

a) Identify suitable outcome measures to assess core ADHD symptoms, child and teacher wellbeing, academic progress, and identify an appropriate primary outcome (from these) for a future definitive trial.

b) Develop and test a framework for costing the toolkit, and for assessing cost-effectiveness in a future definitive trial.

c) Assess whether observational measures of behaviour, classroom functioning and teacher-reported ADHD symptoms indicate improvement following use of the toolkit.

Methods and analysis

Design

A randomised iterative mixed methods case-series design will be used. Schools will be the units of randomisation, and will be randomised to the time (school term) when they will use the toolkit. The toolkit will be refined after each school has used it for one term, or more frequently if major issues are highlighted during a schools' use of the toolkit. Small, theory-based and data driven changes hypothesised to be relevant across the school context will be made, as well as reactive changes addressing barriers to implementation. Randomisation to using the toolkit in different school terms allows for inferences to be made as to whether the intervention is improving outcomes, with consistent improvement following the introduction of the core components enabling researchers to potentially rule out alternative explanations for behaviour change, such as improvement due to other support in place or differences in behaviour across the school year. If baseline symptoms and functioning remain on stable trajectories for children across schools, changing only when the intervention is introduced, this implies it is the intervention rather than an alternate factor stimulating the change. It is a commonly used design for single-subject analyses where the intervention cannot be withdrawn and re-implemented such as in an ABAB design.[16-18]

For peer review only - http://bmjopen.bmj.com/site/about/guidelines.xhtml

For peer review only

Eight schools will participate in total in two recruitment cohorts of four (in order to avoid later participating schools having to sign up to the study years in advance); each cohort will enter the study at a different time. Schools in each cohort will then be randomly assigned their individual baseline term in the study using a computerised random sequence generator (see Figure 1). The length of baseline period will be the same for each school (one school term), with schools within each cohort entering their baseline period in different school terms; the term in which the intervention is delivered is therefore staggered across the study (Figure 1). This design will allow for refining and adapting the toolkit and its implementation based on ongoing participant feedback, maximising the chances it has of being acceptable and feasible (and therefore suitable for further evaluation in a pilot and then definitive cluster randomised controlled trial) by the end of the study.

<INSERT FIGURE 1 HERE>

Patient and public involvement

A planning group of public and patient representatives, including people with ADHD (adults and children), parents of children with ADHD, school staff, education and health psychologists has been established to co-create the intervention. Input from this planning group has already extensively informed the design of the intervention and study, refining research questions, selecting outcome measures and considering participant burden. The group will also shape study dissemination plans. The planning group contains around 25 people, and will be consulted throughout the course of the study to make decisions about necessary modifications to the intervention, delivery or research design of a future evaluation. In relation to the study objectives, co-developing the intervention with key stakeholders in the planning group increases the chances of the toolkit being feasible and acceptable in the Primary school context. The group will actively collaborate to refine the toolkit in response to participant feedback to achieve the first aim. They will be consulted as to whether the feasibility study results indicate that a future trial is feasible, in relation to understanding the core features of a high-quality trial.

For peer review only - http://bmjopen.bmj.com/site/about/guidelines.xhtml

Regarding the secondary objectives, the planning group will use data collected to agree on the final primary outcome for a future definitive trial, as well as judging whether trialled measures are suitable measures to assess ADHD symptoms, academic attainment, child and teacher wellbeing. The planning group will contribute to the development of the costing framework and evaluate findings from study participants. Finally, the planning group will discuss the findings from observational measures in relation to the toolkit use, helping to understand why any emerging patterns in the data are seen.

Recruitment and Participants

Eight Primary schools, associated school staff and up to 32 children with impairing traits of ADHD (minimum 16 children) will participate in the study, and use the toolkit. All schools and all child participants will receive the intervention. Baseline data regarding treatment as usual will be collected in order to assess normal fluctuations in symptoms and response to any school-implemented treatment as usual during the baseline phase. Baseline data will not be used to exclude participants who have unstable profiles, rather to greater understand the nature of symptom and impairment fluctuation over time.

Recruitment will be through opportunistic sampling, utilising existing contacts from the planning group such as local Educational Psychology teams, and other education networks in the South West. At the time of writing, four schools have consented to participate and, given the demand for support managing children with ADHD in school, we anticipate no problems recruiting a total of eight schools. This sample of eight mainstream primary schools in the South West of England will allow sufficient variety to purposively sample schools to include a minimum of four in areas of high socioeconomic disadvantage, based on the selection of schools with an index of multiple deprivation above the regional mean. The intervention needs to be able to respond to the range of children with traits of ADHD, including those who are often unrecognised or less likely to access other support, such as girls,[19] therefore eligible schools will have at least one female pupil meeting eligibility criteria

For peer review only - http://bmjopen.bmj.com/site/about/guidelines.xhtml

and we will aim to recruit a diverse sample of children with differing ethnicities. A sample of eight schools also allows for the intervention periods to be staggered across the study and successive iterations of the toolkit. All participating schools will use the toolkit in the study for one school term.

Study participants include the school's senior leadership or head teacher, who will consent to schools' participation; the school special educational needs (and disabilities) co-ordinator (SENCo), teachers and teaching assistants (TAs) working with eligible children, eligible children with impairing traits of ADHD, and their parents.

School and school staff consent

Each school's senior leadership team will consent to school's participation. They will nominate a mental health lead, who is likely to be the SENCo (as children with ADHD and associated difficulties currently fall under their remit in the UK) and will be referred to as such henceforth. Decisions around recruitment and eligibility criteria have been made in close consultation with the study planning group.

As part of the condition of each schools' participation, the senior leadership team will be asked to ensure that the SENCo and potentially eligible teachers and TAs (those working with recruited children) are supportive of and potentially willing to take part in the study.

Identification and recruitment of eligible children and families

Eligible children will either have a clinical diagnosis of ADHD (as reported by the school or parents), or high symptom levels and will be aged 4 to 10 (school years Reception-5) at the first baseline assessment to ensure they will be in primary school for the study duration. Children with ADHD may either have not yet received a formal diagnosis, or their family may not wish to pursue a diagnosis, therefore, children with high symptom levels will be eligible. Teachers and SENCos will identify students who meet criteria for this indicated group using the Strengths and Difficulties Questionnaire hyperactivity-inattention subscale and the impact supplement.[20] Teachers of

For peer review only - http://bmjopen.bmj.com/site/about/guidelines.xhtml

For peer review only

potentially eligible children will be asked by the SENCo to complete this brief screening measure, and if this indicates that a child may have probable ADHD (teacher-reported symptoms being ≥6 and impact ≥1), their parent will also be asked to complete the screen in order to ensure that impairments are noted across more than one setting.[2, 21] Should these combined ratings indicate 'probable' ADHD according to the validated algorithm, the child will be eligible for inclusion in the study. Probable ADHD is assigned if the parent-report hyperactivity/inattention score is ≥7 and an impact score of 2 is given, or if the hyperactivity/inattention score is ≥9 and an impact score of at least one is reported, and the teacher criteria above are met (see sdqinfo.org for syntax).

SENCos will make initial contact with parents of children to clarify eligibility, to provide the study information and to establish whether families are interested in participation in principle. Willing families will be asked to consent to having their details passed to the research team, who will obtain written informed consent from parents and assent from children to participate. Should an individual child not meet eligibility criteria but their parents and teacher feels they would benefit from participation, this will be considered on a case-by-case basis. As the intervention is embedded as part of usual school practice, the planning group has indicated that ongoing assent should then be assumed for children through data collection unless the child indicates distress or reluctance.

Sample size

The planning group has advised that to minimise burden of data collection on teachers, each class teacher should have a maximum of one child participant; however this may be revised during the course of the study, for example if a school feels strongly that several children in one class may benefit, or if two eligible children in the same class also have TA support and the teacher wishes both to participate. Estimating that each of the eight participating schools will have 2-4 participating children and teachers, there will be a total of 16-32 child participants, 16-64 parents, and 16-64 teachers and TAs along with eight SENCos. The total sample size will depend on the distribution of

For peer review only - http://bmjopen.bmj.com/site/about/guidelines.xhtml

participating parents, teachers and teaching assistants and the distribution of eligible children across different classes within schools: Table 1 indicates the minimum and maximum sample sizes.

Given that the aim of the study is not to assess effectiveness or efficacy, the potential for the intervention to change between participants, and the case-series design, this study will not be powered to conduct a conventional between-individuals analysis. Instead, we will use multiple individual measures to analyse within-individual change and assess whether this replicates (at a

Table 1. Sample size minimum and maximum

**Participant type**	**Minimum number**	**Maximum number**
School	6	8
Children	16	32
Parents or carers	16	64 (i.e. 2 parents per child)
SENCos	8	8
Teachers	16	32
TAs	0	32

*maximum number based on every eligible school recruiting four pupils, each of which have a different class teacher. We anticipate actual numbers of TAs will be lower than the maximum.

descriptive level) across individuals. The pragmatic choice of eight schools allows for this purposive sampling, and both the minimum and maximum sample size of children is sufficient to address our objectives. Based on the mean number of children in eligible year groups in Devon schools, and a

For peer review only - http://bmjopen.bmj.com/site/about/guidelines.xhtml

conservative estimate of prevalence of ADHD in this age group of approximately 3%; it is expected that each school will have 4-8 eligible children, of whom we hope to recruit at least 50%. The recruitment target is feasible: school staff have indicated that a toolkit for ADHD would be perceived as useful and not burdensome as they believe it would be beneficial to their wider classroom management (the planning group highlight the data-collection burden as being the reason that teachers should only have one participating student per class).

An established set of study progression criteria are shown in Table 2 and will be used to assess success in terms of meeting the study aims. Criteria relate to the recruitment of schools (five or more being acceptable); recruitment of parents and teachers, and completion of study measures as well as some basic metrics of fidelity. The criteria will be reviewed throughout the study, and where individual criteria are Amber or Red, a core academics team meeting and a planning group meeting will be held to discuss, agree and then implement modifications to the study procedure or content.

Procedure

The study will run from September 2022 until January 2025. Data will primarily be collected at the participating schools, however interviews with parents and school staff may take place over the phone, via internet video call, at participants' homes, or in another location of participant's convenience. Baseline length will be one school term. During the baseline phase, the teacher will provide repeated measures of the primary quantitative outcomes: participating children's ADHD symptoms and classroom functioning. These will be collected every two weeks during the baseline period and in the intervention period. This detailed baseline data will capture normal fluctuations in symptoms across the term and response to any school-implemented 'treatment as usual' during the baseline phase.

Information on healthcare and education resource use will be collected from children, parents, and school staff for each child during the baseline period using a structured draft survey with open

For peer review only

For peer review only - http://bmjopen.bmj.com/site/about/guidelines.xhtml

questions to capture any resources not mentioned, and child quality of life will be reported by the child and their parent. Each school will then implement the intervention for one school term; two modules from the intervention will each be implemented for four weeks. There will be a ten-week follow-up period where detailed qualitative data will also be captured, including asking participants about changes in resource and service use relative to the information they provided at baseline.

Table 2. Study progression criteria

	Green: acceptable	Amber: discuss, modify	Red: redesign	Assessing
Recruitment of schools	6 or more	5	4 or fewer	Feasibility
Recruitment- teachers, children, parents	>65%	20-65%	<20%	Feasibility
Retention of schools, teachers, children and parents in study	>65%	40-65%	<40%	Feasibility
Training completed (teachers)	>90%	70-90%	<70%	Acceptability and feasibility
Introductory video watched (parents)	>50%	20-50%	<20%	Acceptability
Child strengths activity completed	>50%	20-50%	<20%	Acceptability
Adherence to digital Daily Report Card	>70%	50-70%	<50%	Acceptability

For peer review only - http://bmjopen.bmj.com/site/about/guidelines.xhtml

Teacher-completed measures	>70%	50-70%	<50%	Feasibility
Parent-completed measures	>50%	20-50%	<20%	Feasibility
Child-completed measures	> 70%	50-70%	<50%	Feasibility
Observational measures	>50%	20-50%	<20%	Feasibility
Attendance at toolkit-related meetings	>75%	40-75%	<40%	Acceptability
Percentage of occasions toolkit reportedly used as instructed	>75%	50-75%	<50%	Acceptability and feasibility
Follow-up measures completed	>50%	20-50%	N/A	Feasibility

Intervention delivery

The intervention is currently in development. Broadly, it will take the form of a digital toolkit of training and resource packages and behavioural strategies for non-specialists to use, organised within 'modules' that cover different classroom-based problems common to ADHD: we anticipate that each child participant will receive two modules. SENCos will have the role of co-ordinating and supporting teachers and TAs to deliver the toolkit in conjunction with support from parents at home. Supplementary Figure 1 illustrates the participant structure and roles within a school (see Supplementary material).

Intervention description

The Tools for Schools *FLEX* toolkit will primarily be a digital resource. The current logic model is shown in Figure 2 and the toolkit outline is shown in the Supplementary material (Supplementary Figure 2). The key goals of the toolkit that will foster sustainable behaviour change are to:

1. **Understand** the child and their communications

For peer review only - http://bmjopen.bmj.com/site/about/guidelines.xhtml

2. **Adapt** the classroom and school expectations

3. **Support** the child to:

A- reduce acute impairments

B- increase skills

C- improve self-identity

4. This results in the child being able to consciously monitor and self-regulate

5. **Sustain** and adapt across time

<INSERT FIGURE 2 HERE>

Table 3 shows the data collection schedule relative to the stage within the study. A full intervention description can be found in the Supplementary Material.

Qualitative data collection

Toolkit iteration feedback

The toolkit will go through several iterations, refined based on ongoing data collection and analysis. Flexibility allowing for this is a strength of the study design.[22] Brief semi-structured 'feedback sessions' will be held by phone or in person with all participating school staff and parents during the intervention period, in order to understand where modifications need to be made to the design, content and measures of the intervention. Example questions from the topic guide for teachers are included in the Supplementary Material. All changes made from the original prototype of the toolkit will be recorded in a change log, including the reason for the change, when the change was made and what aspects of the toolkit the change relates to. The planning group and the study advisory group will convene to discuss feedback or major suggested modifications, and to review minor changes in the change log.

For peer review only - http://bmjopen.bmj.com/site/about/guidelines.xhtml

Process evaluation

During the ten-week follow-up period, detailed qualitative data will be collected to assess acceptability, perceived usefulness, fidelity and implementation of the intervention, exploring in more depth what participants did and did not do and explanations of their actions, with questions similar to those shown above. Focus groups comprising the constellation of all school staff involved with one child will be held for all participants. This format is familiar to school staff as professional discussions often take a 'case conference' format. Interviews by phone or an online platform such as zoom will be conducted with a purposive sample of half of the parents, using the same criteria detailed above (i.e. at least 50% from areas with a deprivation index above the regional mean) and aiming to attain a balance of male and female parents.

For peer review only

For peer review only - http://bmjopen.bmj.com/site/about/guidelines.xhtml

Table 3. Data collection schedule and toolkit implementation timeline

School terms	Baseline term												
**Week**	**1**	**2**	**3**	**4**	**5**	**6**	**7**	**8**	**9**	**10**	**11**	**12**	**13**
**Toolkit steps**													
Know ADHD											X	X	X
Know me											X	X	X
Understand motivations and develop targets													X
Establish digital school-home notebook													
Map targets to modules and choose strategies													
Implement strategies (module 1)													
Implement strategies (module 2)													
**Study measures (data collection)**													
Measures of ADHD symptoms	X		X		X		X		X		X		X
Measures of classroom functioning	X		X		X		X		X		X		X
Toolkit iteration feedback (qualitative)													
Process evaluation: fidelity, acceptability, perceived usefulness													
Child dimensional psychopathology	X											X	
Child-reported satisfaction with school	X											X	
Teacher wellbeing	X											X	
Healthcare and education resource use	X											X	

School terms	Intervention term												
**Week**	**1**	**2**	**3**	**4**	**5**	**6**	**7**	**8**	**9**	**10**	**11**	**12**	**13**
**Toolkit steps**													
Know ADHD													
Know me													
Understand motivations and develop targets	X	X											
Establish digital school-home notebook	X	X											
Map targets to modules and choose strategies	X	X											
Implement strategies (module 1)		X	X	X	X	X	X						
Implement strategies (module 2)								X	X	X	X	X	X
**Study measures (data collection)**													
Measures of ADHD symptoms		X		X		X		X		X		X	
Measures of classroom functioning		X		X		X		X		X		X	
Toolkit iteration feedback (qualitative)					X								X
Process evaluation: fidelity, acceptability, perceived usefulness													
Child dimensional psychopathology						X							X
Child-reported satisfaction with school						X							X
Teacher wellbeing						X							X
Healthcare and education resource use													

School terms	Follow-up term												
**Week**	**1**	**2**	**3**	**4**	**5**	**6**	**7**	**8**	**9**	**10**	**11**	**12**	**13**
**Toolkit steps**													
Know ADHD													
Know me													
Understand motivations and develop targets													
Establish digital school-home notebook													
Map targets to modules and choose strategies													
Implement strategies (module 1)													
Implement strategies (module 2)													
**Study measures (data collection)**													
Measures of ADHD symptoms	X			X			X			X			X
Measures of classroom functioning	X			X			X			X			X
Toolkit iteration feedback (qualitative)													
Process evaluation: fidelity, acceptability, perceived usefulness	X	X	X	X	X	X	X	X	X	X			
Child dimensional psychopathology											X		
Child-reported satisfaction with school											X		
Teacher wellbeing											X		
Healthcare and education resource use	X												X

For peer review only

Child quality of life	x										x															x													x

Note: Supplementary material contains information on the Toolkit steps

For peer review only - http://bmjopen.bmj.com/site/about/guidelines.xhtml

All children will be invited to an activity- based interview with the option of a paired interview, where they can bring an accompanying family member of their choosing. Paired interviews can reduce stress, mitigate child protection risks and reduce issues of running a focus group where children in the school with traits of ADHD are identified to one another. The activity interviews will be designed with the planning group and piloted with children with ADHD, for example these may involve collecting objects of different colours or taking photos around the school to represent answers to questions (such as *can you show me where you like to go if you feel you need to calm down? What are your favourite things about school? What do you not like about school?)*, and using these objects or photos as prompts to discuss thoughts. All participants in the process evaluation will be asked what they think the most important outcome would be for a future hypothetical trial of the toolkit, and why.

Adverse effects of intervention

It is possible that the intervention will lead to adverse effects or negative impacts on participating children, such as feeling singled out due to the intervention leading to social exclusion from the peer group.[23] The study planning group will contribute to identifying potential adverse effects *(as of March 21 social exclusion is the most prominent concern*), and qualitative data will be collected from participants at the feedback and process evaluation stages to assess perceptions of negative impacts of the intervention. Modifications will be made as needed in relation to this through consultation with the planning group and the academic advisory team.

Quantitative measures

There are two quantitative outcome measures that will be assessed in order to: 1) determine which would be most suitable as the primary outcome in a future trial and 2) evaluate whether they make sense and are relevant to those completing the measures. **ADHD symptoms** will be measured using the Strengths and Weaknesses of ADHD symptoms and Normal-behaviour (SWAN) questionnaire,

For peer review only - http://bmjopen.bmj.com/site/about/guidelines.xhtml

based on preferences of the planning group who considered several measures of ADHD symptoms,[24, 25] reported by teachers; and **classroom functioning** measured using the Social Skills Improvement System,[26] that captures social, academic and competing problem behaviours in the classroom environment, reported by parents and teachers. These will be measured every 2 weeks during the baseline and intervention period and every 3 weeks during the follow-up period.

Other quantitative measures will be captured to assess whether there is indication of some improvement in the key areas the toolkit aims to target. Measures relating to children include **child dimensional psychopathology**, assessed using the teacher and parent-reported Strengths and Difficulties Questionnaire,[21] and **child-reported satisfaction with school** using the How I Feel About My School measure.[27] **Teacher wellbeing** will be measured using the Warwick-Edinburgh mental wellbeing scale 14-item Teacher survey [28] and the Maslach Burnout Inventory- General Survey.[29, 30] These will be collected at the beginning and end of the baseline period, at the end of the intervention term and at the end of the follow-up term.

Methods for collecting data on **healthcare and education resource use** and **health-related quality of life** will also be established and tested. Child quality of life (child-report and parent-proxy) will be measured using the Child Health Utility for Economic Evaluation (CHU9D).[31] A tool to accurately capture education and healthcare resource use will be developed for the study, drawing on the Client Service Receipt Inventory [32] and measures in the Database of Instruments for Resource Use Management Repository. Participants will complete this at baseline and follow-up, and provide qualitative information on changes in resource use during the follow-up process evaluation. A data collection form will also capture data on resource use and costs associated with the set-up and delivery of the intervention.

Observational measures of child behaviour in the classroom will also be tested throughout the study. These include but are not limited to the Classroom Observation Code,[33] and the Teacher-Pupil Observation Tool.[34]

For peer review only - http://bmjopen.bmj.com/site/about/guidelines.xhtml

Analysis

Aim 1. To assess whether the toolkit is feasible and acceptable for schools to implement , we will use the definition of acceptability proposed by Sekhon et al (2017): "a multi-faceted construct that reflects the extent to which people delivering or receiving a healthcare intervention consider it to be appropriate, based on anticipated or experienced cognitive and emotional responses to the intervention". Qualitative data from both the toolkit iteration interviews and the process evaluation interviews and focus groups will be analysed using the Framework method and Thematic analysis.[35] An initial coding framework will be developed after reading and re-reading transcripts. Deductive codes derived from the research questions and inductive codes within the data will be identified. This framework will be applied independently to three transcripts; discrepancies in coding will be discussed and revisions made to the framework which will then be applied to the remaining transcripts, with further discussion and revision where necessary.

Responses relating to each component of the toolkit will be synthesised in a framework in order to assess the acceptability of the evolving and final prototype; specifically to assess whether the components and the overall toolkit are considered appropriate to support children with ADHD in school. In addition, acceptability of the toolkit will be quantitatively assessed against the study progression criteria that relate to completion of each component of the toolkit, attendance at toolkit-related meetings, and the percentage of occasions the participant reports using the toolkit as instructed. The qualitative findings will be used to explain and further explore quantitative results in a mixed methods synthesis. Modifications will be made iteratively as findings emerge, and by the end of the study each indicator should be in the "green" range in order to indicate that the toolkit is acceptable.

Aim 2. To establish whether a future trial of efficacy is feasible, data on the research process and participant compliance will be evaluated. The quantitative data on recruitment and retention of participants, completion of measures by each participant group (parents, child, teacher, observed)

For peer review only

and adherence to the intervention protocol will be assessed relative to the relevant progression criteria described in Table 2. These have been informed by existing school-based trials.[36, 37] As above, qualitative findings from the process evaluation and toolkit iteration feedback will be integrated to explain quantitative findings. Should all relevant progression criteria be rated "green", a future trial of efficacy will be considered feasible. Should criteria be amber or red as the study progresses, potential mitigating modifications will be discussed, such as removing the requirement for parents to actively engage with the toolkit.

Sub-aim a: In order to identify suitable outcome measures to assess core ADHD symptoms, child and teacher wellbeing and identify an appropriate primary outcome for a future definitive trial the acceptability and feasibility of using the individual measures will be evaluated following the steps above and in relation to the threshold set in the progression criteria. Measures will be considered suitable if there is a high level of compliance with completion (i.e. each meet the green criteria), and qualitative findings indicate no major barriers to completing the measure on repeated occasions. Should measures be identified as unsuitable between toolkit iterations, new measures will be identified with the planning group and the data collection plan modified. For example, if the ADHD symptoms measure is too burdensome to complete as frequently as required, other briefer ADHD symptom scales will be explored, or the data collection frequency may be modified. The two versions of the CHU9-D will be compared to assess whether child-report is sufficient. To **identify an appropriate primary outcome for a future trial**, participants' qualitative responses to direct questions regarding what they think the most important outcome should be (and why) collected during the process evaluation will be synthesised. Findings will be discussed with the planning group and advisory team prior to deciding on the final primary outcome measure.

Sub-aim b. In order to test a framework for costing the toolkit, and for assessing cost-effectiveness in a future definitive trial, the detailed information collected from the draft framework will be assessed following baseline data collection, and modifications made to the framework where free-

For peer review only - http://bmjopen.bmj.com/site/about/guidelines.xhtml

text responses indicate additional resources not captured in the draft framework. The process evaluation interviews and focus group data will also contribute to understanding changes in resource use, which will be aligned with the follow-up responses regarding resource use in order to ascertain whether the framework is adequate and sensitive to changes in resource use.

The data captured on a bespoke form relating to the costs of setting up and delivering the toolkit will be assessed to expand the framework for accurately costing the toolkit's delivery and potential savings, and the costs of delivering the prototype intervention will be calculated (including costs for staff time, training and delivery of components). This framework will be revised in line with study findings with the aid of health economists, with a final framework developed by the end of the study that would allow for cost-effectiveness to be formally assessed in a future definitive trial.

Sub-aim c. Assess whether measures of behaviour, classroom functioning and teacher-reported ADHD symptoms indicate improvement following use of the toolkit. **To explore whether there is indication that the toolkit may be efficacious**, quantitative data will be visually analysed within schools and participants, describing the nature of change over time and in relation to the intervention, following recommendations.[22] Non-parametric analysis of the data will be considered, for example by calculating Tau-U statistics. As the toolkit will be refined between schools' participating, it would be irrelevant to compare measures across individuals who received different iterations of the toolkit; the focus will therefore be on assessing whether there is initial indication of improvement in outcomes, and whether any effect is replicated across individuals. Comparisons between the magnitude of change seen between those who receive earlier and later iterations of the toolkit will be carried out as it is anticipated that later iterations, modified to be more acceptable and feasible, would result in greater evidence of behaviour change. This will be an exploratory analysis. The core and target behaviours chosen across study participants will also be explored in a descriptive manner in order to assess the use of the behaviour web, targets and modules.

For peer review only - http://bmjopen.bmj.com/site/about/guidelines.xhtml

Missing, unused and spurious data and inclusion in analysis

All data will be utilised in analysis. Patterns of missing quantitative data will be evaluated as part of assessing the feasibility and acceptability of the intervention. Missing data could relate to the feasibility and acceptability of the intervention, or the utility and acceptability of the measure itself (i.e. Aim 1 and sub-aim 1), and so through the qualitative data collection we will aim to elicit how patterns of missing data relate to our aims and make modifications accordingly.

Ethics and dissemination

Ethics and governance approval

Ethical approval has been awarded by the University of Exeter College of Medicine and Health Research Ethics Committee (Ref Jan22/B/300). Informed consent will be sought from all adult participants (and by parents for child participants). Children will provide assent and ongoing assent will be judged by the research team throughout. Should it become clear that a child does not want to continue to participate in data collection or the study, withdrawal will be discussed with parents and school staff. Should a child express that they wish to stop during a single instance of data collection, this will be stopped for that day. The child's feelings will be respected and discussed with the child, their parents and school staff about their future involvement. Ultimately, decisions will be made in line with the best interests of the child. All data collected will comply with General Data Protection Regulation (GDPR) data protection regulations, being stored on secure servers and only accessible by the research team. Some data will be shared as part of the intervention, for example teachers' ratings of ADHD symptoms will be viewable by the SENCo and express permission will be sought for any planned data sharing between participants. Participants will be asked whether their data can be made available following the study for future analysis or related research. Those that consent will contribute their data to a fully anonymised dataset accessible to bona fide researchers.

For peer review only - http://bmjopen.bmj.com/site/about/guidelines.xhtml

There are several operational issues and associated risks that are involved in performing this study. It is possible that the intervention will lead to adverse outcomes [23] such as study children feeling singled-out from their peers due to the intervention. The planning group will discuss anticipated potential adverse outcomes due to the intervention, and qualitative feedback will be sought from participants both during their use of the intervention and in the follow-up period. Further risks (aside from those detailed in progression criteria in Table 2) have been identified and mitigation plans detailed (see Supplementary material).

Dissemination

Participants who consent to their anonymised data being shared for future research purposes will have their data archived in an open access dataset. The results from the study will be presented at relevant national and international conferences (e.g. the ADHD World Congress, Eunethydis, the Association for Child and Mental Health national conferences). Separate publications will describe: the iterative development process, the main outcomes of the case-series study, development of the resource use tool, and an in-depth qualitative analysis of data collected during the iteration and process evaluation. A detailed dissemination plan and documents will be developed with the planning group and will include blogs and podcasts, recorded and live talks and events and engagement activities to reach a broader audience of interested stakeholders and policymakers.

Declarations

Data Statement

Not applicable at this time as this is a study protocol. All participants will be asked for consent to store and share their data in a publically-accessible anonymised dataset.

For peer review only - http://bmjopen.bmj.com/site/about/guidelines.xhtml

Author Contributions

AR, DM and TF conceptualised the work; AR, BD, DM, RH, ESB, LP, TF, JK designed and drafted the research programme, led by AR. AR drafted the manuscript, and BD, DM, RH, ESB, LP, TF and JK critically revised the final manuscript.

Acknowledgements

Additional contribution was made by Suzie Holt, Eleanor Bryant, Becky Gudka and Charlotte Kelman of the Exeter University ChYMe (Children and Young People's Mental Health (ChYMe) research collaboration. We would also like to thank the members of the Tools for Schools planning group for their ongoing collaboration (https://blogs.exeter.ac.uk/toolsforschools/about-us/planning-group/).

Competing Interests

All authors declare that they have no competing interests other than those disclosed in the funding section below.

Funding

This report is independent research supported by the National Institute for Health Research NIHR Advanced Fellowship - Stage 2, Dr Abigail Russell, grant ref NIHR300591. The views expressed in this publication are those of the author(s) and not necessarily those of the NHS, the National Institute for Health Research or the Department of Health and Social Care. Tamsin Ford receives funding from Place2Be, a children's mental health charity (grant ref: N/A).

References

1. Parliamentary House of Commons. Children and young people's mental health - policy, services, funding and education (07196), 2017.

For peer review only - http://bmjopen.bmj.com/site/about/guidelines.xhtml

2. American Psychiatric Association. Diagnostic and statistical manual of mental disorders (DSM-5®): American Psychiatric Pub 2013.
3. Tarver J, Daley D, Sayal K. Attention-deficit hyperactivity disorder (ADHD): an updated review of the essential facts. *Child Care Health Dev* 2014;40(6):762-74. doi: 10.1111/cch.12139 [published Online First: 2014/04/15]
4. Faraone SV, Asherson P, Banaschewski T, et al. Attention-deficit/hyperactivity disorder. *Nature Reviews Disease Primers* 2015:15020. doi: 10.1038/nrdp.2015.20
5. Shaw M, Hodgkins P, Caci H, et al. A systematic review and analysis of long-term outcomes in attention deficit hyperactivity disorder: effects of treatment and non-treatment. *BMC medicine* 2012;10(1):99.
6. Gore FM, Bloem PJ, Patton GC, et al. Global burden of disease in young people aged 10–24 years: a systematic analysis. *The Lancet* 2011;377(9783):2093-102.
7. Telford C, Green C, Logan S, et al. Estimating the costs of ongoing care for adolescents with attention-deficit hyperactivity disorder. *Social psychiatry and psychiatric epidemiology* 2013;48(2):337-44.
8. Fleming M, Fitton CA, Steiner MFC, et al. Educational and Health Outcomes of Children Treated for Attention-Deficit/Hyperactivity Disorder. *JAMA pediatrics* 2017;171(7):e170691. doi: 10.1001/jamapediatrics.2017.0691 [published Online First: 2017/05/02]
9. Greene RW, Beszterczey SK, Katzenstein T, et al. Are students with ADHD more stressful to teach? Patterns of teacher stress in an elementary school sample. *Journal of Emotional and Behavioral Disorders* 2002;10(2):79-89.
10. Moore DA, Russell AE, Matthews J, et al. School-based interventions for attention-deficit/hyperactivity disorder: A systematic review with multiple synthesis methods. *Review of Education* 2018;6(3):209-63.
11. Veenman B, Luman M, Oosterlaan J. Efficacy of behavioral classroom programs in primary school. A meta-analysis focusing on randomized controlled trials. *PloS one* 2018;13(10):e0201779.
12. Fabiano GA, Pyle K. Best Practices in School Mental Health for Attention-Deficit/Hyperactivity Disorder: A Framework for Intervention. *School Mental Health* 2019;11(1):72-91. doi: 10.1007/s12310-018-9267-2
13. Wiener J. The role of school psychologists in supporting adolescents with ADHD. *Canadian Journal of School Psychology* 2020;35(4):299-310.
14. Kok G, Gottlieb NH, Peters G-JY, et al. A taxonomy of behaviour change methods: an Intervention Mapping approach. *Health Psychology Review* 2016;10(3):297-312. doi: 10.1080/17437199.2015.1077155
15. Perry RC, Ford TJ, O'Mahen H, et al. Prioritising Targets for School-Based ADHD Interventions: A Delphi Survey. *School Mental Health* 2021:1-15.
16. Kratochwill TR, Levin JR. Enhancing the scientific credibility of single-case intervention research: Randomization to the rescue. 2014
17. Morgan DL, Morgan RK. Multiple-baseline designs. *Single-case research methods for the behavioral and health sciences* 2009:125-61.
18. Kratochwill TR, Hitchcock J, Horner R, et al. Single-case designs technical documentation. *What works clearinghouse* 2010
19. Russell A, Ford T, Russell G. Barriers and predictors of medication use for childhood ADHD: findings from a UK population-representative cohort. *Social Psychiatry and Psychiatric Epidemiology* 2019:1-10.
20. Hall CL, Guo B, Valentine AZ, et al. The validity of the Strengths and Difficulties Questionnaire (SDQ) for children with ADHD symptoms. *PLOS ONE* 2019;14(6):e0218518. doi: 10.1371/journal.pone.0218518

For peer review only - http://bmjopen.bmj.com/site/about/guidelines.xhtml

21. Goodman R, Ford T, Simmons H, et al. Using the Strengths and Difficulties Questionnaire (SDQ) to screen for child psychiatric disorders in a community sample. *The British journal of psychiatry* 2000;177(6):534-39.
22. Morley S. Single case methods in clinical psychology: a practical guide: Routledge 2017.
23. Pfiffner LJ, DuPaul GJ. Possible adverse side effects of school behavioral interventions. *The ADHD Report* 2018;26(1):10-13.
24. Swanson JM, Schuck S, Porter MM, et al. Categorical and dimensional definitions and evaluations of symptoms of ADHD: history of the SNAP and the SWAN rating scales. *The International journal of educational and psychological assessment* 2012;10(1):51.
25. Brites C, Salgado-Azoni C, Ferreira T, et al. Development and applications of the SWAN rating scale for assessment of attention deficit hyperactivity disorder: a literature review. *Brazilian Journal of Medical and Biological Research* 2015;48(11):965-72.
26. Gresham F, Elliott SN. Social skills improvement system (SSIS) rating scales: San Antonio, TX: Pearson Education Inc 2007.
27. Allen K, Marlow R, Edwards V, et al. 'How I Feel About My School': The construction and validation of a measure of wellbeing at school for primary school children. *Clinical child psychology and psychiatry* 2018;23(1):25-41.
28. Tennant R, Hiller L, Fishwick R, et al. The Warwick-Edinburgh mental well-being scale (WEMWBS): development and UK validation. *Health and Quality of life Outcomes* 2007;5(1):1-13.
29. Langballe EM, Falkum E, Innstrand ST, et al. The factorial validity of the Maslach Burnout Inventory–General Survey in representative samples of eight different occupational groups. *Journal of Career Assessment* 2006;14(3):370-84.
30. Maslach C, Jackson S. Leiter MP Maslach burnout inventory manual. *Palo Alto: California Consulting Psychological Press Inc* 1996
31. Stevens K. Valuation of the child health utility 9D index. *Pharmacoeconomics* 2012;30(8):729-47.
32. Chisholm D, Knapp MRJ, Knudsen HC, et al. Client socio-demographic and service receipt inventory–European version: development of an instrument for international research: EPSILON Study 5. *The British Journal of Psychiatry* 2000;177(S39):s28-s33.
33. Abikoff H, Gittelman-Klein R, Klein DF. Validation of a classroom observation code for hyperactive children. *Journal of Consulting and Clinical Psychology* 1977;45(5):772.
34. Martin P, Daley D, Hutchings J, et al. The teacher-pupil observation tool (T-POT) development and testing of a New classroom observation measure. *School Psychology International* 2010;31(3):229-49.
35. Gale NK, Heath G, Cameron E, et al. Using the framework method for the analysis of qualitative data in multi-disciplinary health research. *BMC medical research methodology* 2013;13(1):1-8.
36. Pfiffner LJ, Rooney M, Haack L, et al. A randomized controlled trial of a school-implemented school–home intervention for attention-deficit/hyperactivity disorder symptoms and impairment. *Journal of the American Academy of Child & Adolescent Psychiatry* 2016;55(9):762-70.
37. Ford T, Hayes R, Byford S, et al. The effectiveness and cost-effectiveness of the Incredible Years® Teacher Classroom Management programme in primary school children: results of the STARS cluster randomised controlled trial. *Psychological medicine* 2019;49(5):828-42.

Figure legends

Figure 1. Case series design outline

Key- grey, baseline; green, intervention; blue, follow-up

For peer review only - http://bmjopen.bmj.com/site/about/guidelines.xhtml

Figure 2. Intervention Draft Logic Model

For peer review only

For peer review only - http://bmjopen.bmj.com/site/about/guidelines.xhtml

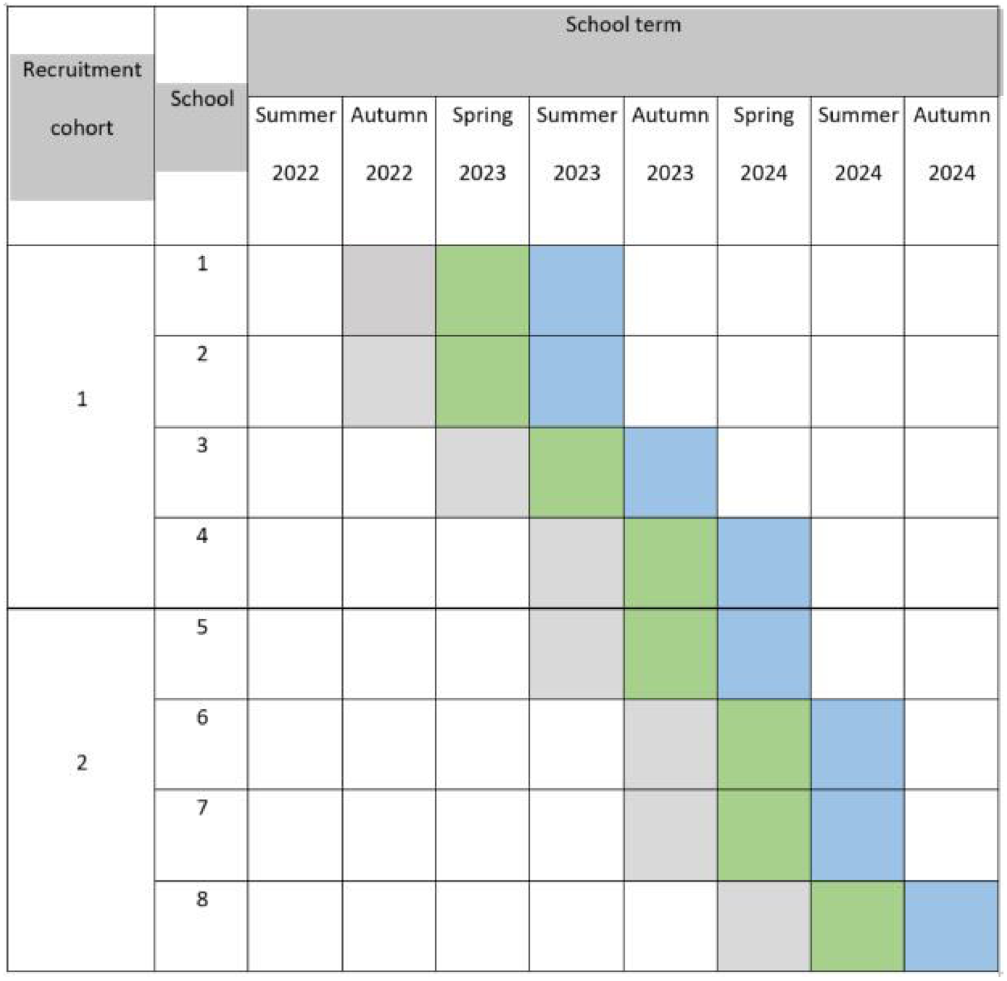

Figure 1. Case series design outline
Key- grey, baseline; green, intervention; blue, follow-up

52x51mm (300 x 300 DPI)

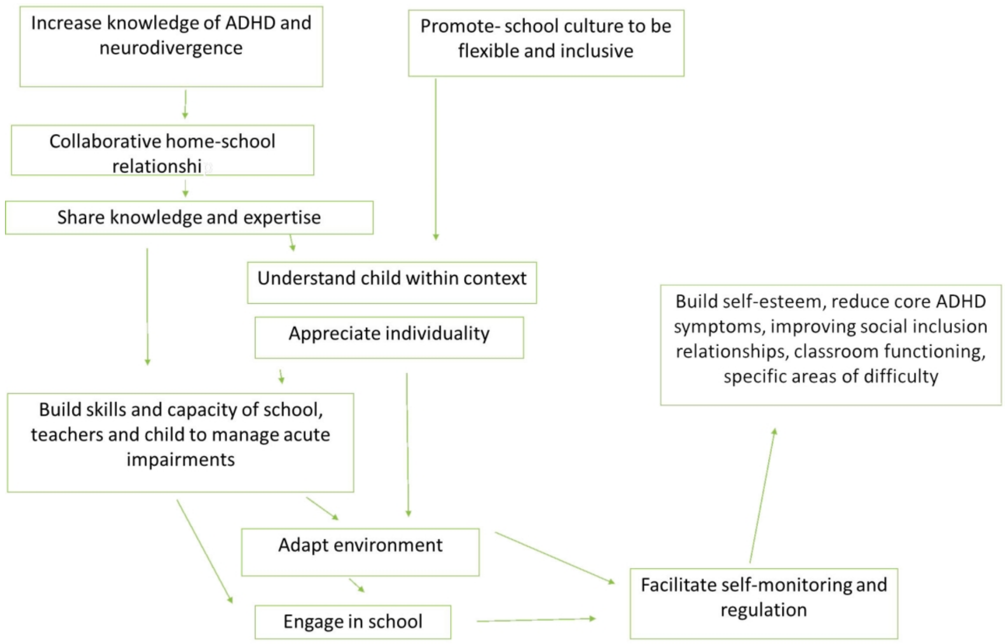

Figure 2. Intervention Draft Logic Model

169x109mm (300 x 300 DPI)

Protocol for an iterative case-series study to investigate the feasibility and acceptability of a school-based intervention for children with traits of ADHD: Supplementary Material

Full intervention description

The Tools for Schools *FLEX* toolkit will comprise a digital resource of training and resource packages and behavioural strategies organised within 'modules' that cover different classroom-based problems common to ADHD. See the main article for information on the key goals of the toolkit. Supplementary Figure 1 illustrates the participant structure and roles within a school for delivery of the toolkit.

Supplementary Figure 1. Structure of individuals within schools and participant roles in case series study

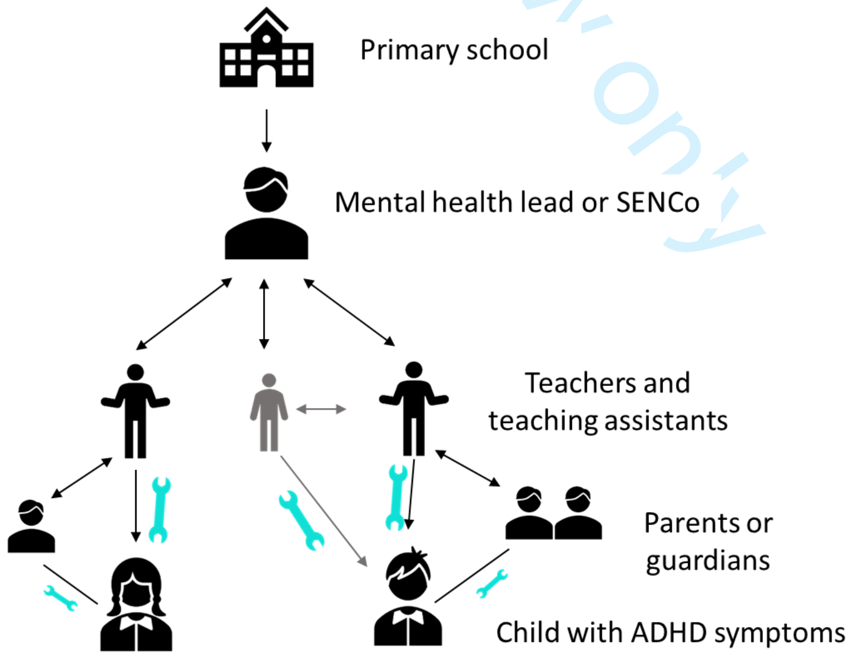

For peer review only - http://bmjopen.bmj.com/site/about/guidelines.xhtml

Delivery of the intervention comprises a series of steps, as indicated in the toolkit outline in Supplementary Figure 2, and described below.

Supplementary Figure 2. Outline of the *FLEX* Toolkit

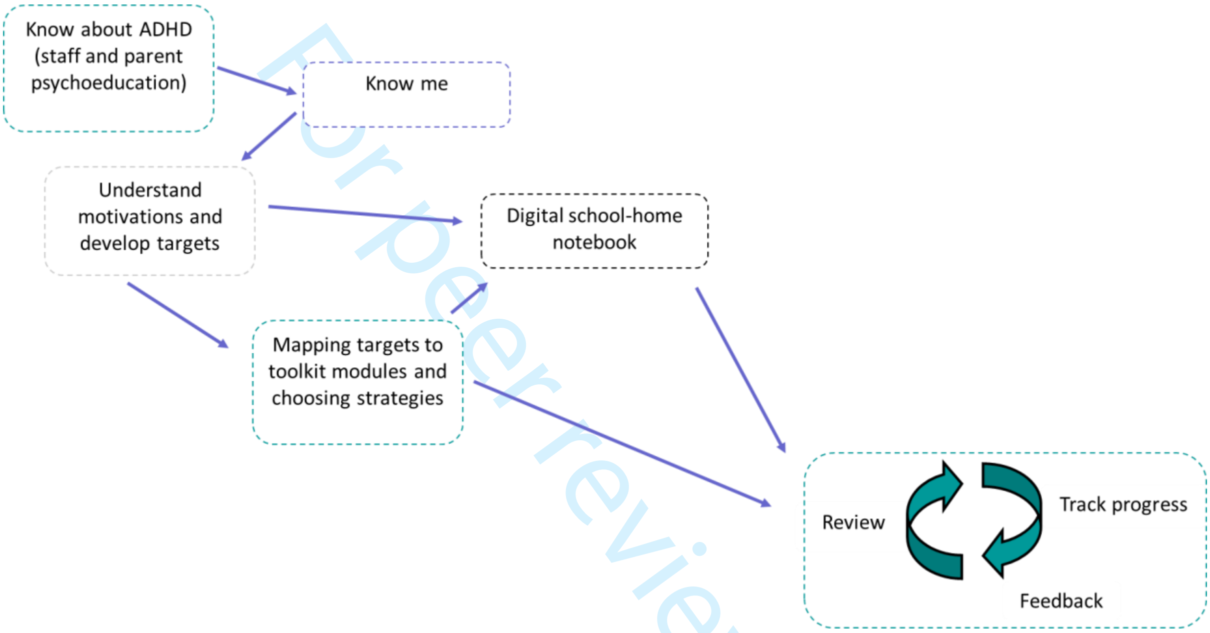

Step 1: Know ADHD

Purpose: Tailored knowledge of ADHD and relevant theory, psychoeducation. Putting staff and parents on equal footing, helping to address power dynamics between school and home and recognise expertise of each party

The toolkit will begin with online psychoeducation training for school staff and parents. This will cover ADHD as well as training on implementing the toolkit itself. Videos from people with ADHD, parents and school staff about their experiences will be included.

Key things the Know ADHD training will cover:

- ADHD is not an issue of morality or simply a deficit in attention
- Involvement of dopamine in the developing brain

For peer review only - http://bmjopen.bmj.com/site/about/guidelines.xhtml

- Executive functions and ADHD
- Delay aversion and ADHD
- Other theories about how ADHD traits come about
- Gender differences
- Sensory processing
- The variation between children with ADHD: every child with ADHD is different and unique, and their traits will affect them in different ways
- Growing up with traits of ADHD

Schools will also be asked to plan and deliver activities to promote inclusivity and the concepts of embracing individual differences and diversity at the outset of the study, through a participatory action research approach. Senior leadership and the SENCo will be asked to reflect on their school culture and inclusivity of school policies, make changes to these with the aim of improving flexibility around children with ADHD and their individual needs, and reflect on and evaluate these changes [23]. This might involve participating in national events such as Neurodiversity Celebration Week, having a school assembly or applying for the ADHD Foundation "ADHD friendly schools award" for example. Each school (senior leadership team [SLT] and SENCo) will decide on whether they want to implement any whole-school measures, and what form these will take. They will be supported by the research team but this is not an essential component of the toolkit, rather an exploratory component that will need to be led by each school themselves to be implementable or useful in future versions of the toolkit. We will use the Participatory Action Research cycle of Plan, Act, Evaluate, Reflect to structure this and provide a short instruction booklet as this component of the toolkit. We ask the SLT to consider how their school could be more inclusive for neurodivergent children (including asking for input from children and others in the school) and plan how they could change this. Then they would implement the new ideas (act) for a given period of time; evaluate the changes by seeking staff and student views, and reflect on whether they were successful before implementing further changes. No data will be collected at awareness events or other activities the

For peer review only - http://bmjopen.bmj.com/site/about/guidelines.xhtml

SLT decide to enact in this step unless they collect it for their own evaluative purposes. Staff will be asked to feedback about this during qualitative data collection instances. We anticipate researching the mechanisms of this potential component in further depth in separate research if participants report using this, implementing it and finding this component potentially useful.

Step 2: Know me (the child)

Purpose: Children with ADHD are more able to engage with something if it interests them. They often feel misunderstood by others and this activity is intended to allow staff to understand and empathise with the child, seeing things from their perspective. Children may not be able to verbalise their emotions and feelings and understanding how they behave when happy, sad, angry or excited is an important component of functional behaviour analysis and behaviour change approaches. This activity aims to draw these out for each individual.

Children will complete a strengths-focussed activity with the teacher or TA to identify strengths or hobbies that could be used to personalise the intervention and to help school staff understand the child from their perspective. This activity will be bespoke depending on what the child wants and likes to do and could be delivered to a whole class, but might include making a film or animation, creating a story or photo board, or building a virtual version of oneself on a computer game.

Step 3: Understand motivations and develop targets

Purpose of step: Sharing knowledge and expertise between school and home, focussing on understanding antecedents and consequences of behaviour in order to understand how best to modify. Mutually deciding on which targets are most important (and the relationships between potential targets)

At the end of the baseline term and the beginning of the intervention term, SENCos, teachers and other relevant staff will work alongside the child and family to complete a functional behaviour analysis of two problem areas where the child is currently struggling. We have chosen to constrain this to two areas in order to provide focus during the use of the toolkit in one school term for

For peer review only - http://bmjopen.bmj.com/site/about/guidelines.xhtml

For peer review only

research purposes, and to support those testing the toolkit to identify the key areas for improvement and therefore observable change. Understanding the antecedents and consequences of behaviour will be crucial to identify where to intervene and to understand which modules and strategies from the toolkit are likely to be useful. The meetings will result in identifying two core behaviours that will be transformed into the intervention "toolkit targets". The core problems will be operationalised into tangible and achievable goals (e.g. stay in seat for 5 minutes, with the number of minutes increasing as the child meets the target) over the course of two meetings.

The form of these meetings may vary depending on parent engagement and preferences. One option that will be piloted is inviting families and children to join these meetings via videoconference. This would allow the child to feel involved in the process if they wish to engage, but also provides them with the option to withdraw from the conversation. Through discussions with the planning group, parents identified that they wish to be consulted and to bring their perspective to these meetings, however they may also find such meetings are difficult emotionally, as the focus will be on their child's difficulties. As such, parents may feel more empowered or comfortable joining from their home environment. Alternately, parents and children may wish to attend these meetings in person at school. Parents will make the decision as to where/through what medium the meeting takes place.

Step 4: Digital school-home notebook

Purpose: Establishing and maintaining positive communication between teachers, TAs and parents. Research shows that conflictual and negative relationships between school and home are common for children with ADHD. Breaking negative communication cycles and establishing positive communication will improve sharing of information and ideas between home and school, and better support the child across contexts.

A communication platform for regular contact between staff and parents will be established once the child's toolkit targets have been agreed. This was initially intended to take the form of a digital

For peer review only - http://bmjopen.bmj.com/site/about/guidelines.xhtml

daily report card (dDRC) with staff updating home daily on the child's progress with their targets, and parents reinforcing this at home. The purpose of this component is to develop habits of positive communication about progress and child strengths, rather than to function as a "behaviour log" or to report incidents during school.

The frequency and nature of communication using the notebook will be flexible to suit the needs of the individual child and participants surrounding them; the DRC format will be proposed as the suggested model as this has the most existing evidence for effectiveness [24 25]. However, other forms of successful communication may already be in place between school and home, or daily communication may be considered burdensome and not beneficial to both parents and teachers for a wide variety of reasons. As such, staff and parents will be asked to mutually agree on frequency of communication using the digital school-home notebook, and this will be reviewed and modified as required during the intervention period.

Step 5. Mapping targets to toolkit modules and choosing strategies

Purpose: identifying strategies within modules that are most likely to be effective based on functional behaviour analysis and understanding of the child

The SENCo and teacher will link the child's targets to the most pertinent modules and outcomes in the toolkit using a graphical map (currently called the behaviour web). This may be initially discussed in the two meetings to understand behaviour and develop targets, or may be decided following these meetings. The functional behaviour analysis will be drawn upon with a focus on the current consequences relating to the target behaviour, and alternate consequences that could encourage more adaptive behaviour.

The toolkit modules are based on individual ADHD-related outcomes selected by consensus in a Delphi study. They are:

For peer review only - http://bmjopen.bmj.com/site/about/guidelines.xhtml

1. Organising, planning and reminding (describe to differentiate from organising)->means self-monitoring and self-regulating
2. Paying attention
3. Feeling good (self-esteem, self-worth)
4. Belonging and relating (relationships)
5. Pausing for thought (inhibiting impulsive behaviour)
6. Channelling energy (hyperactivity)

Modules will contain a bank of approximately 10 non-pharmacological strategies that can be used at individual or classroom level to target the outcome, organised into domains such as "Behaviour management" "Training tools" and "Changing the classroom" through the co-creation process. The planning group have indicated that the focus of the toolkit should be on inclusivity: supporting children to remain in the classroom and allowing the child to access the same education and opportunities as their classmates. Strategies will be developed with this goal in mind. In addition, modules will contain resources on maintaining strategies once implemented.

Step 6. Implementing and reflecting on strategies

Purpose: produce improvement in target behaviours that can be maintained

Once teachers and SENCos have determined the modules to implement through consideration of the functional behaviour analysis and the child's agreed targets, the SENCo will support the teacher and TAs to select and implement strategies. We anticipate that one module will be implemented in each half-term of the intervention term, however there will not be a restriction on this, nor how many strategies within a module can be attempted; we anticipate that some strategies will have already been tried by a school or teacher, or it may be apparent that other strategies do not suit the child and so different strategies may be selected. Repeated measures of ADHD symptoms, classroom functioning, and measures specific to the outcomes relevant for each module will be collected from

For peer review only - http://bmjopen.bmj.com/site/about/guidelines.xhtml

teachers, TAs and children as appropriate (Table 3 shows the data collection schedule relative to the stage within the study).

Example questions for Toolkit iteration feedback interviews

1. *Could you tell me about how you have used the toolkit?*
2. *What have your experiences been of using the toolkit? Let's talk about it step-by-step so you can give me your reflections on each component. First, overall did you think the toolkit was useable in Primary school? Why/why not? And do you think other schools and families would find it acceptable? Why/why not? Do you think other schools and families would be able to implement it? Why/why not?*
3. *What did you like most about the toolkit? What did you like least? Could you tell me some more about that?*
4. *How was the Know ADHD component? Did you use it, what was good about it and what was difficult or challenging? What would help you to use it better in the future? Was there anything missing that you think is important for people to know about ADHD?*
5. *How was the Know Me component? What did you do with the child to complete this activity? What format did the child choose (e.g. video, storybook)? How did you feel about it, and how do you think the child felt about doing the activity? Did you find the product of this Know Me step was useful for the rest of the toolkit? Could you tell me why you think that? Would you make any changes to the Know Me step or instructions, and what would they be?*
6. *Who set up the meetings with school staff to assess and understand the two main problems? How did those meetings go and who was there? When did you have the meetings? What was good and bad about the meetings? Did you manage to define two target goals for the child? Were parents and the child involved (if so how)? Would you do anything differently if you did it again? Should the toolkit have different instructions? How did you understand the antecedents, behaviours and consequences of each behaviour? Who helped to figure those out, how and how long did it take? Was that too much time for you?*

For peer review only - http://bmjopen.bmj.com/site/about/guidelines.xhtml

Risks and mitigation plans

Risk 1. Schools are open but due to periods of Covid-related closure, child development and socialisation has been impacted.

Mitigation: as part of the toolkit development process we are liaising closely with primary schools, parents and teachers to monitor ongoing changes to school environments and cultures. We anticipate that, if anything, there will be greater demand for the toolkit if more pupils are struggling with relevant interpersonal and developmental challenges. There may be the need to adapt the focus of modules, for example, to support school attendance explicitly. Potential adaptations will be discussed with the academic advisory team and planning group before implementation.

Risk 2. Schools are closed due to ongoing Covid-19

Mitigation. The planning group has advised that many of the children meeting eligibility criteria for the study have attended school through prior lockdowns and closure periods due to their vulnerable status and additional needs. We will explore whether sufficient children and staff are attending school at the time of recruitment and data collection, and if so will continue as planned with implementation of the intervention. Data collection will be completed remotely through digital questionnaires and online video conferencing software meeting data protection requirements if external (i.e. research) staff are unable to access schools due to covid-19 lockdowns.

Risk 3. Unanticipated adverse effects of intervention

Mitigation: adverse effects will be monitored through qualitative data collection during the use of the intervention, and participating school staff will follow a defined procedure for reporting of negative impacts as soon as possible after they come to light. Should a child or other participant of the study report severe worsening of ADHD symptoms or distress, the PI will consult with clinically-trained members of the academic advisory board to decide on the most appropriate course of action. This may include: the clinical academic speaking directly to staff and families to ascertain

For peer review only - http://bmjopen.bmj.com/site/about/guidelines.xhtml

For peer review only

whether the adverse effects are caused by the intervention or research study or are external to this, the child and family withdrawing from the study, or bespoke plans being put in place to monitor and manage the situation. Should a child or family withdraw from the study due to this, their existing data will not be used unless express consent is given at the time of withdrawal, and they will be able to continue to access the prototype intervention (as long as the study team are confident that this is not the cause of adverse effects) if they wish.

For peer review only

For peer review only - http://bmjopen.bmj.com/site/about/guidelines.xhtml

SPIRIT

STANDARD PROTOCOL ITEMS: RECOMMENDATIONS FOR INTERVENTIONAL TRIALS

SPIRIT 2013 Checklist: Recommended items to address in a clinical trial protocol and related documents*

Section/item	Item No	Description
**Administrative information**		
Title	1	Descriptive title identifying the study design, population, interventions, and, if applicable, trial acronym
Trial registration	2a	Trial identifier and registry name. If not yet registered, name of intended registry
	2b	All items from the World Health Organization Trial Registration Data Set
Protocol version	3	Date and version identifier
Funding	4	Sources and types of financial, material, and other support
Roles and responsibilities	5a	Names, affiliations, and roles of protocol contributors
	5b	Name and contact information for the trial sponsor
	5c	Role of study sponsor and funders, if any, in study design; collection, management, analysis, and interpretation of data; writing of the report; and the decision to submit the report for publication, including whether they will have ultimate authority over any of these activities
	5d	Composition, roles, and responsibilities of the coordinating centre, steering committee, endpoint adjudication committee, data management team, and other individuals or groups overseeing the trial, if applicable (see Item 21a for data monitoring committee)
**Introduction**		
Background and rationale	6a	Description of research question and justification for undertaking the trial, including summary of relevant studies (published and unpublished) examining benefits and harms for each intervention
	6b	Explanation for choice of comparators
Objectives	7	Specific objectives or hypotheses
Trial design	8	Description of trial design including type of trial (eg, parallel group, crossover, factorial, single group), allocation ratio, and framework (eg, superiority, equivalence, noninferiority, exploratory)

For peer review only

Methods: Participants, interventions, and outcomes

Study setting	9	Description of study settings (eg, community clinic, academic hospital) and list of countries where data will be collected. Reference to where list of study sites can be obtained
Eligibility criteria	10	Inclusion and exclusion criteria for participants. If applicable, eligibility criteria for study centres and individuals who will perform the interventions (eg, surgeons, psychotherapists)
Interventions	11a	Interventions for each group with sufficient detail to allow replication, including how and when they will be administered
	11b	Criteria for discontinuing or modifying allocated interventions for a given trial participant (eg, drug dose change in response to harms, participant request, or improving/worsening disease)
	11c	Strategies to improve adherence to intervention protocols, and any procedures for monitoring adherence (eg, drug tablet return, laboratory tests)
	11d	Relevant concomitant care and interventions that are permitted or prohibited during the trial
Outcomes	12	Primary, secondary, and other outcomes, including the specific measurement variable (eg, systolic blood pressure), analysis metric (eg, change from baseline, final value, time to event), method of aggregation (eg, median, proportion), and time point for each outcome. Explanation of the clinical relevance of chosen efficacy and harm outcomes is strongly recommended
Participant timeline	13	Time schedule of enrolment, interventions (including any run-ins and washouts), assessments, and visits for participants. A schematic diagram is highly recommended (see Figure)
Sample size	14	Estimated number of participants needed to achieve study objectives and how it was determined, including clinical and statistical assumptions supporting any sample size calculations
Recruitment	15	Strategies for achieving adequate participant enrolment to reach target sample size

Methods: Assignment of interventions (for controlled trials)

Allocation:

Sequence generation	16a	Method of generating the allocation sequence (eg, computer-generated random numbers), and list of any factors for stratification. To reduce predictability of a random sequence, details of any planned restriction (eg, blocking) should be provided in a separate document that is unavailable to those who enrol participants or assign interventions

Allocation concealment mechanism	16b	Mechanism of implementing the allocation sequence (eg, central telephone; sequentially numbered, opaque, sealed envelopes), describing any steps to conceal the sequence until interventions are assigned
Implementation	16c	Who will generate the allocation sequence, who will enrol participants, and who will assign participants to interventions
Blinding (masking)	17a	Who will be blinded after assignment to interventions (eg, trial participants, care providers, outcome assessors, data analysts), and how
	17b	If blinded, circumstances under which unblinding is permissible, and procedure for revealing a participant's allocated intervention during the trial
**Methods: Data collection, management, and analysis**		
Data collection methods	18a	Plans for assessment and collection of outcome, baseline, and other trial data, including any related processes to promote data quality (eg, duplicate measurements, training of assessors) and a description of study instruments (eg, questionnaires, laboratory tests) along with their reliability and validity, if known. Reference to where data collection forms can be found, if not in the protocol
	18b	Plans to promote participant retention and complete follow-up, including list of any outcome data to be collected for participants who discontinue or deviate from intervention protocols
Data management	19	Plans for data entry, coding, security, and storage, including any related processes to promote data quality (eg, double data entry; range checks for data values). Reference to where details of data management procedures can be found, if not in the protocol
Statistical methods	20a	Statistical methods for analysing primary and secondary outcomes. Reference to where other details of the statistical analysis plan can be found, if not in the protocol
	20b	Methods for any additional analyses (eg, subgroup and adjusted analyses)
	20c	Definition of analysis population relating to protocol non-adherence (eg, as randomised analysis), and any statistical methods to handle missing data (eg, multiple imputation)
**Methods: Monitoring**		
Data monitoring	21a	Composition of data monitoring committee (DMC); summary of its role and reporting structure; statement of whether it is independent from the sponsor and competing interests; and reference to where further details about its charter can be found, if not in the protocol. Alternatively, an explanation of why a DMC is not needed

For peer review only - http://bmjopen.bmj.com/site/about/guidelines.xhtml

	21b	Description of any interim analyses and stopping guidelines, including who will have access to these interim results and make the final decision to terminate the trial
Harms	22	Plans for collecting, assessing, reporting, and managing solicited and spontaneously reported adverse events and other unintended effects of trial interventions or trial conduct
Auditing	23	Frequency and procedures for auditing trial conduct, if any, and whether the process will be independent from investigators and the sponsor
**Ethics and dissemination**		
Research ethics approval	24	Plans for seeking research ethics committee/institutional review board (REC/IRB) approval
Protocol amendments	25	Plans for communicating important protocol modifications (eg, changes to eligibility criteria, outcomes, analyses) to relevant parties (eg, investigators, REC/IRBs, trial participants, trial registries, journals, regulators)
Consent or assent	26a	Who will obtain informed consent or assent from potential trial participants or authorised surrogates, and how (see Item 32)
	26b	Additional consent provisions for collection and use of participant data and biological specimens in ancillary studies, if applicable
Confidentiality	27	How personal information about potential and enrolled participants will be collected, shared, and maintained in order to protect confidentiality before, during, and after the trial
Declaration of interests	28	Financial and other competing interests for principal investigators for the overall trial and each study site
Access to data	29	Statement of who will have access to the final trial dataset, and disclosure of contractual agreements that limit such access for investigators
Ancillary and post-trial care	30	Provisions, if any, for ancillary and post-trial care, and for compensation to those who suffer harm from trial participation
Dissemination policy	31a	Plans for investigators and sponsor to communicate trial results to participants, healthcare professionals, the public, and other relevant groups (eg, via publication, reporting in results databases, or other data sharing arrangements), including any publication restrictions
	31b	Authorship eligibility guidelines and any intended use of professional writers
	31c	Plans, if any, for granting public access to the full protocol, participant-level dataset, and statistical code

For peer review only

Appendices

Informed consent materials	32	Model consent form and other related documentation given to participants and authorised surrogates
Biological specimens	33	Plans for collection, laboratory evaluation, and storage of biological specimens for genetic or molecular analysis in the current trial and for future use in ancillary studies, if applicable

*It is strongly recommended that this checklist be read in conjunction with the SPIRIT 2013 Explanation & Elaboration for important clarification on the items. Amendments to the protocol should be tracked and dated. The SPIRIT checklist is copyrighted by the SPIRIT Group under the Creative Commons "Attribution-NonCommercial-NoDerivs 3.0 Unported" license.

For peer review only

BMJ Open

Protocol for an iterative case-series study to investigate the feasibility and acceptability of a school-based intervention for children with traits of ADHD

Journal:	*BMJ Open*
Manuscript ID	bmjopen-2022-065176.R1
Article Type:	Protocol
Date Submitted by the Author:	15-Nov-2022

Complete List of Authors:	Russell, Abigail; University of Exeter, Children and Young People's Mental Health Research Collaboration (ChYMe), College of Medicine and Health<br>Dunn, Barney ; University of Exeter, Department of Psychology<br>Hayes, Rachel; University of Exeter, Children and Young People's Mental Health Research Collaboration (ChYMe), College of Medicine and Health<br>Moore, Darren; University of Exeter, Graduate School of Education<br>Kidger, Judi; University of Bristol, Population Health Sciences, Bristol Medical School<br>Sonuga-Barke, Edmund; King's College London, Institute of Psychiatry, Psychology and Neuroscience<br>Pfiffner, Linda; University of California San Francisco, Department of Psychiatry<br>Ford, Tamsin; University of Cambridge, Department of Psychiatry

**Primary Subject Heading**:	Public health
Secondary Subject Heading:	Health services research, Mental health, Qualitative research, Research methods
Keywords:	Developmental neurology & neurodisability < PAEDIATRICS, Community child health < PAEDIATRICS, Child & adolescent psychiatry < PSYCHIATRY

SCHOLARONE™
Manuscripts

Protocol for an iterative case-series study to investigate the feasibility and acceptability of a school-based intervention for children with traits of ADHD

Abigail Emma Russell[1], Barney Dunn[2], Rachel Hayes[1], Darren Moore[3], Judi Kidger[4], Edmund Sonuga-Barke[5], Linda Pfiffner[6], and Tamsin Ford[7]

[1]Children and Young People's Mental Health Research Collaboration (ChYMe), College of Medicine and Health, University of Exeter, Exeter, UK

[2]Department of Psychology, University of Exeter, Exeter, UK

[3]Graduate School of Education, University of Exeter, Exeter, UK

[4]Population Health Sciences, Bristol Medical School, University of Bristol, Bristol, UK

[5]Institute of Psychiatry, Psychology and Neuroscience, King's College London, London, UK

[6]Department of Psychiatry, University of California San Francisco, San Francisco, USA

[7]Department of Psychiatry, University of Cambridge, Cambridge, UK

Corresponding author: Abigail E Russell, University of Exeter, College of Medicine and Health, South Cloisters, St Luke's Campus, Exeter, UK EX1 2LU. E-mail: a.e.russell@exeter.ac.uk

Word count: 6023

For peer review only

Abstract

Introduction: Attention deficit/hyperactivity disorder (ADHD) is a prevalent and impairing cluster of traits affecting 2-5% of children. These children are at risk of negative health, social and educational outcomes and often experience severe difficulties at school: effective psychosocial interventions are needed. There is mixed evidence for existing school-based interventions for ADHD, which are complex and resource-intensive, contradicting teachers' preferences for short, flexible strategies that suit a range of ADHD-related classroom-based problems. They are also poorly evaluated. In this study, a prototype intervention comprising a digital 'toolkit' of behavioural strategies will be tested and refined. We aim to refine the prototype so that its use is feasible and acceptable within school settings, and to establish whether a future definitive, appropriately powered, trial of efficacy is feasible. This novel iterative study aims to pre-emptively address implementation and evaluation challenges that have hampered previous randomised controlled trials of non-pharmacological interventions. **Methods and analysis**: A randomised iterative mixed methods case-series design will be used. Schools will be randomised to the time (school term) they implement the toolkit. Eight Primary schools and 16-32 children with impairing traits of ADHD will participate, along with school staff and parents. The toolkit will be refined after each term, or more frequently if needed. Small, theory-based and data driven changes hypothesised as relevant across school contexts will be made, as well as reactive changes addressing implementation barriers. Feasibility and acceptability will be assessed through quantitative and qualitative data collection and analyses in relation to study continuation criteria, and ADHD symptoms and classroom functioning will be tracked and visually evaluated to assess whether there are early indications of toolkit utility. **Ethics and dissemination**: Ethical approval has been obtained. Results will be presented in journal articles, conferences and through varied forms of media to reach policymakers, stakeholders and the public.

Keywords: ADHD; school-based intervention; behavioural strategies; case-series; feasibility; acceptability

For peer review only - http://bmjopen.bmj.com/site/about/guidelines.xhtml

Article Summary

Strengths and limitations of this study

- This study uses an iterative staggered design in order to refine an intervention throughout the research process.
- Measures of acceptability and feasibility of the intervention and of the ability to evaluate it in a rigorous trial setting will be reviewed against continuation criteria throughout the study.
- Multiple informants will provide relevant data, including school staff and parents, and data on both positive and adverse outcomes will be captured.
- However, the iterative nature of the study means that different participants will not receive the same intervention, therefore group-level effects cannot be delineated.
- Alternate analyses will be utilised to ascertain whether there are patterns of change in symptoms and functioning that replicate across individuals instead.

Introduction

Children and young people's mental health and neurodevelopment (including ADHD) is a government priority. In the 2017 green paper, the pivotal role of schools in supporting good mental health was acknowledged. By the end of 2023, >20% of schools across the UK will have a designated mental health lead with the aim of improving access to evidence-based treatments.[1] Cost-effective, implementable, sustainable evidence-based mental health interventions that can be delivered in schools are therefore urgently needed.

Attention deficit/hyperactivity disorder (ADHD) is a neurodevelopmental disorder affecting 2-5% of children, characterised by impairing levels of impulsivity, hyperactivity and/or inattention.[2] Multiple negative life course outcomes are associated with childhood ADHD including high rates of co-occurring mental disorders, accidents, poor educational and occupational attainment and antisocial behaviour.[3] Other neuropsychiatric disorders and unintentional injury are strongly associated with ADHD, which are the leading cause of years lost due to disability worldwide in 10-24 year olds.[4] Costs to the health, education and judicial systems, social services and economic loss are substantial; 24%

For peer review only - http://bmjopen.bmj.com/site/about/guidelines.xhtml

of the mean annual cost of £5,493 is attributable to National Health Service (NHS) resources.[5] Pharmacological treatment is available and effective for some, especially in acute management of symptoms, but is not appropriate, acceptable or tolerable for all children.[6] Response is often partial[7 8], with tolerance developing over several years.[9 10] Even with drug treatment, ADHD causes particular problems in school, resulting in the outcomes described above.[11] Although ADHD medications have been shown to be effective and safe for the treatment of ADHD in the shorter term[10], licensed ADHD medications are controlled drugs and in order to access these families must receive a clinical diagnosis of ADHD, waiting lists for which in the UK are currently backlogged. Medication is not appropriate or preferable for all children with ADHD, and in the UK is not recommended as a first-line treatment for children with traits that are not severe[12]; and non-pharmacological intervention options that can be used as a precursor, alternative or supplement to pharmacological treatments are important.

ADHD causes problems in the classroom for the child, the teacher and for other children.[13] Symptoms of ADHD (which are also traits present to varying degrees in all children) make it challenging for a child to sit still, be attentive for sustained periods of time, listen to and follow instructions, or to resist impulses to shout out. Children with ADHD traits may also wander around the classroom or school building, struggle to complete school-work and need reminders to know what they are doing. This combination of characteristics is challenging for the child themselves; as they are not consciously able to control their behaviour without learning self-monitoring and regulatory strategies and are often criticised by adults and peers for their involuntary behaviour. It can also be disruptive to the usual mainstream classroom context, and there is a poor fit between the UK mainstream Primary classroom environment and the behavioural characteristics and needs of children with impairing traits of ADHD. Development of an effective school-based intervention for ADHD that overcomes the limitations of previous interventions would be likely to improve health and social outcomes. The limitations of existing school-based non-pharmacological interventions are that they usually require delivery by trained clinicians, are complex and multi-component: targeting

For peer review only - http://bmjopen.bmj.com/site/about/guidelines.xhtml

many potential deficits in every child regardless of their individual strengths and weaknesses[14-16]. ADHD encompasses a wide range of core associated symptoms and functional issues that are seen in the classroom, and existing evidence is unclear about what works for whom, or which aspects of interventions are effective. In combination, this means existing interventions are not feasible or affordable for schools to routinely deliver, they are rarely rolled out in schools and are not followed with high fidelity. There are barriers to school staff identifying evidence-based practices and implementing these with high fidelity as well as adapting to the needs of the individual, and sustaining this over time. Reviews of existing school-based interventions for ADHD have highlighted the potential efficacy of such interventions (e.g.[17-20]), and so overcoming the limitations of existing interventions is crucial to provide an implementable, effective school-based intervention for ADHD.

We are in the process of developing an intervention from high-quality evidence, based on theory around behaviour change and ADHD and from a programme of development work including a Delphi survey of key stakeholders. We are following the Intervention Mapping approach[21 22] to co-create the intervention with key stakeholders in order to adapt the format of evidence-based strategies, targeting both symptoms and associated functional impairments, to overcome key implementation issues in schools.[23] Intervention Mapping has six steps, and the full process of the toolkit development will be detailed in a subsequent publication, however in brief these are: 1. Creating a logic model of the problem; 2. Defining programme outcomes and objectives and a logic model of change; 3. Programme design; 4. Programme production; 5. Programme implementation plan; and 6. Evaluation plan[22]. The result will be a 'toolkit' of strategies in a modular format, with some core components then a selection of optional modules focussing on a different core outcome, that is designed for Primary school staff to use when working with children with traits of ADHD: the Tools for Schools *FLEX* toolkit. The toolkit will have several modules targeting different outcomes that were shown to be of importance to key stakeholders in a Delphi survey;[24] including conflict with teachers and peers, paying attention, building self-esteem and improving planning and organisation.

For peer review only - http://bmjopen.bmj.com/site/about/guidelines.xhtml

Given the limitations of existing school-based interventions for ADHD, including limited evidence of effectiveness and difficulty evaluating them in methodologically-rigorous RCTs, it is clear that the toolkit will need to be developed in a way that ensures it is acceptable, useful and feasible for school staff. In addition to this, we must address key features that will allow successful trials of the toolkit in future powered trials so that the strength of evidence for its effectiveness is comparable to other treatments such as medication. Intervention development frameworks such as the Medical Research Council (MRC) complex intervention guidelines highlight the importance of the development and feasibility phases (rather than prematurely evaluating interventions in large randomised trials); carefully optimising protocols, working closely with the target populations, planning and testing implementation, and assessing the acceptability and feasibility of the toolkit are essential to develop an intervention that can later be tested for effectiveness. The development of the toolkit will therefore take an iterative case-series approach, trialling and modifying successive versions of the prototype in order to meet the study objectives. We have not included a control group or comparison group as the study does not aim to assess effectiveness.

In this protocol we outline plans to conduct the initial evaluation and further development of the Tools for Schools *FLEX* toolkit. As the toolkit is at an early stage of development, we plan to assess the acceptability of the toolkit, and the feasibility of implementing it within the Primary school context in the UK.

Objectives

The main aims of this feasibility study are to:

1. Refine the prototype *Tools for Schools FLEX* toolkit so that it is feasible and acceptable to implement in the school setting.
2. Establish whether a future definitive trial of efficacy is feasible.

Secondary aims are to:

For peer review only - http://bmjopen.bmj.com/site/about/guidelines.xhtml

a) Identify suitable outcome measures to assess core ADHD symptoms, child and teacher wellbeing, academic progress, and identify an appropriate primary outcome (from these) for a future definitive trial.

b) Develop and test a framework for costing the toolkit, and for assessing cost-effectiveness in a future definitive trial.

c) Assess whether observational measures of behaviour, classroom functioning and teacher-reported ADHD symptoms indicate improvement following use of the toolkit.

Methods and analysis

Design

A randomised iterative mixed methods case-series design will be used. Schools will be the units of randomisation, and will be randomised to the time (school term) when they will use the toolkit. The toolkit will be refined after each school has used it for one term, or more frequently if major issues are highlighted during a schools' use of the toolkit. Small, theory-based and data driven changes hypothesised to be relevant across the school context will be made, as well as reactive changes addressing barriers to implementation. Randomisation to using the toolkit in different school terms allows for inferences to be made as to whether the intervention is improving outcomes, with consistent improvement following the introduction of the core components enabling researchers to potentially rule out alternative explanations for behaviour change, such as improvement due to other support in place or differences in behaviour across the school year. If baseline symptoms and functioning remain on stable trajectories for children across schools, changing only when the intervention is introduced, this implies it is the intervention rather than an alternate factor stimulating the change. It is a commonly used design for single-subject analyses where the intervention cannot be withdrawn and re-implemented such as in an ABAB design.[25-27]

Eight schools will participate in total in two recruitment cohorts of four (in order to avoid later participating schools having to sign up to the study years in advance); each cohort will enter the

For peer review only - http://bmjopen.bmj.com/site/about/guidelines.xhtml

study at a different time. Schools in each cohort will then be randomly assigned their individual baseline term in the study using a computerised random sequence generator (see Figure 1). The length of baseline period will be the same for each school (one school term), with schools within each cohort entering their baseline period in different school terms; the term in which the intervention is delivered is therefore staggered across the study (Figure 1). This design will allow for refining and adapting the toolkit and its implementation based on ongoing participant feedback, maximising the chances it has of being acceptable and feasible (and therefore suitable for further evaluation in a pilot and then definitive cluster randomised controlled trial) by the end of the study.

<INSERT FIGURE 1 HERE>

Patient and public involvement

A planning group of public and patient representatives, including people with ADHD (adults [n=5] and children [n=9]), parents of children with ADHD [n=12], school staff [n=9], education and health psychologists [n=4] has been established to co-create the intervention. Input from this planning group has already extensively informed the design of the intervention and study, refining research questions, selecting outcome measures and considering participant burden. The group will also shape study dissemination plans. The planning group contains around 25 people, and will be consulted throughout the course of the study to make decisions about necessary modifications to the intervention, delivery or research design of a future evaluation. In relation to the study objectives, co-developing the intervention with key stakeholders in the planning group increases the chances of the toolkit being feasible and acceptable in the Primary school context. The group will actively collaborate to refine the toolkit in response to participant feedback to achieve the first aim. They will be consulted as to whether the feasibility study results indicate that a future trial is feasible, in relation to understanding the core features of a high-quality trial. The role of the planning group in co-production of the toolkit is outlined in further detail in the supplementary material.

For peer review only

Regarding the secondary objectives, the planning group will use data collected to agree on the final primary outcome for a future definitive trial, as well as judging whether trialled measures are suitable measures to assess ADHD symptoms, academic attainment, child and teacher wellbeing. The planning group will contribute to the development of the costing framework and evaluate findings from study participants. Finally, the planning group will discuss the findings from observational measures in relation to the toolkit use, helping to understand why any emerging patterns in the data are seen.

Recruitment and Participants

Eight Primary schools, associated school staff and up to 32 children with impairing traits of ADHD (minimum 16 children) will participate in the study, and use the toolkit. All schools and all child participants will receive the intervention. Baseline data regarding treatment as usual will be collected in order to assess normal fluctuations in symptoms and response to any school-implemented treatment as usual during the baseline phase. Baseline data will not be used to exclude participants who have unstable profiles, rather to greater understand the nature of symptom and impairment fluctuation over time.

Recruitment will be through opportunistic sampling, utilising existing contacts from the planning group such as local Educational Psychology teams, and other education networks in the South West. At the time of writing, four schools have consented to participate and, given the demand for support managing children with ADHD in school, we anticipate no problems recruiting a total of eight schools. This sample of eight mainstream primary schools in the South West of England will allow sufficient variety to purposively sample schools to include a minimum of four in areas of high socioeconomic disadvantage, based on the selection of schools with an index of multiple deprivation above the regional mean. The intervention needs to be able to respond to the range of children with traits of ADHD, including those who are often unrecognised or less likely to access other support, such as girls,[28] therefore eligible schools will have at least one female pupil meeting eligibility criteria

For peer review only - http://bmjopen.bmj.com/site/about/guidelines.xhtml

and we will aim to recruit a diverse sample of children with differing ethnicities. A sample of eight schools also allows for the intervention periods to be staggered across the study and successive iterations of the toolkit. All participating schools will use the toolkit in the study for one school term.

Study participants include the school's senior leadership or head teacher, who will consent to schools' participation; the school special educational needs (and disabilities) co-ordinator (SENCo), teachers and teaching assistants (TAs) working with eligible children, eligible children with impairing traits of ADHD, and their parents. Consent will be obtained by a member of the research team through meeting with potential participants face-to-face or online.

School and school staff consent

Each school's senior leadership team will consent to school's participation. They will nominate a mental health lead, who is likely to be the SENCo (as children with ADHD and associated difficulties currently fall under their remit in the UK) and will be referred to as such henceforth. Decisions around recruitment and eligibility criteria have been made in close consultation with the study planning group.

As part of the condition of each schools' participation, the senior leadership team will be asked to ensure that the SENCo and potentially eligible teachers and TAs (those working with recruited children) are supportive of and potentially willing to take part in the study.

Identification and recruitment of eligible children and families

Eligible children will either have a clinical diagnosis of ADHD (as reported by the school or parents), or high symptom levels and will be aged 4 to 10 (school years Reception-5) at the first baseline assessment to ensure they will be in primary school for the study duration. Children with ADHD may either have not yet received a formal diagnosis, or their family may not wish to pursue a diagnosis, therefore, children with high symptom levels will be eligible. There are no additional exclusion criteria. Teachers and SENCos will identify students who meet criteria for this indicated group using the Strengths and Difficulties Questionnaire hyperactivity-inattention subscale and the impact

For peer review only - http://bmjopen.bmj.com/site/about/guidelines.xhtml

supplement.[29] Teachers of potentially eligible children will be asked by the SENCo to complete this brief screening measure, and if this indicates that a child may have probable ADHD (teacher-reported symptoms being ≥6 and impact ≥1), their parent will also be asked to complete the screen in order to ensure that impairments are noted across more than one setting.[2 30] Should these combined ratings indicate ‘probable’ ADHD according to the validated algorithm, the child will be eligible for inclusion in the study. Probable ADHD is assigned if the parent-report hyperactivity/inattention score is ≥7 and an impact score of 2 is given, or if the hyperactivity/inattention score is ≥9 and an impact score of at least one is reported, and the teacher criteria above are met (see sdqinfo.org for syntax).

SENCos will make initial contact with parents of children to clarify eligibility, to provide the study information and to establish whether families are interested in participation in principle. Willing families will be asked to consent to having their details passed to the research team, who will obtain written informed consent from parents and assent from children to participate. Children will be informed of the broad purpose of the research through an age-appropriate information sheet and assent form, unless their parents request otherwise. Should an individual child not meet eligibility criteria but their parents and teacher feels they would benefit from participation, this will be considered on a case-by-case basis. As the intervention is embedded as part of usual school practice, the planning group has indicated that ongoing assent should then be assumed for children through data collection unless the child indicates distress or reluctance.

Sample size

The planning group has advised that to minimise burden of data collection on teachers, each class teacher should have a maximum of one child participant; however this may be revised during the course of the study, for example if a school feels strongly that several children in one class may benefit, or if two eligible children in the same class also have TA support and the teacher wishes both to participate. Estimating that each of the eight participating schools will have 2-4 participating

For peer review only - http://bmjopen.bmj.com/site/about/guidelines.xhtml

For peer review only

children and teachers, there will be a total of 16-32 child participants, 16-64 parents, and 16-64 teachers and TAs along with eight SENCos. The total sample size will depend on the distribution of participating parents, teachers and teaching assistants and the distribution of eligible children across different classes within schools: Table 1 indicates the minimum and maximum sample sizes.

Given that the aim of the study is not to assess effectiveness or efficacy, the potential for the intervention to change between participants, and the case-series design, this study will not be powered to conduct a conventional between-individuals analysis. Instead, we will use multiple individual measures to analyse within-individual change and assess whether this replicates (at a descriptive level) across individuals. The pragmatic choice of eight schools allows for this purposive sampling, and both the minimum and maximum sample size of children is sufficient to address our objectives. Based on the mean number of children in eligible year groups in Devon schools, and a conservative estimate of prevalence of ADHD in this age group of approximately 3%; it is expected that each school will have 4-8 eligible children, of whom we hope to recruit at least 50%. The recruitment target is feasible: school staff have indicated that a toolkit for ADHD would be perceived as useful and not burdensome as they believe it would be beneficial to their wider classroom management (the planning group highlight the data-collection burden as being the reason that teachers should only have one participating student per class).

For peer review only - http://bmjopen.bmj.com/site/about/guidelines.xhtml

Table 1. Sample size minimum and maximum

Participant type	Minimum number	Maximum number
School	6	8
Children	16	32
Parents or carers	16	64 (i.e. 2 parents per child)
SENCos	8	8
Teachers	16	32
TAs	0	32

*maximum number based on every eligible school recruiting four pupils, each of which have a different class teacher. We anticipate actual numbers of TAs will be lower than the maximum.

An established set of study progression criteria are shown in Table 2 and will be used to assess success in terms of meeting the study aims. Criteria relate to the recruitment of schools (five or more being acceptable); recruitment of parents and teachers, and completion of study measures as well as some basic metrics of fidelity. The criteria will be reviewed throughout the study, and where individual criteria are Amber or Red, a core academics team meeting and a planning group meeting will be held to discuss, agree and then implement modifications to the study procedure or content.

Procedure

The study will run from September 2022 until January 2025. Data will primarily be collected at the participating schools, however interviews with parents and school staff may take place over the phone, via internet video call, at participants' homes, or in another location of participant's convenience. Baseline length will be one school term. During the baseline phase, the teacher will provide repeated measures of the primary quantitative outcomes: participating children's ADHD

For peer review only - http://bmjopen.bmj.com/site/about/guidelines.xhtml

symptoms and classroom functioning. These will be collected every two weeks during the baseline period and in the intervention period. This detailed baseline data will capture normal fluctuations in symptoms across the term and response to any school-implemented 'treatment as usual' during the baseline phase.

Information on healthcare and education resource use will be collected from children, parents, and school staff for each child during the baseline period using a structured draft survey with open questions to capture any resources not mentioned, and child quality of life will be reported by the child and their parent. Any other support or treatment will not be altered during the study. Each school will then implement the intervention for one school term; two modules from the intervention will each be implemented for four weeks. There will be a ten-week

follow-up period where detailed qualitative data will also be captured, including asking participants about changes in resource and service use relative to the information they provided at baseline.

For peer review only

Table 2. Study progression criteria

	Green: acceptable	Amber: discuss, modify	Red: redesign	Assessing
Recruitment of schools	6 or more	5	4 or fewer	Feasibility
Recruitment- teachers, children, parents (considered as separate groups)	>65%	20-65%	<20%	Feasibility
Retention of schools, teachers, children and parents in study	>65%	40-65%	<40%	Feasibility
Training completed (teachers)	>90%	70-90%	<70%	Acceptability and feasibility
Introductory video watched (parents)	>50%	20-50%	<20%	Acceptability
Child strengths activity completed	>50%	20-50%	<20%	Acceptability
Adherence to digital Daily Report Card	>70%	50-70%	<50%	Acceptability
Teacher-completed measures	>70%	50-70%	<50%	Feasibility
Parent-completed measures	>50%	20-50%	<20%	Feasibility
Child-completed measures	> 70%	50-70%	<50%	Feasibility
Observational measures	>70%	50-70%	<50%	Feasibility
Attendance at toolkit-related meetings	>75%	40-75%	<40%	Acceptability
Percentage of occasions toolkit reportedly used as instructed	>75%	50-75%	<50%	Acceptability and feasibility

For peer review only - http://bmjopen.bmj.com/site/about/guidelines.xhtml

Follow-up qualitative data collection completed (for each type of respondent: school staff, parents, child)	>50%	20-50%	N/A	Feasibility

Intervention delivery

The intervention is currently in development. Broadly, it will take the form of a digital toolkit of training and resource packages and behavioural strategies for non-specialists to use, organised within 'modules' that cover different classroom-based problems common to ADHD: we anticipate that each child participant will receive two modules. SENCos will have the role of co-ordinating and supporting teachers and TAs to deliver the toolkit in conjunction with support from parents at home, and given agency over the duration, dose and discontinuation of strategies. Supplementary Figure 1 illustrates the participant structure and roles within a school (see Supplementary material).

Intervention description

The Tools for Schools *FLEX* toolkit will primarily be a digital resource. The current logic model is shown in Figure 2 and the toolkit outline is shown in the Supplementary material (Supplementary Figure 2). The key goals of the toolkit that will foster sustainable behaviour change are to:

1. **Understand** the child and their communications

2. **Adapt** the classroom and school expectations

3. **Support** the child to:

   A- reduce acute impairments

   B- increase skills

   C- improve self-identity

through providing strategies that change the environment and expectations around the child.

For peer review only - http://bmjopen.bmj.com/site/about/guidelines.xhtml

4. This results in the child being able to consciously monitor and self-regulate

5. **Sustain** and adapt across time

<INSERT FIGURE 2 HERE>

Table 3 shows the data collection schedule relative to the stage within the study. A full intervention description can be found in the Supplementary Material.

Qualitative data collection

Toolkit iteration feedback

The toolkit will go through several iterations, refined based on ongoing data collection and analysis. Flexibility allowing for this is a strength of the study design.[31] Brief semi-structured 'feedback sessions' will be held by phone or in person with all participating school staff and parents during the intervention period, in order to understand where modifications need to be made to the design, content and measures of the intervention. Example questions from the topic guide for teachers are included in the Supplementary Material. All changes made from the original prototype of the toolkit will be recorded in a change log, including the reason for the change, when the change was made and what aspects of the toolkit the change relates to. The planning group and the study advisory group will convene to discuss feedback or major suggested modifications, and to review minor changes in the change log.

Process evaluation

During the ten-week follow-up period, detailed qualitative data will be collected to assess acceptability, perceived usefulness, fidelity and implementation of the intervention, exploring in more depth what participants did and did not do and explanations of their actions, with questions similar to those shown above. Focus groups comprising the constellation of all school staff involved with one child will be held for all participants. This format is familiar to school staff as professional discussions often take a 'case conference'

For peer review only - http://bmjopen.bmj.com/site/about/guidelines.xhtml

format. Interviews by phone or an online platform such as zoom will be conducted with a purposive sample of half of the parents, using the same criteria detailed above (i.e. at least 50% from areas with a deprivation index above the regional mean) and aiming to attain a balance of male and female parents. Children will take part in ‘active interviews’ during the process evaluation in the follow-up period with a researcher (at home or at school), where they will have an informal conversation about their experiences of the study, if they recalled any new things their teacher tried with them, how they felt about their behaviour targets (if they knew about them), and how they found the research process.

For peer review only

Table 3. Data collection schedule and toolkit implementation timeline

School terms	Baseline term													Intervention term													Follow-up term												
**Week**	**1**	**2**	**3**	**4**	**5**	**6**	**7**	**8**	**9**	**10**	**11**	**12**	**13**	**1**	**2**	**3**	**4**	**5**	**6**	**7**	**8**	**9**	**10**	**11**	**12**	**13**	**1**	**2**	**3**	**4**	**5**	**6**	**7**	**8**	**9**	**10**	**11**	**12**	**13**
**Toolkit steps**																																							
Know ADHD											X	X	X																										
Know me											X	X	X																										
Understand motivations and develop targets													X	X	X																								
Establish digital school-home notebook														X	X																								
Map targets to modules and choose strategies														X	X																								
Implement strategies (module 1)															X	X	X	X	X	X																			
Implement strategies (module 2)																					X	X	X	X	X	X													
**Study measures (data collection)**																																							
Measures of ADHD symptoms	X		X		X		X		X		X		X		X		X		X		X		X		X		X			X			X			X			X
Measures of classroom functioning	X		X		X		X		X		X		X		X		X		X		X		X		X		X			X			X			X			X
Toolkit iteration feedback (qualitative)																		X								X													
Process evaluation: fidelity, acceptability, perceived usefulness																											X	X	X	X	X	X	X	X	X	X			
Child dimensional psychopathology and school functioning	X											X							X							X											X		
Child-reported satisfaction with school	X											X							X							X											X		
Teacher wellbeing	X											X							X							X											X		
Healthcare and education resource use	X											X															X												X

For peer review only

Child quality of life	X											X																X											X

Note: Supplementary material contains information on the Toolkit steps. Measures are: ADHD symptoms: the Strengths and Weaknesses of ADHD and Normal Behavior Scale (SWAN); Classroom functioning: problem behaviour subscale of the Social Skills Improvement System (SISS); Dimensional psychopathology and school functioning: the Strengths and Difficulties Questionnaire and the academic competence and social skills subscales of the SISS; Satisfaction with school: the How I Feel About My School questionnaire; Teacher wellbeing: the Warwick-Edinburgh mental wellbeing scale 14-item Teacher survey, and the Relationship with Work survey from the Maslach Burnout Inventory- General Survey, and the Teacher Sense of Efficacy Scale; healthcare and education resource use is a bespoke questionnaire available from the authors on request; and Child quality of life is measured by child-report and parent proxy Child Health Utility-9D.

For peer review only

All children will be invited to an activity- based interview with the option of a paired interview, where they can bring an accompanying family member of their choosing. Paired interviews can reduce stress, mitigate child protection risks and reduce issues of running a focus group where children in the school with traits of ADHD are identified to one another. The activity interviews will be designed with the planning group and piloted with children with ADHD, for example these may involve collecting objects of different colours or taking photos around the school to represent answers to questions (such as *can you show me where you like to go if you feel you need to calm down? What are your favourite things about school? What do you not like about school?)*, and using these objects or photos as prompts to discuss thoughts.

All participants in the process evaluation will be asked what they think the most important outcome would be for a future hypothetical trial of the toolkit, and why.

Adverse effects of intervention

It is possible that the intervention will lead to adverse effects or negative impacts on participating children, such as feeling singled out due to the intervention leading to social exclusion from the peer group.[32] The study planning group will contribute to identifying potential adverse effects *(as of March 21 social exclusion is the most prominent concern*), and qualitative data will be collected from participants at the feedback and process evaluation stages to assess perceptions of negative impacts of the intervention. Modifications will be made as needed in relation to this through consultation with the planning group and the academic advisory team.

Quantitative measures

There are two quantitative outcome measures that will be assessed in order to: 1) determine which would be most suitable as the primary outcome in a future trial and 2) evaluate whether they make sense and are relevant to those completing the measures. **ADHD symptoms** will be measured using the Strengths and Weaknesses of ADHD symptoms and Normal-behaviour (SWAN) questionnaire, based on preferences of the planning group who considered several measures of ADHD symptoms,[33]

For peer review only - http://bmjopen.bmj.com/site/about/guidelines.xhtml

[34] reported by teachers; and **classroom functioning problem behaviour** subscale measured using the Social Skills Improvement System (SISS),[35] that captures social, academic and competing problem behaviours in the classroom environment, reported by parents and teachers. These will be measured every 2 weeks during the baseline and intervention period and every 3 weeks during the follow-up period. All planned quantitative measures are detailed in a table in the Supplementary material.

Other quantitative measures will be captured to assess whether there is indication of some improvement in the key areas the toolkit aims to target. Measures relating to children include **child dimensional psychopathology**, assessed using the teacher and parent-reported Strengths and Difficulties Questionnaire,[30] and **child-reported satisfaction with school** using the How I Feel About My School measure.[36] **Teacher wellbeing** will be measured using the Warwick-Edinburgh mental wellbeing scale 14-item Teacher survey [37] the Teachers' Sense of Efficacy Scale,[38] and the Relationship with Work Survey from the Maslach Burnout Inventory- General Survey.[39 40] ; and the social skills and academic competence subscales from the SISS will be completed by teachers. These will be collected at the beginning and end of the baseline period, at the end of the intervention term and at the end of the follow-up term. Other module-specific measures which are not covered by the above measures will be administered depending on the modules used by each child, e.g. the Harter Self-Perception Profile for Children to measure self-esteem.[41]

Methods for collecting data on **healthcare and education resource use** and **health-related quality of life** will also be established and tested. Child quality of life (child-report and parent-proxy) will be measured using the Child Health Utility for Economic Evaluation (CHU9D).[42] A tool to accurately capture education and healthcare resource use will be developed for the study, drawing on the Client Service Receipt Inventory [43] and measures in the Database of Instruments for Resource Use Management Repository. Participants will complete this at baseline and follow-up, and provide qualitative information on changes in resource use during the follow-up process evaluation. A data

For peer review only - http://bmjopen.bmj.com/site/about/guidelines.xhtml

collection form will also capture data on resource use and costs associated with the set-up and delivery of the intervention.

Observational measures of child behaviour in the classroom will also be tested throughout the study. These include but are not limited to the Classroom Observation Code,[44] and the Teacher-Pupil Observation Tool.[45] Observations will be carried out at school by members of the research team, trained to an acceptable reliability (0.7 or higher).

All data will be collected online via secure servers, or on paper, and manual data entry will be double checked.

Analysis

Aim 1. To assess whether the toolkit is feasible and acceptable for schools to implement , we will use the definition of acceptability proposed by Sekhon et al (2017): "a multi-faceted construct that reflects the extent to which people delivering or receiving a healthcare intervention consider it to be appropriate, based on anticipated or experienced cognitive and emotional responses to the intervention". Qualitative data from both the toolkit iteration interviews and the process evaluation interviews and focus groups will be audio recorded, transcribed and analysed using the Framework method and Thematic analysis.[46] An initial coding framework will be developed after reading and re-reading transcripts. Deductive codes derived from the research questions and inductive codes within the data will be identified. This framework will be applied independently to three transcripts; discrepancies in coding will be discussed and revisions made to the framework which will then be applied to the remaining transcripts, with further discussion and revision where necessary.

Responses relating to each component of the toolkit will be synthesised in a framework in order to assess the acceptability of the evolving and final prototype; specifically to assess whether the components and the overall toolkit are considered appropriate to support children with ADHD in school. In addition, acceptability of the toolkit will be quantitatively assessed against the study progression criteria that relate to completion of each component of the toolkit, attendance at

For peer review only - http://bmjopen.bmj.com/site/about/guidelines.xhtml

toolkit-related meetings, and the percentage of occasions the participant reports using the toolkit as instructed. The qualitative findings will be used to explain and further explore quantitative results in a mixed methods synthesis. Modifications will be made iteratively as findings emerge, and by the end of the study each indicator should be in the "green" range in order to indicate that the toolkit is acceptable.

Aim 2. To establish whether a future trial of efficacy is feasible, data on the research process and participant compliance will be evaluated. The quantitative data on recruitment and retention of participants, completion of measures by each participant group (parents, child, teacher, observed) and adherence to the intervention protocol will be assessed relative to the relevant progression criteria described in Table 2. These have been informed by existing school-based trials.[47 48] As above, qualitative findings from the process evaluation and toolkit iteration feedback will be integrated to explain quantitative findings. Should all relevant progression criteria be rated "green", a future trial of efficacy will be considered feasible. Should criteria be amber or red as the study progresses, potential mitigating modifications will be discussed, such as removing the requirement for parents to actively engage with the toolkit.

Sub-aim a: In order to identify suitable outcome measures to assess core ADHD symptoms, child and teacher wellbeing and identify an appropriate primary outcome for a future definitive trial the acceptability and feasibility of using the individual measures will be evaluated following the steps above and in relation to the threshold set in the progression criteria. Measures will be considered suitable if there is a high level of compliance with completion (i.e. each meet the green criteria), and qualitative findings indicate no major barriers to completing the measure on repeated occasions. Should measures be identified as unsuitable between toolkit iterations, new measures will be identified with the planning group and the data collection plan modified. For example, if the ADHD symptoms measure is too burdensome to complete as frequently as required, other briefer ADHD symptom scales will be explored, or the data collection frequency may be modified. The two versions of the CHU9-D will be compared to assess whether child-report is sufficient. To **identify an**

For peer review only - http://bmjopen.bmj.com/site/about/guidelines.xhtml

appropriate primary outcome for a future trial, participants' qualitative responses to direct questions regarding what they think the most important outcome should be (and why) collected during the process evaluation will be synthesised. Findings will be discussed with the planning group and advisory team prior to deciding on the final primary outcome measure.

Sub-aim b. In order to test a framework for costing the toolkit, and for assessing cost-effectiveness in a future definitive trial, the detailed information collected from the draft framework will be assessed following baseline data collection, and modifications made to the framework where free-text responses indicate additional resources not captured in the draft framework. The process evaluation interviews and focus group data will also contribute to understanding changes in resource use, which will be aligned with the follow-up responses regarding resource use in order to ascertain whether the framework is adequate and sensitive to changes in resource use.

The data captured on a bespoke form relating to the costs of setting up and delivering the toolkit will be assessed to expand the framework for accurately costing the toolkit's delivery and potential savings, and the costs of delivering the prototype intervention will be calculated (including costs for staff time, training and delivery of components). This framework will be revised in line with study findings with the aid of health economists, with a final framework developed by the end of the study that would allow for cost-effectiveness to be formally assessed in a future definitive trial.

Sub-aim c. Assess whether measures of behaviour, classroom functioning and teacher-reported ADHD symptoms indicate improvement following use of the toolkit. **To explore whether there is indication that the toolkit may be efficacious**, quantitative data will be visually analysed within schools and participants, describing the nature of change over time and in relation to the intervention, following recommendations.[31] Non-parametric analysis of the data will be considered, for example by calculating Tau-U statistics. As the toolkit will be refined between schools' participating, it would be irrelevant to compare measures across individuals who received different iterations of the toolkit; the focus will therefore be on assessing whether there is initial indication of

For peer review only - http://bmjopen.bmj.com/site/about/guidelines.xhtml

improvement in outcomes, and whether any effect is replicated across individuals. Comparisons between the magnitude of change seen between those who receive earlier and later iterations of the toolkit will be carried out as it is anticipated that later iterations, modified to be more acceptable and feasible, would result in greater evidence of behaviour change. This will be an exploratory analysis. The core and target behaviours chosen across study participants will also be explored in a descriptive manner in order to assess the use of the behaviour web, targets and modules.

Missing, unused and spurious data and inclusion in analysis

All data will be utilised in analysis. Patterns of missing quantitative data will be evaluated as part of assessing the feasibility and acceptability of the intervention. Missing data could relate to the feasibility and acceptability of the intervention, or the utility and acceptability of the measure itself (i.e. Aim 1 and sub-aim 1), and so through the qualitative data collection we will aim to elicit how patterns of missing data relate to our aims and make modifications accordingly.

Ethics and dissemination

Ethics and governance approval

Ethical approval has been awarded by the University of Exeter College of Medicine and Health Research Ethics Committee (Ref Jan22/B/300). Informed consent will be sought from all adult participants (and by parents for child participants; an example information sheet and consent form is provided in the Supplementary Material). Children will provide assent and ongoing assent will be judged by the research team throughout. Should it become clear that a child does not want to continue to participate in data collection or the study, withdrawal will be discussed with parents and school staff. Should a child express that they wish to stop during a single instance of data collection, this will be stopped for that day. The child's feelings will be respected and discussed with the child, their parents and school staff about their future involvement. Ultimately, decisions will be made in line with the best interests of the child. All data collected will comply with General Data Protection Regulation (GDPR) data protection regulations, being stored on secure servers and only accessible by

For peer review only

the research team. Some data will be shared as part of the intervention, for example teachers' ratings of ADHD symptoms will be viewable by the SENCo and express permission will be sought for any planned data sharing between participants. Participants will be asked whether their data can be made available following the study for future analysis or related research. Those that consent will contribute their data to a fully anonymised dataset accessible to bona fide researchers. There are several operational issues and associated risks that are involved in performing this study. It is possible that the intervention will lead to adverse outcomes [32] such as study children feeling singled-out from their peers due to the intervention. The planning group will discuss anticipated potential adverse outcomes due to the intervention, and qualitative feedback will be sought from participants both during their use of the intervention and in the follow-up period. Further risks (aside from those detailed in progression criteria in Table 2) have been identified and mitigation plans detailed, as well as noting key study limitations (see Supplementary material).

Dissemination

Participants who consent to their anonymised data being shared for future research purposes will have their data archived in an open access dataset. The results from the study will be presented at relevant national and international conferences (e.g. the ADHD World Congress, Eunethydis, the Association for Child and Mental Health national conferences). Separate publications will describe: the iterative development process, the main outcomes of the case-series study, development of the resource use tool, and an in-depth qualitative analysis of data collected during the iteration and process evaluation; authorship will be determined using the ICJME criteria. A detailed dissemination plan and documents will be developed with the planning group and will include blogs and podcasts, recorded and live talks and events and engagement activities to reach a broader audience of interested stakeholders and policymakers.

For peer review only - http://bmjopen.bmj.com/site/about/guidelines.xhtml

Declarations

Data Statement

Not applicable at this time as this is a study protocol. All participants will be asked for consent to store and share their data in a publically-accessible anonymised dataset.

Should amendments be made to the study protocol after publication, we will advise the publishing journal and publish a note of amendment.

Author Contributions

AR, DM and TF conceptualised the work; AR, BD, DM, RH, ESB, LP, TF, JK designed and drafted the research programme, led by AR. AR drafted the manuscript, and BD, DM, RH, ESB, LP, TF and JK critically revised the final manuscript. This is protocol version 1.2, November 2022.

Acknowledgements

Additional contribution was made by Suzie Holt, Eleanor Bryant, Becky Gudka and Charlotte Kelman of the Exeter University ChYMe (Children and Young People's Mental Health (ChYMe) research collaboration. We would also like to thank the members of the Tools for Schools planning group for their ongoing collaboration (https://blogs.exeter.ac.uk/toolsforschools/about-us/planning-group/).

Competing Interests

All authors declare that they have no competing interests other than those disclosed in the funding section below.

Funding

This report is independent research supported by the National Institute for Health Research NIHR Advanced Fellowship - Stage 2, Dr Abigail Russell, grant ref NIHR300591. The views expressed in this publication are those of the author(s) and not necessarily those of the NHS, the National Institute for

For peer review only - http://bmjopen.bmj.com/site/about/guidelines.xhtml

Health Research or the Department of Health and Social Care. Tamsin Ford receives funding from Place2Be, a children's mental health charity (grant ref: N/A).

References

1. Parliamentary House of Commons. Children and young people's mental health - policy, services, funding and education (07196), 2017.

2. American Psychiatric Association. Diagnostic and statistical manual of mental disorders (DSM-5®): American Psychiatric Pub 2013.

3. Tarver J, Daley D, Sayal K. Attention-deficit hyperactivity disorder (ADHD): an updated review of the essential facts. *Child Care Health Dev* 2014;40(6):762-74. doi: 10.1111/cch.12139 [published Online First: 2014/04/15]

4. Gore FM, Bloem PJ, Patton GC, et al. Global burden of disease in young people aged 10–24 years: a systematic analysis. *The Lancet* 2011;377(9783):2093-102.

5. Telford C, Green C, Logan S, et al. Estimating the costs of ongoing care for adolescents with attention-deficit hyperactivity disorder. *Social psychiatry and psychiatric epidemiology* 2013;48(2):337-44.

6. Wehmeier PM, Dittmann RW, Banaschewski T. Treatment compliance or medication adherence in children and adolescents on ADHD medication in clinical practice: results from the COMPLY observational study. *ADHD Attention Deficit and Hyperactivity Disorders* 2015;7(2):165-74.

7. Taylor E. ADHD medication in the longer term. *Zeitschrift für Kinder-und Jugendpsychiatrie und Psychotherapie* 2019

8. Bates G. Drug treatments for attention-deficit hyperactivity disorder in young people. *Advances in psychiatric treatment* 2009;15(3):162-71.

9. Galvez-Contreras AY, Vargas-de la Cruz I, Beltran-Navarro B, et al. Therapeutic Approaches for ADHD by Developmental Stage and Clinical Presentation. *International Journal of Environmental Research and Public Health* 2022;19(19):12880.

For peer review only - http://bmjopen.bmj.com/site/about/guidelines.xhtml

10. Cortese S, Adamo N, Del Giovane C, et al. Comparative efficacy and tolerability of medications for attention-deficit hyperactivity disorder in children, adolescents, and adults: a systematic review and network meta-analysis. *The Lancet Psychiatry* 2018;5(9):727-38. doi: https://doi.org/10.1016/S2215-0366(18)30269-4

11. Fleming M, Fitton CA, Steiner MFC, et al. Educational and Health Outcomes of Children Treated for Attention-Deficit/Hyperactivity Disorder. *JAMA pediatrics* 2017;171(7):e170691. doi: 10.1001/jamapediatrics.2017.0691 [published Online First: 2017/05/02]

12. Excellence NIfHaC. Attention deficit hyperactivity disorder: diagnosis and management. *Attention deficit hyperactivity disorder: diagnosis and management* 2019

13. Greene RW, Beszterczey SK, Katzenstein T, et al. Are students with ADHD more stressful to teach? Patterns of teacher stress in an elementary school sample. *Journal of Emotional and Behavioral Disorders* 2002;10(2):79-89.

14. Fabiano GA, Pelham WE, Jr., Coles EK, et al. A meta-analysis of behavioral treatments for attention-deficit/hyperactivity disorder. *Clinical Psychology Review* 2009;29(2):129-40.

15. Richardson M, Moore DA, Gwernan-Jones R, et al. Non-pharmacological interventions for attention-deficit/hyperactivity disorder (ADHD) delivered in school settings: systematic reviews of quantitative and qualitative research. *Health Technology Assessment (Winchester, England)* 2015;19(45):1-470.

16. Catalá-López F, Hutton B, Núñez-Beltrán A, et al. The pharmacological and non-pharmacological treatment of attention deficit hyperactivity disorder in children and adolescents: a systematic review with network meta-analyses of randomised trials. *PloS one* 2017;12(7):e0180355.

17. Moore DA, Russell AE, Matthews J, et al. School-based interventions for attention-deficit/hyperactivity disorder: A systematic review with multiple synthesis methods. *Review of Education* 2018;6(3):209-63.

For peer review only - http://bmjopen.bmj.com/site/about/guidelines.xhtml

18. Veenman B, Luman M, Oosterlaan J. Efficacy of behavioral classroom programs in primary school. A meta-analysis focusing on randomized controlled trials. *PloS one* 2018;13(10):e0201779.

19. Fabiano GA, Pyle K. Best Practices in School Mental Health for Attention-Deficit/Hyperactivity Disorder: A Framework for Intervention. *School Mental Health* 2019;11(1):72-91.

20. Wiener J. The role of school psychologists in supporting adolescents with ADHD. *Canadian Journal of School Psychology* 2020;35(4):299-310.

21. Bartholomew LK, Parcel GS, Kok G. Intervention mapping: a process for developing theory and evidence-based health education programs. *Health education & behavior* 1998;25(5):545-63.

22. Eldredge LKB, Markham CM, Ruiter RA, et al. Planning health promotion programs: an intervention mapping approach: John Wiley & Sons 2016.

23. Kok G, Gottlieb NH, Peters G-JY, et al. A taxonomy of behaviour change methods: an Intervention Mapping approach. *Health Psychology Review* 2016;10(3):297-312. doi: 10.1080/17437199.2015.1077155

24. Perry RC, Ford TJ, O'Mahen H, et al. Prioritising Targets for School-Based ADHD Interventions: A Delphi Survey. *School Mental Health* 2021:1-15.

25. Kratochwill TR, Levin JR. Enhancing the scientific credibility of single-case intervention research: Randomization to the rescue. 2014

26. Morgan DL, Morgan RK. Multiple-baseline designs. *Single-case research methods for the behavioral and health sciences* 2009:125-61.

27. Kratochwill TR, Hitchcock J, Horner R, et al. Single-case designs technical documentation. *What works clearinghouse* 2010

28. Russell A, Ford T, Russell G. Barriers and predictors of medication use for childhood ADHD: findings from a UK population-representative cohort. *Social Psychiatry and Psychiatric Epidemiology* 2019:1-10.

For peer review only - http://bmjopen.bmj.com/site/about/guidelines.xhtml

29. Hall CL, Guo B, Valentine AZ, et al. The validity of the Strengths and Difficulties Questionnaire (SDQ) for children with ADHD symptoms. *PLOS ONE* 2019;14(6):e0218518. doi: 10.1371/journal.pone.0218518

30. Goodman R, Ford T, Simmons H, et al. Using the Strengths and Difficulties Questionnaire (SDQ) to screen for child psychiatric disorders in a community sample. *The British journal of psychiatry* 2000;177(6):534-39.

31. Morley S. Single case methods in clinical psychology: a practical guide: Routledge 2017.

32. Pfiffner LJ, DuPaul GJ. Possible adverse side effects of school behavioral interventions. *The ADHD Report* 2018;26(1):10-13.

33. Swanson JM, Schuck S, Porter MM, et al. Categorical and dimensional definitions and evaluations of symptoms of ADHD: history of the SNAP and the SWAN rating scales. *The International journal of educational and psychological assessment* 2012;10(1):51.

34. Brites C, Salgado-Azoni C, Ferreira T, et al. Development and applications of the SWAN rating scale for assessment of attention deficit hyperactivity disorder: a literature review. *Brazilian Journal of Medical and Biological Research* 2015;48(11):965-72.

35. Gresham F, Elliott SN. Social skills improvement system (SSIS) rating scales: San Antonio, TX: Pearson Education Inc 2007.

36. Allen K, Marlow R, Edwards V, et al. 'How I Feel About My School': The construction and validation of a measure of wellbeing at school for primary school children. *Clinical child psychology and psychiatry* 2018;23(1):25-41.

37. Tennant R, Hiller L, Fishwick R, et al. The Warwick-Edinburgh mental well-being scale (WEMWBS): development and UK validation. *Health and Quality of life Outcomes* 2007;5(1):1-13.

38. Tschannen-Moran M, Hoy AW. Teacher efficacy: Capturing an elusive construct. *Teaching and teacher education* 2001;17(7):783-805.

For peer review only

39. Langballe EM, Falkum E, Innstrand ST, et al. The factorial validity of the Maslach Burnout Inventory–General Survey in representative samples of eight different occupational groups. *Journal of Career Assessment* 2006;14(3):370-84.

40. Maslach C, Jackson S. Leiter MP Maslach burnout inventory manual. *Palo Alto: California Consulting Psychological Press Inc* 1996

41. Harter S. Self-perception profile for adolescents: Manual and questionnaires. *Denver, CO: Univeristy of Denver, Department of Psychology* 2012

42. Stevens K. Valuation of the child health utility 9D index. *Pharmacoeconomics* 2012;30(8):729-47.

43. Chisholm D, Knapp MRJ, Knudsen HC, et al. Client socio-demographic and service receipt inventory–European version: development of an instrument for international research: EPSILON Study 5. *The British Journal of Psychiatry* 2000;177(S39):s28-s33.

44. Abikoff H, Gittelman-Klein R, Klein DF. Validation of a classroom observation code for hyperactive children. *Journal of Consulting and Clinical Psychology* 1977;45(5):772.

45. Martin P, Daley D, Hutchings J, et al. The teacher-pupil observation tool (T-POT) development and testing of a New classroom observation measure. *School Psychology International* 2010;31(3):229-49.

46. Gale NK, Heath G, Cameron E, et al. Using the framework method for the analysis of qualitative data in multi-disciplinary health research. *BMC medical research methodology* 2013;13(1):1-8.

47. Pfiffner LJ, Rooney M, Haack L, et al. A randomized controlled trial of a school-implemented school–home intervention for attention-deficit/hyperactivity disorder symptoms and impairment. *Journal of the American Academy of Child & Adolescent Psychiatry* 2016;55(9):762-70.

48. Ford T, Hayes R, Byford S, et al. The effectiveness and cost-effectiveness of the Incredible Years® Teacher Classroom Management programme in primary school children: results of the STARS cluster randomised controlled trial. *Psychological medicine* 2019;49(5):828-42.

For peer review only

For peer review only - http://bmjopen.bmj.com/site/about/guidelines.xhtml

Figure legends

Figure 1. Case series design outline

Key- grey, baseline; green, intervention; blue, follow-up

Figure 2. Intervention Draft Logic Model

For peer review only

For peer review only - http://bmjopen.bmj.com/site/about/guidelines.xhtml

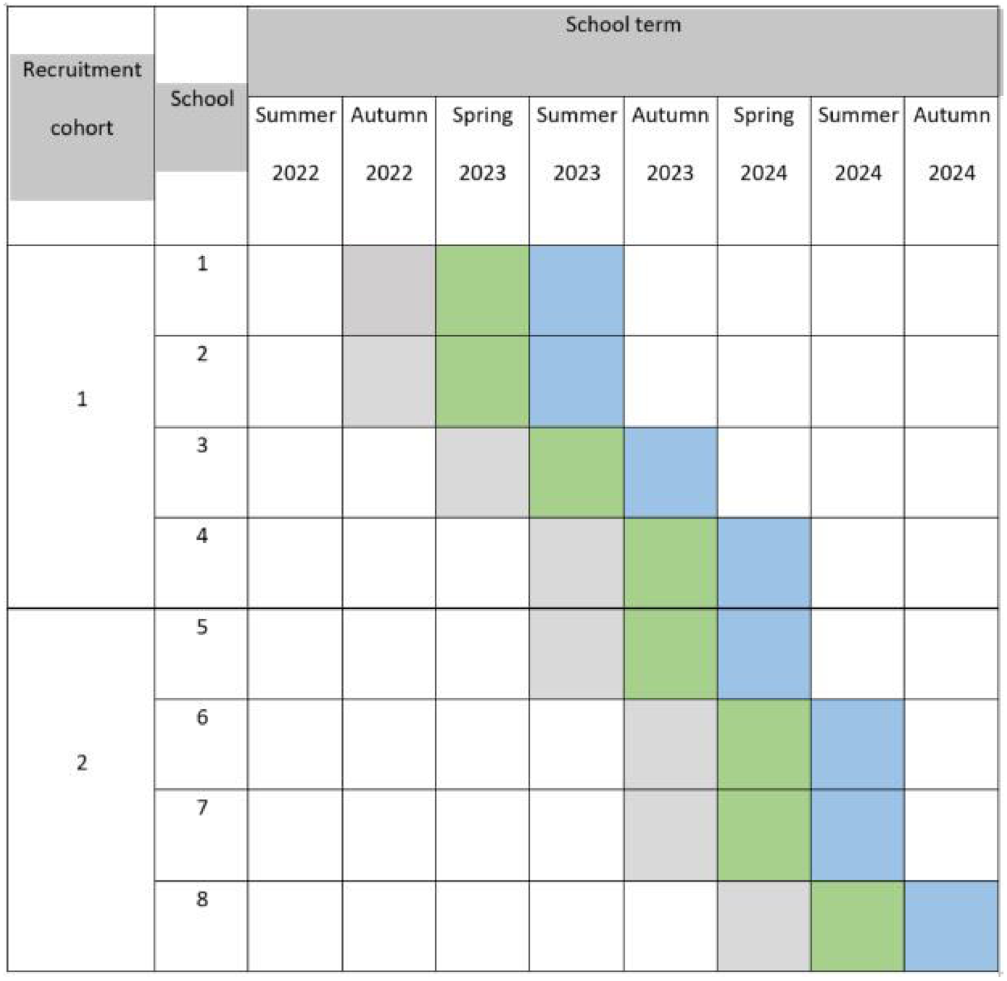

Figure 1. Case series design outline
Key- grey, baseline; green, intervention; blue, follow-up

52x51mm (300 x 300 DPI)

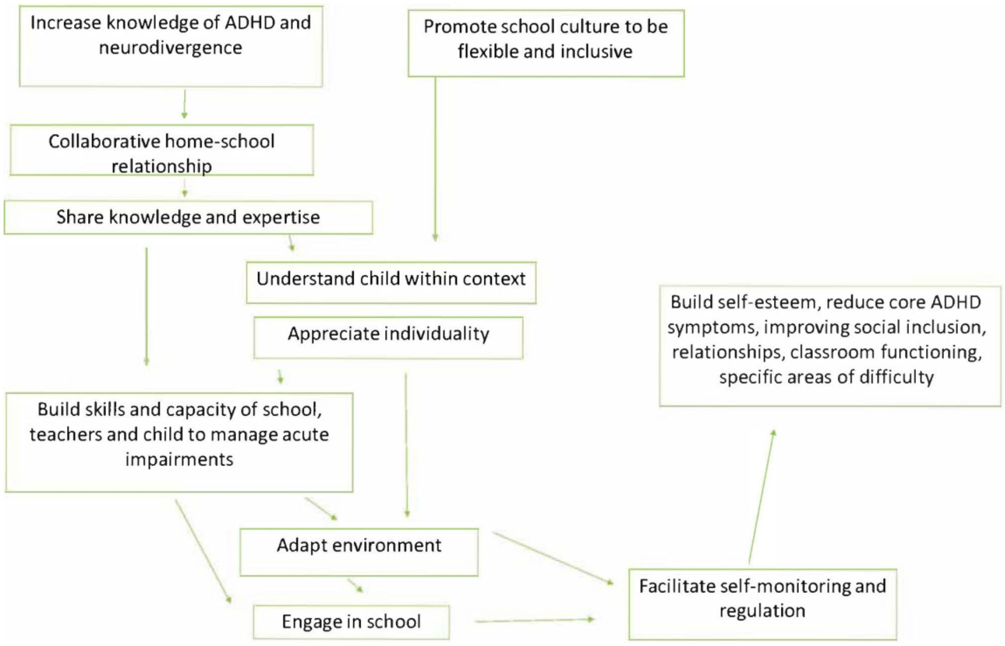

Figure 2. Intervention Draft Logic Model

169x109mm (300 x 300 DPI)

Protocol for an iterative case-series study to investigate the feasibility and acceptability of a school-based intervention for children with traits of ADHD: Supplementary Material

Full intervention description

The Tools for Schools *FLEX* toolkit will comprise a digital resource of training and resource packages and behavioural strategies organised within 'modules' that cover different classroom-based problems common to ADHD. See the main article for information on the key goals of the toolkit. Supplementary Figure 1 illustrates the participant structure and roles within a school for delivery of the toolkit.

Supplementary Figure 1. Structure of individuals within schools and participant roles in case series study

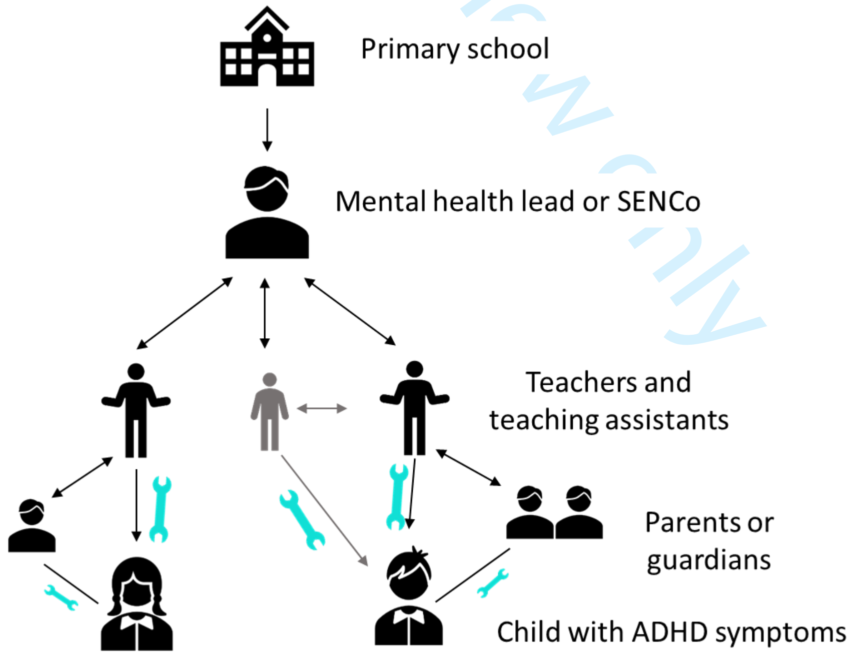

Delivery of the intervention comprises a series of steps, as indicated in the toolkit outline in Supplementary Figure 2, and described below.

Supplementary Figure 2. Outline of the *FLEX* Toolkit

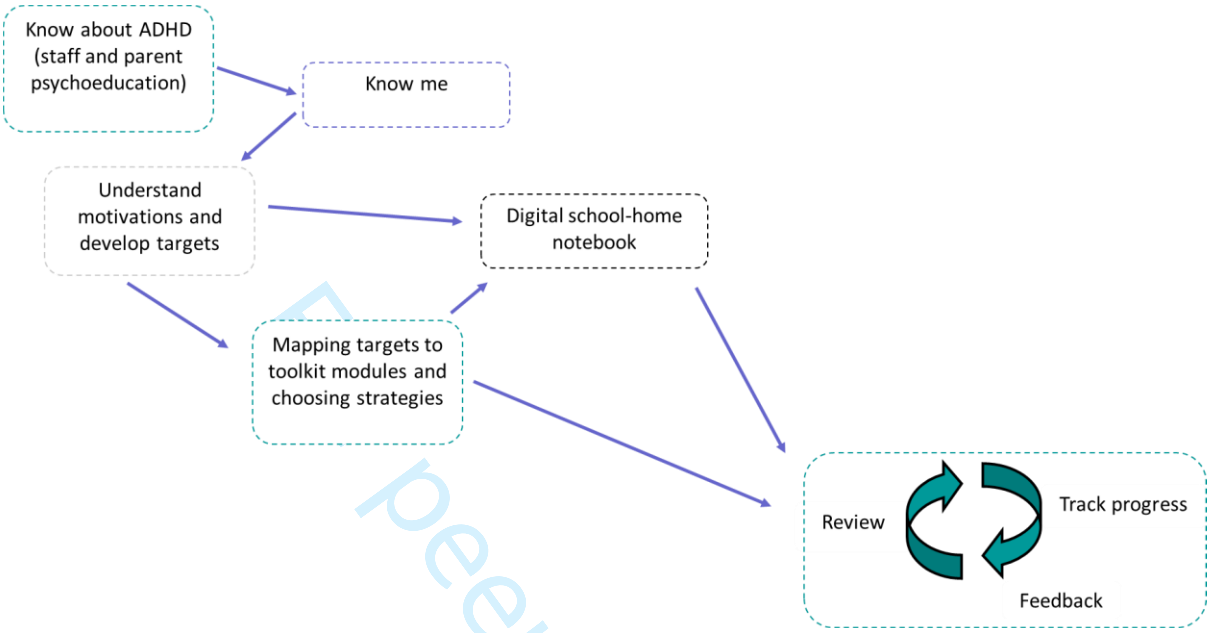

Step 1: Know ADHD

Purpose: Tailored knowledge of ADHD and relevant theory, psychoeducation. Putting staff and parents on equal footing, helping to address power dynamics between school and home and recognise expertise of each party

The toolkit will begin with online psychoeducation training for school staff and parents. This will cover ADHD as well as training on implementing the toolkit itself. Videos from people with ADHD, parents and school staff about their experiences will be included.

Key things the Know ADHD training will cover:

- ADHD is not an issue of morality or simply a deficit in attention
- Involvement of dopamine in the developing brain
- Executive functions and ADHD
- Delay aversion and ADHD
- Other theories about how ADHD traits come about

- Gender differences
- Sensory processing
- The variation between children with ADHD: every child with ADHD is different and unique, and their traits will affect them in different ways
- Growing up with traits of ADHD

Schools will also be asked to plan and deliver activities to promote inclusivity and the concepts of embracing individual differences and diversity at the outset of the study, through a participatory action research approach. Senior leadership and the SENCo will be asked to reflect on their school culture and inclusivity of school policies, make changes to these with the aim of improving flexibility around children with ADHD and their individual needs, and reflect on and evaluate these changes [23]. This might involve participating in national events such as Neurodiversity Celebration Week, having a school assembly or applying for the ADHD Foundation "ADHD friendly schools award" for example. Each school (senior leadership team [SLT] and SENCo) will decide on whether they want to implement any whole-school measures, and what form these will take. They will be supported by the research team but this is not an essential component of the toolkit, rather an exploratory component that will need to be led by each school themselves to be implementable or useful in future versions of the toolkit. The toolkit provides step-by-step instructions for the SLT to conduct an "Inclusion challenge" and makes suggestions as to what systems and processes within the school they may consider changing as part of this.

We will use the Participatory Action Research cycle of Plan, Act, Evaluate, Reflect to structure this. We ask the SLT to consider how their school could be more inclusive for neurodivergent children (including asking for input from children and others in the school) and plan how they could change this. Then they would implement the new ideas (act) for a given period of time; evaluate the changes by seeking staff and student views, and reflect on whether they were successful before implementing further changes. No data will be collected at awareness events or other activities the

For peer review only

SLT decide to enact in this step, unless they collect it for their own evaluative purposes. Staff will be asked to feedback about this during qualitative data collection instances. We anticipate researching the mechanisms of this potential component in further depth in separate research if participants report using this, implementing it and finding this component potentially useful.

Given the aim of this study is to assess feasibility and acceptability of implementation in a real-world context, we have made the decision not to make any component of the flex toolkit mandatory (although we strongly suggest that staff complete the ADHD training and the SLT do the inclusion challenge); the current UK school context is facing severe pressures and resource constraints, and we anticipate that uptake of components will be better and more representative of naturalistic use of the toolkit if they are not mandated. Whether or not to mandate this component in a future trial will be considered based on the findings of this study.

An additional consideration is how to present this information to parents whose child has traits of ADHD but is not diagnosed. The Step 1 learning is titled “know traits of ADHD” and uses minimal language about ADHD as a disorder, talking more about traits of poor attention or concentration, impulsive behaviour or acting without thinking, and hyperactivity. In the learning materials we include a segment on how this relates to the label or diagnosable disorder of ADHD. We are interested to obtain feedback from parents about this.

All of the parents in the study have had contact with their child’s SENCo about the child’s difficulties or perceived need for additional support in school prior to the study. When selecting and approaching parents, this is discussed expressly with the SENCo and they have a discussion with parents prior to the research team becoming involved. The study information sheet also talks about traits of ADHD, and parents are therefore aware of the nature of the toolkit before they get to Step 1.

For peer review only

Step 2: Know me (the child)

Purpose: Children with ADHD are more able to engage with something if it interests them. They often feel misunderstood by others and this activity is intended to allow staff to understand and empathise with the child, seeing things from their perspective. Children may not be able to verbalise their emotions and feelings and understanding how they behave when happy, sad, angry or excited is an important component of functional behaviour analysis and behaviour change approaches. This activity aims to draw these out for each individual.

Children will complete a strengths-focussed activity with the teacher or TA to identify strengths or hobbies that could be used to personalise the intervention and to help school staff understand the child from their perspective. This activity will be bespoke depending on what the child wants and likes to do and could be delivered to a whole class, but might include making a film or animation, creating a story or photo board, or building a virtual version of oneself on a computer game.

Step 3: Understand motivations and develop targets

Purpose of step: Sharing knowledge and expertise between school and home, focussing on understanding antecedents and consequences of behaviour in order to understand how best to modify. Mutually deciding on which targets are most important (and the relationships between potential targets)

At the end of the baseline term and the beginning of the intervention term, SENCos, teachers and other relevant staff will work alongside the child and family to complete a functional behaviour analysis of two problem areas where the child is currently struggling. We have chosen to constrain this to two areas in order to provide focus during the use of the toolkit in one school term for research purposes, and to support those testing the toolkit to identify the key areas for improvement and therefore observable change. Understanding the antecedents and consequences of behaviour will be crucial to identify where to intervene and to understand which modules and strategies from the toolkit are likely to be useful. The meetings will result in identifying two core

For peer review only

behaviours that will be transformed into the intervention “toolkit targets”. The core problems will be operationalised into tangible and achievable goals (e.g. stay in seat for 5 minutes, with the number of minutes increasing as the child meets the target; having more than one friend to play with at lunchtime, starting with one day per week and increasing over time; having three adults at school they can go to if they have a problem, starting with one adult and knowing what and how to communicate to them if there is a problem) over the course of two meetings.

The form of these meetings may vary depending on parent engagement and preferences. One option that will be piloted is inviting families and children to join these meetings via videoconference. This would allow the child to feel involved in the process if they wish to engage, but also provides them with the option to withdraw from the conversation, and parents will make the decision as to whether they wish for their child to be involved in these meetings. Through discussions with the planning group, parents identified that they wish to be consulted and to bring their perspective to these meetings, however they may also find such meetings are difficult emotionally, as the focus will be on their child’s difficulties. As such, parents may feel more empowered or comfortable joining from their home environment. Alternately, parents and children may wish to attend these meetings in person at school. Parents will make the decision as to where/through what medium the meeting takes place.

The purpose of this step is to encourage school staff and parents to become aware of the layers of context around the child, highlighting potential points for intervention that do not rely on intrinsic changes to the child themselves. This step of the intervention focusses on what achievements it is hoped the child will make, however the strategies that will be implemented to achieve these are primarily designed to adapt the environment around the child, for example scaffolding them with classroom tools and activities to improve organisation through making it clearer to the child where their possessions can be found or visual aids to demonstrate what will be needed for the next activity; introduction of whole-class games on identifying and providing positive feedback to peers

For peer review only

where all classmates are reinforced for “picking up the positives” to improve self-esteem; or incorporation of movement-based learning strategies throughout the school day to improve ability to concentrate, and the teacher changing the format of the work that the child is asked to complete.

Step 4: Digital school-home notebook

Purpose: Establishing and maintaining positive communication between teachers, TAs and parents. Research shows that conflictual and negative relationships between school and home are common for children with ADHD. Breaking negative communication cycles and establishing positive communication will improve sharing of information and ideas between home and school, and better support the child across contexts.

A communication platform for regular contact between staff and parents will be established once the child’s toolkit targets have been agreed. This was initially intended to take the form of a digital daily report card (dDRC) with staff updating home daily on the child’s progress with their targets, and parents reinforcing this at home. The purpose of this component is to develop habits of positive communication about progress and child strengths, rather than to function as a “behaviour log” or to report incidents during school.

The frequency and nature of communication using the notebook will be flexible to suit the needs of the individual child and participants surrounding them; the DRC format will be proposed as the suggested model as this has the most existing evidence for effectiveness [24 25]. However, other forms of successful communication may already be in place between school and home, or daily communication may be considered burdensome and not beneficial to both parents and teachers for a wide variety of reasons. As such, staff and parents will be asked to mutually agree on frequency of communication using the digital school-home notebook, and this will be reviewed and modified as required during the intervention period.

Step 5. Mapping targets to toolkit modules and choosing strategies

Purpose: identifying strategies within modules that are most likely to be effective based on functional behaviour analysis and understanding of the child

The SENCo and teacher will link the child's targets to the most pertinent modules and outcomes in the toolkit using a graphical map (currently called the behaviour web). This may be initially discussed in the two meetings to understand behaviour and develop targets, or may be decided following these meetings. The functional behaviour analysis will be drawn upon with a focus on the current consequences relating to the target behaviour, and alternate consequences that could encourage more adaptive behaviour.

The toolkit modules are based on individual ADHD-related outcomes selected by consensus in a Delphi study. They are:

1. Organising, planning and reminding (describe to differentiate from organising)->means self-monitoring and self-regulating
2. Paying attention
3. Feeling good (self-esteem, self-worth)
4. Belonging and relating (relationships)
5. Pausing for thought (inhibiting impulsive behaviour)
6. Channelling energy (hyperactivity)

Modules will contain a bank of approximately 10 non-pharmacological strategies that can be used at individual or classroom level to target the outcome, organised into domains such as "Behaviour management" "Training tools" and "Changing the classroom" through the co-creation process. The planning group have indicated that the focus of the toolkit should be on inclusivity: supporting children to remain in the classroom and allowing the child to access the same education and opportunities as their classmates. Strategies will be developed with this goal in mind. In addition, modules will contain resources on maintaining strategies once implemented.

For peer review only

Step 6. Implementing and reflecting on strategies

Purpose: produce improvement in target behaviours that can be maintained

Once teachers and SENCos have determined the modules to implement through consideration of the functional behaviour analysis and the child's agreed targets, the SENCo will support the teacher and TAs to select and implement strategies. Strategies have been designed to be primarily group-based games or activities, or reminders and support for teachers to incorporate new techniques to adapt around the child, with the aim that this will lead to improved outcomes for the child. None of the strategies require the child to be removed from the classroom, and although some could be used 1:1 with the child, they are primarily presented as group-level activities to promote inclusion. A significant number of strategies focus on "belonging and relating", specifically aiming to promote peer inclusion, celebrate individual differences, and destigmatise neurodevelopmental (and other) differences.

We anticipate that one module will be implemented in each half-term of the intervention term, however there will not be a restriction on this, nor how many strategies within a module can be attempted; we anticipate that some strategies will have already been tried by a school or teacher, or it may be apparent that other strategies do not suit the child and so different strategies may be selected. Repeated measures of ADHD symptoms, classroom functioning, and measures specific to the outcomes relevant for each module will be collected from teachers, TAs and children as appropriate (Table 3 shows the data collection schedule relative to the stage within the study).

Co-production of the toolkit with the planning group

Parents, children with ADHD, adults with ADHD, SENCos, educational psychologists and teachers (the planning group) were initially informally interviewed about their experiences, and what they perceive works for them. These findings were narratively synthesised and written up in one document.

For peer review only

They contributed to an exhaustive list of the kinds of behaviours that may form targets for the intervention, and the modules that these would fall within (e.g. being ‘easily led by others’ mapping to the ‘pausing for thought’ module); with some behaviours mapping to multiple modules depending on the underlying reason (e.g. ‘risky or dangerous behaviour’ mapping to either ‘paying attention and engaging’ or ‘pausing for thought’ depending on whether the primary reason is impulsivity or inattentiveness).

The intervention logic model of change was reviewed and refined with the planning group, who then had input into the performance objectives (the smallest steps of behaviour change required to meet each behavioural goal for each person e.g. teacher, child, senior leadership) through reviewing the lists in an ‘activity workbook’ format, adding thoughts and attending discussion meetings.

These performance objectives were also categorised by the determinants of behaviour (whether the change in behaviour required aligned with skills, values, beliefs, attitudes, knowledge or experience). Initial strategy ideas were then drafted by AR to match performance objectives with input from behaviour change taxonomies to select relevant delivery methods.

These draft strategy ideas were then reviewed by the planning group, and changed entirely or improved based on their input. Strategy instructions were then written by AR, reviewed by the planning group once more and refined and the prototype toolkit was produced. PDF resources to support teachers in delivering each strategy were generated by the research team, with the planning group suggesting resource ideas and providing further input and feedback at the ‘alpha testing’ stage of the prototype. Resources and strategies were then refined and finalised, prior to the beta version (to be used in the feasibility study) being rolled out.

For peer review only

Example questions for Toolkit iteration feedback interviews

1. *Could you tell me about how you have used the toolkit?*
2. *What have your experiences been of using the toolkit? Let's talk about it step-by-step so you can give me your reflections on each component. First, overall did you think the toolkit was useable in Primary school? Why/why not? And do you think other schools and families would find it acceptable? Why/why not? Do you think other schools and families would be able to implement it? Why/why not?*
3. *What did you like most about the toolkit? What did you like least? Could you tell me some more about that?*
4. *How was the Know ADHD component? Did you use it, what was good about it and what was difficult or challenging? What would help you to use it better in the future? Was there anything missing that you think is important for people to know about ADHD?*
5. *How was the Know Me component? What did you do with the child to complete this activity? What format did the child choose (e.g. video, storybook)? How did you feel about it, and how do you think the child felt about doing the activity? Did you find the product of this Know Me step was useful for the rest of the toolkit? Could you tell me why you think that? Would you make any changes to the Know Me step or instructions, and what would they be?*
6. *Who set up the meetings with school staff to assess and understand the two main problems? How did those meetings go and who was there? When did you have the meetings? What was good and bad about the meetings? Did you manage to define two target goals for the child? Were parents and the child involved (if so how)? Would you do anything differently if you did it again? Should the toolkit have different instructions? How did you understand the antecedents, behaviours and consequences of each behaviour? Who helped to figure those out, how and how long did it take? Was that too much time for you?*

For peer review only

Table of quantitative measures

Outcome	Measure
ADHD symptoms	Strengths and Weaknesses of ADHD and Normal Behavior Scale (SWAN)
Classroom functioning	Problem behaviour subscale of the Social Skills Improvement System (SISS)
Dimensional psychopathology	Strengths and Difficulties Questionniare
School functioning	Academic and social skills subscales of the SISS
Child-reported satisfaction with school	How I Feel About My School questionnaire
Teacher wellbeing	Warwick-Edinburgh mental wellbeing scale 14-item Teacher survey; the Relationship with Work survey from the Maslach Burnout Inventory-General Survey; the Teacher Sense of Efficacy Scale;
Health and education resource use	Bespoke questionnaire available from the authors on request
Child quality of life	Child and parent report Child Health Utility-9D
Additional module measures (where not captured by the above)	
Child self-esteem	Harter Self-Perception Profile for Children
Belonging and relating	PROMIS Pediatric Peer Relationships - Short Form 8
Organising, planning and reminding	Children’s Organizational Skills Scale

Risks and mitigation plans

Risk 1. Schools are open but due to periods of Covid-related closure, child development and socialisation has been impacted.

Mitigation: as part of the toolkit development process we are liaising closely with primary schools, parents and teachers to monitor ongoing changes to school environments and cultures. We anticipate that, if anything, there will be greater demand for the toolkit if more pupils are struggling with relevant interpersonal and developmental challenges. There may be the need to adapt the focus of modules, for example, to support school attendance explicitly. Potential adaptations will be discussed with the academic advisory team and planning group before implementation.

For peer review only

Risk 2. Schools are closed due to ongoing Covid-19

Mitigation. The planning group has advised that many of the children meeting eligibility criteria for the study have attended school through prior lockdowns and closure periods due to their vulnerable status and additional needs. We will explore whether sufficient children and staff are attending school at the time of recruitment and data collection, and if so will continue as planned with implementation of the intervention. Data collection will be completed remotely through digital questionnaires and online video conferencing software meeting data protection requirements if external (i.e. research) staff are unable to access schools due to covid-19 lockdowns.

Risk 3. Unanticipated adverse effects of intervention

Mitigation: adverse effects will be monitored through qualitative data collection during the use of the intervention, and participating school staff will follow a defined procedure for reporting of negative impacts as soon as possible after they come to light. Should a child or other participant of the study report severe worsening of ADHD symptoms or distress, the PI will consult with clinically-trained members of the academic advisory board to decide on the most appropriate course of action. This may include: the clinical academic speaking directly to staff and families to ascertain whether the adverse effects are caused by the intervention or research study or are external to this, the child and family withdrawing from the study, or bespoke plans being put in place to monitor and manage the situation. Should a child or family withdraw from the study due to this, their existing data will not be used unless express consent is given at the time of withdrawal, and they will be able to continue to access the prototype intervention (as long as the study team are confident that this is not the cause of adverse effects) if they wish.

For peer review only

Limitations

This study is a feasibility study, and due to the iterative case-series design not all participants will get the same version of the study prototype. Therefore we will not be able to quantitatively measure or compare changes in our outcome measures across all study participants, instead we are restricted to examining whether individual trends of change are replicated across individuals.

In addition, due to the modular nature of the toolkit, the sample size for a future cluster randomised controlled trial may have to be large in order to obtain sufficient numbers of participants using each module.

For peer review only

Model consent form

Tools for Schools: a prototype toolkit for ADHD interventions to be used in primary school teaching.
Consent form for parents or guardians on behalf of children –Version 3: January 2022

I have read the provided information sheet, Version 4, concerning Tools for Schools project and I understand what it is about.
I fully understand what is expected of me and all my questions have been answered to my satisfaction.
I understand I am free to request further information at any stage.

As a **parent or guardian on behalf of my child,** I am aware that:

(please circle Yes or No)

1. My child's participation is entirely voluntary. Yes / No

2. I am able to withdraw my child's participation from the project without consequence or disadvantage. Yes / No

3. I understand that relevant sections of the data collected during the study may be looked at by members of the research team, and individuals from the University of Exeter where it is relevant to my taking part in this research. I give my permission for these individuals to have access to my records. Yes / No

4. The researchers do not anticipate any of the participants to experience any discomfort or harm during the project. Yes / No

5. I can contact the researchers with any questions, queries or worries at any point during the study (using the contact details provided in the information sheet). Yes / No

6. My child's data will be anonymous in any published works relating to the project. Yes / No

7. In the future, my child's anonymous data may be used in other related research. Yes / No

8. Recordings can be taken of interviews and class groups my child takes part in. Yes / No

9. My child will take part in lessons that may ask them to do things differently from other classmates. Wherever possible, this will be done in the most subtle way so as not to draw

attention to my child, but I understand this may not always be possible. Yes / No

10. My child will be asked to complete questions that other children will not do. Yes / No

11. My child might be asked to leave the classroom to do different learning/lessons, for example daily report cards. Yes / No

12. My child might take part in an ‘active interview’ (such as playing together or doing an activity) at home or another suitable location. I may be asked to join. Yes / No

13. Records (e.g. photographs) may be taken of my child’s work. Yes / No

14. In the future, researchers may contact me again to follow up on my child’s progress. Yes / No

By signing below, I agree/consent to take part in this research project.

…………………………………..	…………………………………..	………………
(Printed name of child)	(signature of child)	(Date)
…………………………………..	…………………………………..	………………
(Printed name of parent/guardian)	(signature of parent/guardian)	(Date)
…………………………………..	…………………………………..	………………
(Printed name of researcher)	(signature of researcher)	(Date)

Model information sheet

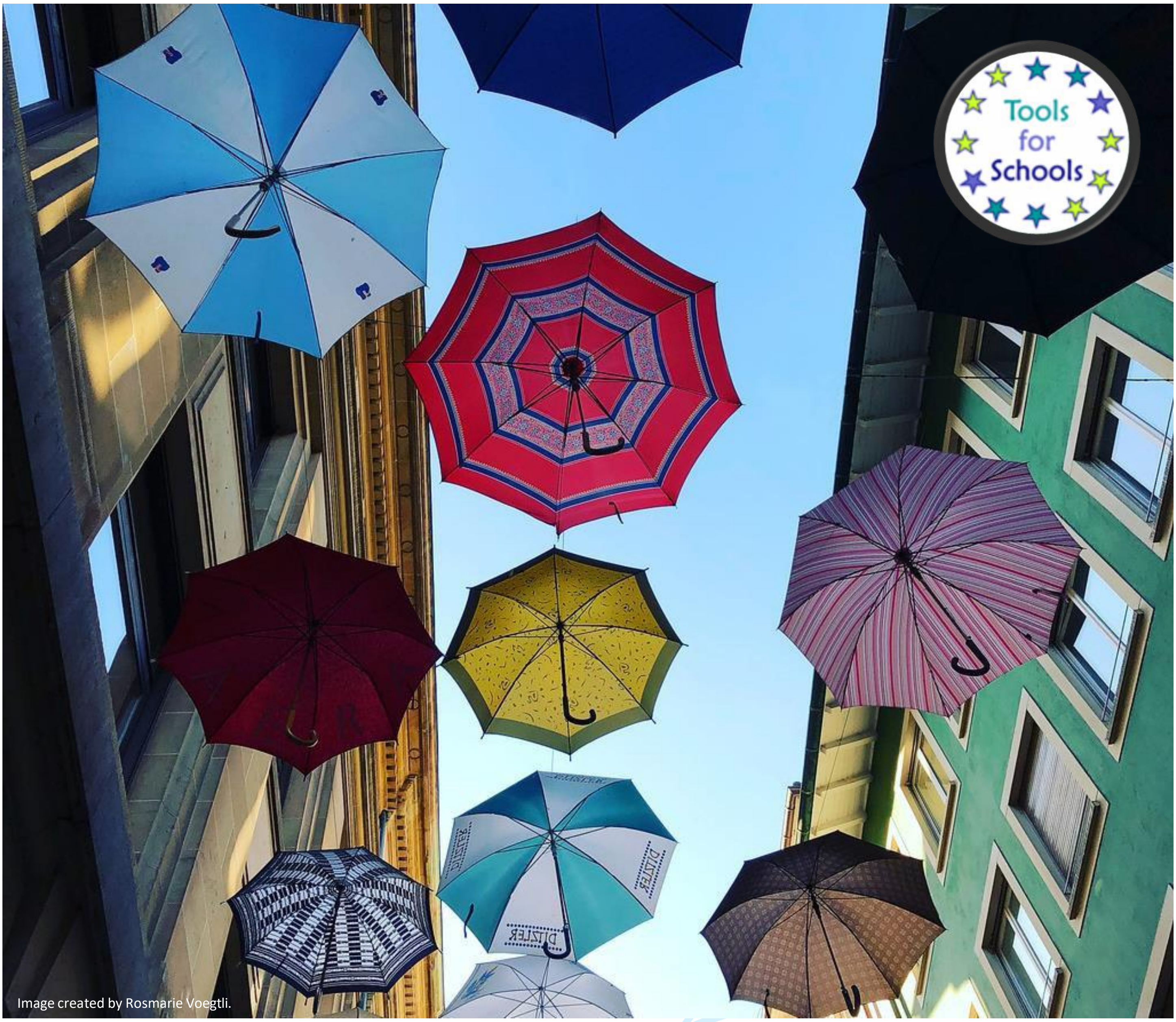

Image created by Rosmarie Voegtli.

Tools for Schools: Development and early testing of a school-based toolkit to support children in school

Information Sheet for Parents, Guardians or Carers: Version 4 Jan 2022

Contents

Glossary 2
Background and aims of this research study 3
Overview of the Tools for School toolkit 4
Your role in the research 5
What does your child need to know? 6
Potential Unintended Consequences 7
Withdrawing your consent 8
Confidentiality and data protection 8
Funding 9
Ethical approval 9
Contact details 10
Complaints 10

For peer review only

http://blogs.exeter.ac.uk/toolsforschools/

Glossary

ADHD = Attention Deficit/Hyperactivity Disorder

SEN(D)Co = Special Educational Needs (and Disabilities) Coordinator

TA = Teaching Assistant (also known as Learning Support Assistant, LSA)

For peer review only - http://bmjopen.bmj.com/site/about/guidelines.xhtml

Thank you for showing an interest in this research. It focuses on early testing of a school-based toolkit of behavioural strategies and adjustments. These will improve the school experience of students who are hyperactive, impulsive or have problems with attention, causing difficulties for the child at school.

You have received this information sheet after your child's school have approached you because they think your child might benefit from taking part in this research. Please take time to carefully read this leaflet so that you have the information you need or listen to the audio/video version here. You can also request a meeting with the research team or your school's SENCo to discuss what will happen in the study and if you would like further clarification on the overview of the toolkit and study protocols.

Do ask the research team any questions you have before signing the consent form for you or your child. It is important that you are comfortable for both you and your child to take part in this study. Any other parents, guardians or main carers of the child who might like to take part would also be welcome.

If you decide to participate, we thank you. If you choose not to, there is no disadvantage to you, and we thank you for considering our project.

Background and aims of this research study

Every classroom has at least one child (on average) who will struggle to sit still, pay attention and resist impulses to do things like jumping out of their seat. Strategies are needed to help teachers support these students, but current advice is often complicated, and no child is the same. This can lead to teachers struggling to develop the right strategies for each child.

In this project, we have worked closely alongside experts, teachers and parents to develop a prototype (early version) of a toolkit which includes a range of strategies for teachers to use to support school children. Your child's school has agreed to take part in this research already and think your child would suit this toolkit and research study, which is why we are contacting you. The toolkit will be used with your child for one school term, but we will be collecting information from you, your child and their teachers for one term before this and for one school term after the toolkit is tried.

Tools for Schools

For peer review only - http://bmjopen.bmj.com/site/about/guidelines.xhtml

08/03/2022 Version 4

The aim of this current step of the study is to see if the toolkit is acceptable and practical to school staff, children and their parents. It is important to know that we are not trying to diagnose or "treat" your child. The toolkit is at an early stage, and we are not yet sure how much it will help. But we hope that in the future this toolkit will help children to have a better primary school experience.

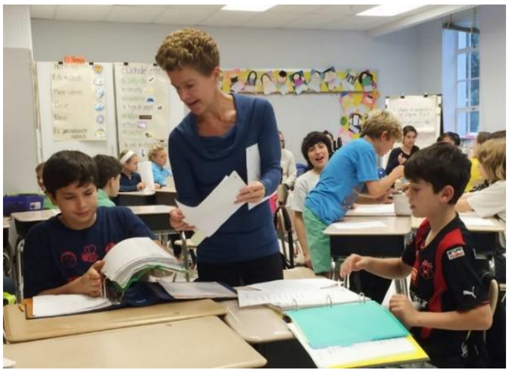
Image created by woodleywonderworks.

Overview of the Tools for School toolkit

The Tools for Schools toolkit will be mainly digital (a website), with several key steps as shown below. We want the toolkit to support your child to remain in the classroom and allow them to access the same education and opportunities as their classmates. Your child's SENCo, class teacher and teaching assistants will use the toolkit with your child in school. To keep you as parents 'in the loop' and to make the most of your knowledge of your child, you will be asked to join in for several parts of the toolkit. You can find out more information about the parts of the toolkit through the videos and descriptions on our website.

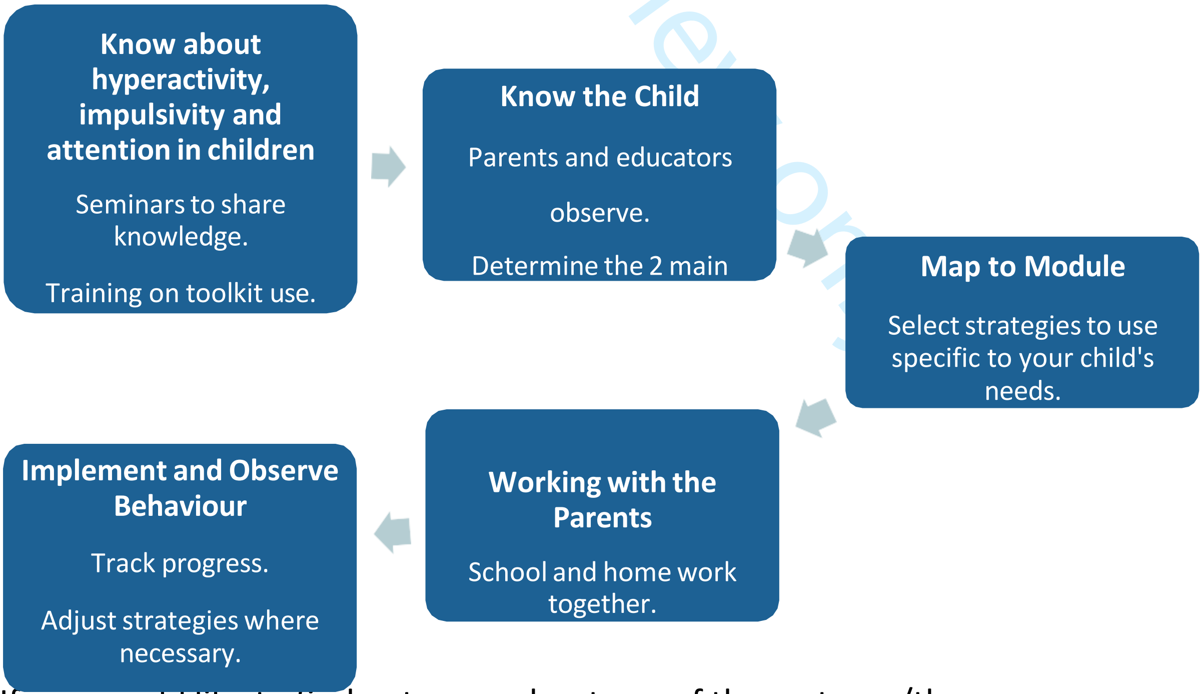

If you would like to find out more about any of these stages/the toolkit, please visit: http://blogs.exeter.ac.uk/toolsforschools/

For peer review only - http://bmjopen.bmj.com/site/about/guidelines.xhtml

Your role in the research

As a parent, guardian or carer of a child using the Tools for Schools intervention, your role will include:

- Possibly completing a brief questionnaire to see if your child is eligible to take part.
- Watching an introductory video and completing training provided by the Tools for Schools research team.
- Completing questionnaires about the healthcare, education and social services you use to support your child, and your child's wellbeing and quality of life (both before and after the toolkit is used).
- Two or three interview conversations with research staff. It is up to you where these take place (e.g. over the phone/zoom, at home or another convenient location for you). These may be recorded
- Taking part in two meetings with your child's teacher and SENDCo to help identify what the toolkit should target to best support your child (you could join these online or in person).
- Working with your child and school staff to use strategies selected in the toolkit.
- Chatting to the research team over the phone about any good and bad things you are experiencing using the toolkit so we can make changes if need be. These may be recorded.
- Using a digital home-school notebook – this will allow you and your child's teacher to share information on your child's progress and ensure strategies are reinforced at home.
- Give the research team permission to collect information about your child from you, school staff and your child.

To show our gratitude, we will provide £20 to your family (£10 for you and £10 for your child) for participating in the interviews.

For peer review only - http://bmjopen.bmj.com/site/about/guidelines.xhtml

We would also like you to give the following permissions on behalf of your child:

- Permission for your child to participate in the research and try the strategies in the toolkit with school staff support.
- Permission for one of the research team to observe your child in school to see how their behaviour changes with different strategies (this will be done subtly without making your child stand out from their classmates and will not disrupt your child's school day).
- Permission for us to ask your child to complete two short age-appropriate questionnaires; one about how they feel about their school, and one about their quality of life (twice during the research study).
- Permission to keep records/photographs of your child's work
- Permission for the research team to conduct an 'active interview' (such as playing together or doing an activity) at home or another suitable location with your child, finding out more about whether they liked or disliked the toolkit strategies. You, another parent/guardian or a sibling can join the child for this if they want you to.

For peer review only

What does your child need to know?

Your child should understand that they are participating in research but it is up to you how much you want to tell them. The toolkit is being tried as part of the usual school day, with SENCos and teachers working to support children in school as usual, so your child need not know that they are doing something new or different if you think this will make them uncomfortable.

We will need your consent for your child to participate, and their verbal agreement that they are happy to take part in a research study and to complete questionnaires. We want to ensure we are mindful of your child's "emotional thermometer" and will not be making your child do something they do not want to do. To achieve this, we will discuss with you the best way to get permission from your child to take part in certain strategies in a way that you and your child are happy with.

Tools for Schools

For peer review only - http://bmjopen.bmj.com/site/about/guidelines.xhtml

Potential Benefits

Benefits for your child include improving their relationship with their teacher and making school a more positive experience for them through:

- School staff and parents becoming better informed about how to work with children who struggle with attention, impulsiveness, or hyperactivity and what it is like for families and children living with children who have issues with this (sometimes diagnosed as ADHD).
- Improvement in teachers' awareness about attention, impulsiveness, or hyperactivity in the classroom.
- Giving teachers easy-to-use strategies to better identify and support students with their attention, impulsiveness, or hyperactivity

Benefits for you include:

- Picking up some tips from the strategies that may help you and your family at home
- Improve your communication with your child's school through the home-school notebook

We hope that access to the Tools for Schools toolkit will help your child's school experience and relationships with school staff. However, the main aim of this study is to test a prototype before it is tried with a wider range of schools and children. In the long run, we hope this will improve support (mostly at school, but also at home) for many children in education.

Potential Unintended Consequences

It is possible that the toolkit may lead to children feeling singled-out from their peers due to being treated differently. We aim to avoid this and any other negative impacts wherever possible so please talk to us about any concerns you may have. Via the toolkit children in the study will hopefully develop social skills and greater self-esteem which may help them build stronger relationships with other children. However, you and your child are free to withdraw at any time if you or they wish to.

Possible negative impacts of the toolkit will be monitored throughout the study and school staff will follow a defined procedure for reporting any negative impacts as soon as possible after they come to light. The research team will abide by any existing safeguarding protocols your child's school has as well.

For peer review only - http://bmjopen.bmj.com/site/about/guidelines.xhtml

Should a child or other participant of the study report severe worsening of symptoms or distress, you will be informed and the use of the toolkit with your child will be temporarily stopped. From there the lead researcher (Dr Abby Russell) will consult with members on the study advisory board to decide on the most appropriate course of action. This can include: a clinician speaking directly to you and the school to see whether the negative effects are caused by the toolkit or research study or are external to this; your child and family withdrawing from the study; or individual plans being put in place to monitor and manage the situation.

Withdrawing your consent

- You (and anyone else involved) may withdraw from participation in the study without needing to provide a reason at any point.
- You can choose to withdraw you or your child's participation and/or any data you and they have provided.
- Data can only be removed so long as the withdrawal is done before the data is anonymised, as at this point, we will not know which information is linked to your individual child.
- Should you withdraw from the study due to negative effects, your existing data will not be used unless you give us consent at the time that you withdraw.
- Your child will be able to continue to access the prototype intervention (as long as the study team are confident that this is not the cause of adverse effects) if they wish.

Confidentiality and data protection

The University of Exeter processes personal data for the purposes of carrying out research in the public interest. The University will endeavour to be transparent about its processing of your personal data and this information sheet should provide a clear explanation of this. If you do have any queries about the University's processing of your personal data that cannot be resolved by the research team, further information may be obtained from the University's Data Protection Officer by emailing informationgovernance@exeter.ac.uk. or at http://www.exeter.ac.uk/ig/

For peer review only - http://bmjopen.bmj.com/site/about/guidelines.xhtml

The data we will collect from you includes recordings of your interviews with the research team, notes taken by the interviewer at the time and any questionnaires that we ask you to complete. The data we will collect from your child includes a recording of their active interview, any questionnaires they complete and information from observing them at school.

Recordings will be written down word for word and anonymised before being analysed along with other interviews we are conducting. The data collected will be securely stored in such a way that only the researchers working on the project can access it. In the interview if you feel uncomfortable, you will be reminded of your right to not answer any particular question(s).

The results of this project may be published, but any data included will not be individually identifiable – so nobody will know which schools or children took part. A summary of the study findings will be sent to you. You will be provided with a copy of your interviews as well if you wish. Additionally, the anonymised data you provide may be used other related research – however this is optional.

Funding

This research is funded by the National Institute of Health Research (NIHR): the research arm of the NHS. Specifically, it is funded by an Advanced Fellowship – Stage 2, Dr Abigail Russell, NIHR300591. The funding currently covers the project for 5 years, which began in July 2020.

Ethical approval

This study has received ethical approval from the University of Exeter College of Medicine and Health Research Ethics Committee (reference Jan22/B/300).

For peer review only

For peer review only - http://bmjopen.bmj.com/site/about/guidelines.xhtml

Contact details

If you have any questions about the Tools for Schools project, either now or in the future, please feel free visit our website for further information, or to contact [removed for review] directly, who leads the research team:

[Researcher name}

Children and Young people's Mental health research collaboration (ChYMe)
University of Exeter College of Medicine and Health

[phone numbers]

ToolsForSchools@exeter.ac.uk

[researcher email and twitter

handle]

Complaints

If you have any complaints about the way in which this study has been carried out, please contact the Chair of the University of Exeter Medical School Research Ethics Committee:

cmhethics@exeter.ac.uk

For peer review only - http://bmjopen.bmj.com/site/about/guidelines.xhtml

SPIRIT

STANDARD PROTOCOL ITEMS: RECOMMENDATIONS FOR INTERVENTIONAL TRIALS

SPIRIT 2013 Checklist: Recommended items to address in a clinical trial protocol and related documents*

Section/item	Item No	Description
**Administrative information**		
Title	1	Descriptive title identifying the study design, population, interventions, and, if applicable, trial acronym: included
Trial registration	2a	Trial identifier and registry name. If not yet registered, name of intended registry : Not applicable
	2b	All items from the World Health Organization Trial Registration Data Set: Not applicable
Protocol version	3	Date and version identifier (added in Author contributions)
Funding	4	Sources and types of financial, material, and other support: included
Roles and responsibilities	5a	Names, affiliations, and roles of protocol contributors: included
	5b	Name and contact information for the trial sponsor: funder (included)
	5c	Role of study sponsor and funders, if any, in study design; collection, management, analysis, and interpretation of data; writing of the report; and the decision to submit the report for publication, including whether they will have ultimate authority over any of these activities: in funding section
	5d	Composition, roles, and responsibilities of the coordinating centre, steering committee, endpoint adjudication committee, data management team, and other individuals or groups overseeing the trial, if applicable (see Item 21a for data monitoring committee): Not applicable for feasibility study.
**Introduction**		
Background and rationale	6a	Description of research question and justification for undertaking the trial, including summary of relevant studies (published and unpublished) examining benefits and harms for each intervention: included
	6b	Explanation for choice of comparators: added (introduction)
Objectives	7	Specific objectives or hypotheses: included

For peer review only

Trial design	8	Description of trial design including type of trial (eg, parallel group, crossover, factorial, single group), allocation ratio, and framework (eg, superiority, equivalence, noninferiority, exploratory): added in intro

Methods: Participants, interventions, and outcomes

Study setting	9	Description of study settings (eg, community clinic, academic hospital) and list of countries where data will be collected. Reference to where list of study sites can be obtained: added to methods (description of settings). Site list is NA due to research confidentiality
Eligibility criteria	10	Inclusion and exclusion criteria for participants. If applicable, eligibility criteria for study centres and individuals who will perform the interventions (eg, surgeons, psychotherapists): included
Interventions	11a	Interventions for each group with sufficient detail to allow replication, including how and when they will be administered: included
	11b	Criteria for discontinuing or modifying allocated interventions for a given trial participant (eg, drug dose change in response to harms, participant request, or improving/worsening disease): added to Intervention delivery
	11c	Strategies to improve adherence to intervention protocols, and any procedures for monitoring adherence (eg, drug tablet return, laboratory tests): process evaluation
	11d	Relevant concomitant care and interventions that are permitted or prohibited during the trial: added to procedure
Outcomes	12	Primary, secondary, and other outcomes, including the specific measurement variable (eg, systolic blood pressure), analysis metric (eg, change from baseline, final value, time to event), method of aggregation (eg, median, proportion), and time point for each outcome. Explanation of the clinical relevance of chosen efficacy and harm outcomes is strongly recommended : included (analysis, quantitative measures)
Participant timeline	13	Time schedule of enrolment, interventions (including any run-ins and washouts), assessments, and visits for participants. A schematic diagram is highly recommended (see Figure): included
Sample size	14	Estimated number of participants needed to achieve study objectives and how it was determined, including clinical and statistical assumptions supporting any sample size calculations: included
Recruitment	15	Strategies for achieving adequate participant enrolment to reach target sample size: NA

Methods: Assignment of interventions (for controlled trials) NA

Allocation:

Sequence generation	16a	Method of generating the allocation sequence (eg, computer-generated random numbers), and list of any factors for stratification. To reduce predictability of a random sequence, details of any planned restriction (eg, blocking) should be provided in a separate document that is unavailable to those who enrol participants or assign interventions
Allocation concealment mechanism	16b	Mechanism of implementing the allocation sequence (eg, central telephone; sequentially numbered, opaque, sealed envelopes), describing any steps to conceal the sequence until interventions are assigned
Implementation	16c	Who will generate the allocation sequence, who will enrol participants, and who will assign participants to interventions
Blinding (masking)	17a	Who will be blinded after assignment to interventions (eg, trial participants, care providers, outcome assessors, data analysts), and how
	17b	If blinded, circumstances under which unblinding is permissible, and procedure for revealing a participant's allocated intervention during the trial

Methods: Data collection, management, and analysis

Data collection methods	18a	Plans for assessment and collection of outcome, baseline, and other trial data, including any related processes to promote data quality (eg, duplicate measurements, training of assessors) and a description of study instruments (eg, questionnaires, laboratory tests) along with their reliability and validity, if known. Reference to where data collection forms can be found, if not in the protocol: included
	18b	Plans to promote participant retention and complete follow-up, including list of any outcome data to be collected for participants who discontinue or deviate from intervention protocols: NA, measuring this as part of study aims
Data management	19	Plans for data entry, coding, security, and storage, including any related processes to promote data quality (eg, double data entry; range checks for data values). Reference to where details of data management procedures can be found, if not in the protocol: added to measures section
Statistical methods	20a	Statistical methods for analysing primary and secondary outcomes. Reference to where other details of the statistical analysis plan can be found, if not in the protocol: included
	20b	Methods for any additional analyses (eg, subgroup and adjusted analyses): included

For peer review only - http://bmjopen.bmj.com/site/about/guidelines.xhtml

	20c	Definition of analysis population relating to protocol non-adherence (eg, as randomised analysis), and any statistical methods to handle missing data (eg, multiple imputation): Not applicable
**Methods: Monitoring**		
Data monitoring	21a	Composition of data monitoring committee (DMC); summary of its role and reporting structure; statement of whether it is independent from the sponsor and competing interests; and reference to where further details about its charter can be found, if not in the protocol. Alternatively, an explanation of why a DMC is not needed: Not applicable
	21b	Description of any interim analyses and stopping guidelines, including who will have access to these interim results and make the final decision to terminate the trial: included
Harms	22	Plans for collecting, assessing, reporting, and managing solicited and spontaneously reported adverse events and other unintended effects of trial interventions or trial conduct: included
Auditing	23	Frequency and procedures for auditing trial conduct, if any, and whether the process will be independent from investigators and the sponsor: not applicable
**Ethics and dissemination**		
Research ethics approval	24	Plans for seeking research ethics committee/institutional review board (REC/IRB) approval: included
Protocol amendments	25	Plans for communicating important protocol modifications (eg, changes to eligibility criteria, outcomes, analyses) to relevant parties (eg, investigators, REC/IRBs, trial participants, trial registries, journals, regulators): included in declarations
Consent or assent	26a	Who will obtain informed consent or assent from potential trial participants or authorised surrogates, and how (see Item 32): included
	26b	Additional consent provisions for collection and use of participant data and biological specimens in ancillary studies, if applicable: included
Confidentiality	27	How personal information about potential and enrolled participants will be collected, shared, and maintained in order to protect confidentiality before, during, and after the trial: included in ethics and dissemination
Declaration of interests	28	Financial and other competing interests for principal investigators for the overall trial and each study site: included
Access to data	29	Statement of who will have access to the final trial dataset, and disclosure of contractual agreements that limit such access for investigators: included

review only

Ancillary and post-trial care	30	Provisions, if any, for ancillary and post-trial care, and for compensation to those who suffer harm from trial participation: NA
Dissemination policy	31a	Plans for investigators and sponsor to communicate trial results to participants, healthcare professionals, the public, and other relevant groups (eg, via publication, reporting in results databases, or other data sharing arrangements), including any publication restrictions: included
	31b	Authorship eligibility guidelines and any intended use of professional writers: added
	31c	Plans, if any, for granting public access to the full protocol, participant-level dataset, and statistical code: included
**Appendices**		
Informed consent materials	32	Model consent form and other related documentation given to participants and authorised surrogates: included in supplementary material
Biological specimens	33	Plans for collection, laboratory evaluation, and storage of biological specimens for genetic or molecular analysis in the current trial and for future use in ancillary studies, if applicable: not applicable

*It is strongly recommended that this checklist be read in conjunction with the SPIRIT 2013 Explanation & Elaboration for important clarification on the items. Amendments to the protocol should be tracked and dated. The SPIRIT checklist is copyrighted by the SPIRIT Group under the Creative Commons "Attribution-NonCommercial-NoDerivs 3.0 Unported" license.

For peer review only

BMJ Open

Protocol for an iterative case-series study to investigate the feasibility and acceptability of a school-based intervention for children with traits of ADHD

Journal:	*BMJ Open*
Manuscript ID	bmjopen-2022-065176.R2
Article Type:	Protocol
Date Submitted by the Author:	24-Jan-2023
Complete List of Authors:	Russell, Abigail; University of Exeter, Children and Young People's Mental Health Research Collaboration (ChYMe), College of Medicine and Health<br>Dunn, Barney ; University of Exeter, Department of Psychology<br>Hayes, Rachel; University of Exeter, Children and Young People's Mental Health Research Collaboration (ChYMe), College of Medicine and Health<br>Moore, Darren; University of Exeter, Graduate School of Education<br>Kidger, Judi; University of Bristol, Population Health Sciences, Bristol Medical School<br>Sonuga-Barke, Edmund; King's College London, Institute of Psychiatry, Psychology and Neuroscience<br>Pfiffner, Linda; University of California San Francisco, Department of Psychiatry<br>Ford, Tamsin; University of Cambridge, Department of Psychiatry
**Primary Subject Heading**:	Public health
Secondary Subject Heading:	Health services research, Mental health, Qualitative research, Research methods
Keywords:	Developmental neurology & neurodisability < PAEDIATRICS, Community child health < PAEDIATRICS, Child & adolescent psychiatry < PSYCHIATRY

SCHOLARONE™
Manuscripts

Protocol for an iterative case-series study to investigate the feasibility and acceptability of a school-based intervention for children with traits of ADHD

Abigail Emma Russell[1], Barney Dunn[2], Rachel Hayes[1], Darren Moore[3], Judi Kidger[4], Edmund Sonuga-Barke[5], Linda Pfiffner[6], and Tamsin Ford[7]

[1]Children and Young People's Mental Health Research Collaboration (ChYMe), College of Medicine and Health, University of Exeter, Exeter, UK

[2]Department of Psychology, University of Exeter, Exeter, UK

[3]Graduate School of Education, University of Exeter, Exeter, UK

[4]Population Health Sciences, Bristol Medical School, University of Bristol, Bristol, UK

[5]Institute of Psychiatry, Psychology and Neuroscience, King's College London, London, UK

[6]Department of Psychiatry, University of California San Francisco, San Francisco, USA

[7]Department of Psychiatry, University of Cambridge, Cambridge, UK

Corresponding author: Abigail E Russell, University of Exeter, College of Medicine and Health, South Cloisters, St Luke's Campus, Exeter, UK EX1 2LU. E-mail: a.e.russell@exeter.ac.uk

Word count: 6023

For peer review only

Abstract

Introduction: Attention deficit/hyperactivity disorder (ADHD) is a prevalent and impairing cluster of traits affecting 2-5% of children. These children are at risk of negative health, social and educational outcomes and often experience severe difficulties at school: effective psychosocial interventions are needed. There is mixed evidence for existing school-based interventions for ADHD, which are complex and resource-intensive, contradicting teachers' preferences for short, flexible strategies that suit a range of ADHD-related classroom-based problems. They are also poorly evaluated. In this study, a prototype intervention comprising a digital 'toolkit' of behavioural strategies will be tested and refined. We aim to refine the prototype so that its use is feasible and acceptable within school settings, and to establish whether a future definitive, appropriately powered, trial of efficacy is feasible. This novel iterative study aims to pre-emptively address implementation and evaluation challenges that have hampered previous randomised controlled trials of non-pharmacological interventions. **Methods and analysis**: A randomised iterative mixed methods case-series design will be used. Schools will be randomised to the time (school term) they implement the toolkit. Eight Primary schools and 16-32 children with impairing traits of ADHD will participate, along with school staff and parents. The toolkit will be refined after each term, or more frequently if needed. Small, theory-based and data driven changes hypothesised as relevant across school contexts will be made, as well as reactive changes addressing implementation barriers. Feasibility and acceptability will be assessed through quantitative and qualitative data collection and analyses in relation to study continuation criteria, and ADHD symptoms and classroom functioning will be tracked and visually evaluated to assess whether there are early indications of toolkit utility. **Ethics and dissemination**: Ethical approval has been obtained. Results will be presented in journal articles, conferences and through varied forms of media to reach policymakers, stakeholders and the public.

Keywords: ADHD; school-based intervention; behavioural strategies; case-series; feasibility; acceptability

For peer review only - http://bmjopen.bmj.com/site/about/guidelines.xhtml

Article Summary

Strengths and limitations of this study

- This study uses an iterative staggered design in order to refine an intervention throughout the research process.
- Measures of acceptability and feasibility of the intervention and of the ability to evaluate it in a rigorous trial setting will be reviewed against continuation criteria throughout the study.
- Multiple informants will provide relevant data, including school staff and parents, and data on both positive and adverse outcomes will be captured.
- However, the iterative nature of the study means that different participants will not receive the same intervention, therefore group-level effects cannot be delineated.
- Alternate analyses will be utilised to ascertain whether there are patterns of change in symptoms and functioning that replicate across individuals instead.

Introduction

Children and young people's mental health and neurodevelopment (including ADHD) is a government priority. In the 2017 green paper, the pivotal role of schools in supporting good mental health was acknowledged. By the end of 2023, >20% of schools across the UK will have a designated mental health lead with the aim of improving access to evidence-based treatments.[1] Cost-effective, implementable, sustainable evidence-based mental health interventions that can be delivered in schools are therefore urgently needed.

Attention deficit/hyperactivity disorder (ADHD) is a neurodevelopmental disorder affecting 2-5% of children, characterised by impairing levels of impulsivity, hyperactivity and/or inattention.[2] Multiple negative life course outcomes are associated with childhood ADHD including high rates of co-occurring mental disorders, accidents, poor educational and occupational attainment and antisocial behaviour.[3] Other neuropsychiatric disorders and unintentional injury are strongly associated with ADHD, which are the leading cause of years lost due to disability worldwide in 10-24 year olds.[4] Costs to the health, education and judicial systems, social services and economic loss are substantial; 24%

For peer review only - http://bmjopen.bmj.com/site/about/guidelines.xhtml

of the mean annual cost of £5,493 is attributable to National Health Service (NHS) resources.[5] Pharmacological treatment is available and effective for some, especially in acute management of symptoms, but is not appropriate, acceptable or tolerable for all children.[6] Response is often partial[7 8], with tolerance developing over several years.[9 10] Even with drug treatment, ADHD causes particular problems in school, resulting in the outcomes described above.[11] Although ADHD medications have been shown to be effective and safe for the treatment of ADHD in the shorter term[10], licensed ADHD medications are controlled drugs and in order to access these families must receive a clinical diagnosis of ADHD, waiting lists for which in the UK are currently backlogged. Medication is not appropriate or preferable for all children with ADHD, and in the UK is not recommended as a first-line treatment for children with traits that are not severe[12]; and non-pharmacological intervention options that can be used as a precursor, alternative or supplement to pharmacological treatments are important.

ADHD causes problems in the classroom for the child, the teacher and for other children.[13] Symptoms of ADHD (which are also traits present to varying degrees in all children) make it challenging for a child to sit still, be attentive for sustained periods of time, listen to and follow instructions, or to resist impulses to shout out. Children with ADHD traits may also wander around the classroom or school building, struggle to complete school-work and need reminders to know what they are doing. This combination of characteristics is challenging for the child themselves; as they are not consciously able to control their behaviour without learning self-monitoring and regulatory strategies and are often criticised by adults and peers for their involuntary behaviour. It can also be disruptive to the usual mainstream classroom context, and there is a poor fit between the UK mainstream Primary classroom environment and the behavioural characteristics and needs of children with impairing traits of ADHD. Development of an effective school-based intervention for ADHD that overcomes the limitations of previous interventions would be likely to improve health and social outcomes. The limitations of existing school-based non-pharmacological interventions are that they usually require delivery by trained clinicians, are complex and multi-component: targeting

For peer review only - http://bmjopen.bmj.com/site/about/guidelines.xhtml

many potential deficits in every child regardless of their individual strengths and weaknesses[14-16]. ADHD encompasses a wide range of core associated symptoms and functional issues that are seen in the classroom, and existing evidence is unclear about what works for whom, or which aspects of interventions are effective. In combination, this means existing interventions are not feasible or affordable for schools to routinely deliver, they are rarely rolled out in schools and are not followed with high fidelity. There are barriers to school staff identifying evidence-based practices and implementing these with high fidelity as well as adapting to the needs of the individual, and sustaining this over time. Reviews of existing school-based interventions for ADHD have highlighted the potential efficacy of such interventions (e.g.[17-20]), and so overcoming the limitations of existing interventions is crucial to provide an implementable, effective school-based intervention for ADHD.

We are in the process of developing an intervention from high-quality evidence, based on theory around behaviour change and ADHD and from a programme of development work including a Delphi survey of key stakeholders. We are following the Intervention Mapping approach[21 22] to co-create the intervention with key stakeholders in order to adapt the format of evidence-based strategies, targeting both symptoms and associated functional impairments, to overcome key implementation issues in schools.[23] Intervention Mapping has six steps, and the full process of the toolkit development will be detailed in a subsequent publication, however in brief these are: 1. Creating a logic model of the problem; 2. Defining programme outcomes and objectives and a logic model of change; 3. Programme design; 4. Programme production; 5. Programme implementation plan; and 6. Evaluation plan[22]. The result will be a 'toolkit' of strategies in a modular format, with some core components then a selection of optional modules focussing on a different core outcome, that is designed for Primary school staff to use when working with children with traits of ADHD: the Tools for Schools *FLEX* toolkit. The toolkit will have several modules targeting different outcomes that were shown to be of importance to key stakeholders in a Delphi survey;[24] including conflict with teachers and peers, paying attention, building self-esteem and improving planning and organisation.

For peer review only - http://bmjopen.bmj.com/site/about/guidelines.xhtml

Given the limitations of existing school-based interventions for ADHD, including limited evidence of effectiveness and difficulty evaluating them in methodologically-rigorous RCTs, it is clear that the toolkit will need to be developed in a way that ensures it is acceptable, useful and feasible for school staff. In addition to this, we must address key features that will allow successful trials of the toolkit in future powered trials so that the strength of evidence for its effectiveness is comparable to other treatments such as medication. Intervention development frameworks such as the Medical Research Council (MRC) complex intervention guidelines highlight the importance of the development and feasibility phases (rather than prematurely evaluating interventions in large randomised trials); carefully optimising protocols, working closely with the target populations, planning and testing implementation, and assessing the acceptability and feasibility of the toolkit are essential to develop an intervention that can later be tested for effectiveness. The development of the toolkit will therefore take an iterative case-series approach, trialling and modifying successive versions of the prototype in order to meet the study objectives. We have not included a control group or comparison group as the study does not aim to assess effectiveness.

In this protocol we outline plans to conduct the initial evaluation and further development of the Tools for Schools *FLEX* toolkit. As the toolkit is at an early stage of development, we plan to assess the acceptability of the toolkit, and the feasibility of implementing it within the Primary school context in the UK.

Objectives

The main aims of this feasibility study are to:

1. Refine the prototype *Tools for Schools FLEX* toolkit so that it is feasible and acceptable to implement in the school setting.
2. Establish whether a future definitive trial of efficacy is feasible.

Secondary aims are to:

For peer review only - http://bmjopen.bmj.com/site/about/guidelines.xhtml

a) Identify suitable outcome measures to assess core ADHD symptoms, child and teacher wellbeing, academic progress, and identify an appropriate primary outcome (from these) for a future definitive trial.

b) Develop and test a framework for costing the toolkit, and for assessing cost-effectiveness in a future definitive trial.

c) Assess whether observational measures of behaviour, classroom functioning and teacher-reported ADHD symptoms indicate improvement following use of the toolkit.

Methods and analysis

Design

A randomised iterative mixed methods case-series design will be used. Schools will be the units of randomisation, and will be randomised to the time (school term) when they will use the toolkit. The toolkit will be refined after each school has used it for one term, or more frequently if major issues are highlighted during a schools' use of the toolkit. Small, theory-based and data driven changes hypothesised to be relevant across the school context will be made, as well as reactive changes addressing barriers to implementation. Randomisation to using the toolkit in different school terms allows for inferences to be made as to whether the intervention is improving outcomes, with consistent improvement following the introduction of the core components enabling researchers to potentially rule out alternative explanations for behaviour change, such as improvement due to other support in place or differences in behaviour across the school year. If baseline symptoms and functioning remain on stable trajectories for children across schools, changing only when the intervention is introduced, this implies it is the intervention rather than an alternate factor stimulating the change. It is a commonly used design for single-subject analyses where the intervention cannot be withdrawn and re-implemented such as in an ABAB design.[25-27]

Eight schools will participate in total in two recruitment cohorts of four (in order to avoid later participating schools having to sign up to the study years in advance); each cohort will enter the

For peer review only - http://bmjopen.bmj.com/site/about/guidelines.xhtml

study at a different time. Schools in each cohort will then be randomly assigned their individual baseline term in the study using a computerised random sequence generator (see Figure 1). The length of baseline period will be the same for each school (one school term), with schools within each cohort entering their baseline period in different school terms; the term in which the intervention is delivered is therefore staggered across the study (Figure 1). This design will allow for refining and adapting the toolkit and its implementation based on ongoing participant feedback, maximising the chances it has of being acceptable and feasible (and therefore suitable for further evaluation in a pilot and then definitive cluster randomised controlled trial) by the end of the study.

<INSERT FIGURE 1 HERE>

Patient and public involvement

A planning group of public and patient representatives, including people with ADHD (adults [n=5] and children [n=9]), parents of children with ADHD [n=12], school staff [n=9], education and health psychologists [n=4] has been established to co-create the intervention. Input from this planning group has already extensively informed the design of the intervention and study, refining research questions, selecting outcome measures and considering participant burden. The group will also shape study dissemination plans. The planning group contains around 25 people, and will be consulted throughout the course of the study to make decisions about necessary modifications to the intervention, delivery or research design of a future evaluation. In relation to the study objectives, co-developing the intervention with key stakeholders in the planning group increases the chances of the toolkit being feasible and acceptable in the Primary school context. The group will actively collaborate to refine the toolkit in response to participant feedback to achieve the first aim. They will be consulted as to whether the feasibility study results indicate that a future trial is feasible, in relation to understanding the core features of a high-quality trial. The role of the planning group in co-production of the toolkit is outlined in further detail in the supplementary material.

For peer review only - http://bmjopen.bmj.com/site/about/guidelines.xhtml

Regarding the secondary objectives, the planning group will use data collected to agree on the final primary outcome for a future definitive trial, as well as judging whether trialled measures are suitable measures to assess ADHD symptoms, academic attainment, child and teacher wellbeing. The planning group will contribute to the development of the costing framework and evaluate findings from study participants. Finally, the planning group will discuss the findings from observational measures in relation to the toolkit use, helping to understand why any emerging patterns in the data are seen.

Recruitment and Participants

Eight Primary schools, associated school staff and up to 32 children with impairing traits of ADHD (minimum 16 children) will participate in the study, and use the toolkit. All schools and all child participants will receive the intervention. Baseline data regarding treatment as usual will be collected in order to assess normal fluctuations in symptoms and response to any school-implemented treatment as usual during the baseline phase. Baseline data will not be used to exclude participants who have unstable profiles, rather to greater understand the nature of symptom and impairment fluctuation over time.

Recruitment will be through opportunistic sampling, utilising existing contacts from the planning group such as local Educational Psychology teams, and other education networks in the South West. At the time of writing, four schools have consented to participate and, given the demand for support managing children with ADHD in school, we anticipate no problems recruiting a total of eight schools. This sample of eight mainstream primary schools in the South West of England will allow sufficient variety to purposively sample schools to include a minimum of four in areas of high socioeconomic disadvantage, based on the selection of schools with an index of multiple deprivation above the regional mean. The intervention needs to be able to respond to the range of children with traits of ADHD, including those who are often unrecognised or less likely to access other support, such as girls,[28] therefore eligible schools will have at least one female pupil meeting eligibility criteria

For peer review only - http://bmjopen.bmj.com/site/about/guidelines.xhtml

and we will aim to recruit a diverse sample of children with differing ethnicities. A sample of eight schools also allows for the intervention periods to be staggered across the study and successive iterations of the toolkit. All participating schools will use the toolkit in the study for one school term.

Study participants include the school's senior leadership or head teacher, who will consent to schools' participation; the school special educational needs (and disabilities) co-ordinator (SENCo), teachers and teaching assistants (TAs) working with eligible children, eligible children with impairing traits of ADHD, and their parents. Consent will be obtained by a member of the research team through meeting with potential participants face-to-face or online.

School and school staff consent

Each school's senior leadership team will consent to school's participation. They will nominate a mental health lead, who is likely to be the SENCo (as children with ADHD and associated difficulties currently fall under their remit in the UK) and will be referred to as such henceforth. Decisions around recruitment and eligibility criteria have been made in close consultation with the study planning group.

As part of the condition of each schools' participation, the senior leadership team will be asked to ensure that the SENCo and potentially eligible teachers and TAs (those working with recruited children) are supportive of and potentially willing to take part in the study.

Identification and recruitment of eligible children and families

Eligible children will either have a clinical diagnosis of ADHD (as reported by the school or parents), or high symptom levels and will be aged 4 to 10 (school years Reception-5) at the first baseline assessment to ensure they will be in primary school for the study duration. Children with ADHD may either have not yet received a formal diagnosis, or their family may not wish to pursue a diagnosis, therefore, children with high symptom levels will be eligible. There are no additional exclusion criteria. Teachers and SENCos will identify students who meet criteria for this indicated group using the Strengths and Difficulties Questionnaire hyperactivity-inattention subscale and the impact

For peer review only - http://bmjopen.bmj.com/site/about/guidelines.xhtml

supplement.[29] Teachers of potentially eligible children will be asked by the SENCo to complete this brief screening measure, and if this indicates that a child may have probable ADHD (teacher-reported symptoms being ≥6 and impact ≥1), their parent will also be asked to complete the screen in order to ensure that impairments are noted across more than one setting.[2 30] Should these combined ratings indicate 'probable' ADHD according to the validated algorithm, the child will be eligible for inclusion in the study. Probable ADHD is assigned if the parent-report hyperactivity/inattention score is ≥7 and an impact score of 2 is given, or if the hyperactivity/inattention score is ≥9 and an impact score of at least one is reported, and the teacher criteria above are met (see sdqinfo.org for syntax).

SENCos will make initial contact with parents of children to clarify eligibility, to provide the study information and to establish whether families are interested in participation in principle. Willing families will be asked to consent to having their details passed to the research team, who will obtain written informed consent from parents and assent from children to participate. Children will be informed of the broad purpose of the research through an age-appropriate information sheet and assent form, unless their parents request otherwise. Should an individual child not meet eligibility criteria but their parents and teacher feels they would benefit from participation, this will be considered on a case-by-case basis. As the intervention is embedded as part of usual school practice, the planning group has indicated that ongoing assent should then be assumed for children through data collection unless the child indicates distress or reluctance.

Sample size

The planning group has advised that to minimise burden of data collection on teachers, each class teacher should have a maximum of one child participant; however this may be revised during the course of the study, for example if a school feels strongly that several children in one class may benefit, or if two eligible children in the same class also have TA support and the teacher wishes both to participate. Estimating that each of the eight participating schools will have 2-4 participating

For peer review only - http://bmjopen.bmj.com/site/about/guidelines.xhtml

children and teachers, there will be a total of 16-32 child participants, 16-64 parents, and 16-64 teachers and TAs along with eight SENCos. The total sample size will depend on the distribution of participating parents, teachers and teaching assistants and the distribution of eligible children across different classes within schools: Table 1 indicates the minimum and maximum sample sizes.

Given that the aim of the study is not to assess effectiveness or efficacy, the potential for the intervention to change between participants, and the case-series design, this study will not be powered to conduct a conventional between-individuals analysis. Instead, we will use multiple individual measures to analyse within-individual change and assess whether this replicates (at a descriptive level) across individuals. The pragmatic choice of eight schools allows for this purposive sampling, and both the minimum and maximum sample size of children is sufficient to address our objectives. Based on the mean number of children in eligible year groups in Devon schools, and a conservative estimate of prevalence of ADHD in this age group of approximately 3%; it is expected that each school will have 4-8 eligible children, of whom we hope to recruit at least 50%. The recruitment target is feasible: school staff have indicated that a toolkit for ADHD would be perceived as useful and not burdensome as they believe it would be beneficial to their wider classroom management (the planning group highlight the data-collection burden as being the reason that teachers should only have one participating student per class).

For peer review only

Table 1. Sample size minimum and maximum

Participant type	Minimum number	Maximum number
School	6	8
Children	16	32
Parents or carers	16	64 (i.e. 2 parents per child)
SENCos	8	8
Teachers	16	32
TAs	0	32

*maximum number based on every eligible school recruiting four pupils, each of which have a different class teacher. We anticipate actual numbers of TAs will be lower than the maximum.

An established set of study progression criteria are shown in Table 2 and will be used to assess success in terms of meeting the study aims. Criteria relate to the recruitment of schools (five or more being acceptable); recruitment of parents and teachers, and completion of study measures as well as some basic metrics of fidelity. The criteria will be reviewed throughout the study, and where individual criteria are Amber or Red, a core academics team meeting and a planning group meeting will be held to discuss, agree and then implement modifications to the study procedure or content.

Procedure

The study will run from September 2022 until January 2025. Data will primarily be collected at the participating schools, however interviews with parents and school staff may take place over the phone, via internet video call, at participants' homes, or in another location of participant's convenience. Baseline length will be one school term. During the baseline phase, the teacher will provide repeated measures of the primary quantitative outcomes: participating children's ADHD

For peer review only - http://bmjopen.bmj.com/site/about/guidelines.xhtml

symptoms and classroom functioning. These will be collected every two weeks during the baseline period and in the intervention period. This detailed baseline data will capture normal fluctuations in symptoms across the term and response to any school-implemented 'treatment as usual' during the baseline phase.

Information on healthcare and education resource use will be collected from children, parents, and school staff for each child during the baseline period using a structured draft survey with open questions to capture any resources not mentioned, and child quality of life will be reported by the child and their parent. Any other support or treatment will not be altered during the study. Each school will then implement the intervention for one school term; two modules from the intervention will each be implemented for four weeks. There will be a ten-week

follow-up period where detailed qualitative data will also be captured, including asking participants about changes in resource and service use relative to the information they provided at baseline.

For peer review only

For peer review only - http://bmjopen.bmj.com/site/about/guidelines.xhtml

Table 2. Study progression criteria

	Green: acceptable	Amber: discuss, modify	Red: redesign	Assessing
Recruitment of schools	6 or more	5	4 or fewer	Feasibility
Recruitment- teachers, children, parents (considered as separate groups)	>65%	20-65%	<20%	Feasibility
Retention of schools, teachers, children and parents in study	>65%	40-65%	<40%	Feasibility
Training completed (teachers)	>90%	70-90%	<70%	Acceptability and feasibility
Introductory video watched (parents)	>50%	20-50%	<20%	Acceptability
Child strengths activity completed	>50%	20-50%	<20%	Acceptability
Adherence to digital Daily Report Card	>70%	50-70%	<50%	Acceptability
Teacher-completed measures	>70%	50-70%	<50%	Feasibility
Parent-completed measures	>50%	20-50%	<20%	Feasibility
Child-completed measures	> 70%	50-70%	<50%	Feasibility
Observational measures	>70%	50-70%	<50%	Feasibility
Attendance at toolkit-related meetings	>75%	40-75%	<40%	Acceptability
Percentage of occasions toolkit reportedly used as instructed	>75%	50-75%	<50%	Acceptability and feasibility

For peer review only

For peer review only - http://bmjopen.bmj.com/site/about/guidelines.xhtml

Follow-up qualitative data collection completed (for each type of respondent: school staff, parents, child)	>50%	20-50%	N/A	Feasibility

Intervention delivery

The intervention is currently in development. Broadly, it will take the form of a digital toolkit of training and resource packages and behavioural strategies for non-specialists to use, organised within 'modules' that cover different classroom-based problems common to ADHD: we anticipate that each child participant will receive two modules. SENCos will have the role of co-ordinating and supporting teachers and TAs to deliver the toolkit in conjunction with support from parents at home, and given agency over the duration, dose and discontinuation of strategies. Supplementary Figure 1 illustrates the participant structure and roles within a school (see Supplementary material).

Intervention description

The Tools for Schools *FLEX* toolkit will primarily be a digital resource. The current logic model is shown in Figure 2 and the toolkit outline is shown in the Supplementary material (Supplementary Figure 2). The key goals of the toolkit that will foster sustainable behaviour change are to:

1. **Understand** the child and their communications

2. **Adapt** the classroom and school expectations

3. **Support** the child to:

    A- reduce acute impairments

    B- increase skills

    C- improve self-identity

through providing strategies that change the environment and expectations around the child.

For peer review only - http://bmjopen.bmj.com/site/about/guidelines.xhtml

For peer review only

4. This results in the child being able to consciously monitor and self-regulate

5. **Sustain** and adapt across time

<INSERT FIGURE 2 HERE>

Table 3 shows the data collection schedule relative to the stage within the study. A full intervention description can be found in the Supplementary Material.

Qualitative data collection

Toolkit iteration feedback

The toolkit will go through several iterations, refined based on ongoing data collection and analysis. Flexibility allowing for this is a strength of the study design.[31] Brief semi-structured 'feedback sessions' will be held by phone or in person with all participating school staff and parents during the intervention period, in order to understand where modifications need to be made to the design, content and measures of the intervention. Example questions from the topic guide for teachers are included in the Supplementary Material. All changes made from the original prototype of the toolkit will be recorded in a change log, including the reason for the change, when the change was made and what aspects of the toolkit the change relates to. The planning group and the study advisory group will convene to discuss feedback or major suggested modifications, and to review minor changes in the change log.

Process evaluation

During the ten-week follow-up period, detailed qualitative data will be collected to assess acceptability, perceived usefulness, fidelity and implementation of the intervention, exploring in more depth what participants did and did not do and explanations of their actions, with questions similar to those shown above. Focus groups comprising the constellation of all school staff involved with one child will be held for all participants. This format is familiar to school staff as professional discussions often take a 'case conference'

For peer review only

For peer review only - http://bmjopen.bmj.com/site/about/guidelines.xhtml

format. Interviews by phone or an online platform such as zoom will be conducted with a purposive sample of half of the parents, using the same criteria detailed above (i.e. at least 50% from areas with a deprivation index above the regional mean) and aiming to attain a balance of male and female parents. Children will take part in 'active interviews' during the process evaluation in the follow-up period with a researcher (at home or at school), where they will have an informal conversation about their experiences of the study, if they recalled any new things their teacher tried with them, how they felt about their behaviour targets (if they knew about them), and how they found the research process.

For peer review only

For peer review only - http://bmjopen.bmj.com/site/about/guidelines.xhtml

Table 3. Data collection schedule and toolkit implementation timeline

School terms	Baseline term													Intervention term													Follow-up term												
**Week**	**1**	**2**	**3**	**4**	**5**	**6**	**7**	**8**	**9**	**10**	**11**	**12**	**13**	**1**	**2**	**3**	**4**	**5**	**6**	**7**	**8**	**9**	**10**	**11**	**12**	**13**	**1**	**2**	**3**	**4**	**5**	**6**	**7**	**8**	**9**	**10**	**11**	**12**	**13**
**Toolkit steps**																																							
Know ADHD											X	X	X																										
Know me											X	X	X																										
Understand motivations and develop targets													X	X	X																								
Establish digital school-home notebook														X	X																								
Map targets to modules and choose strategies														X	X																								
Implement strategies (module 1)															X	X	X	X	X	X																			
Implement strategies (module 2)																					X	X	X	X	X	X													
**Study measures (data collection)**																																							
Measures of ADHD symptoms	X		X		X		X		X		X		X		X		X		X		X		X		X		X			X			X			X			X
Measures of classroom functioning	X		X		X		X		X		X		X		X		X		X		X		X		X		X			X			X			X			X
Toolkit iteration feedback (qualitative)																		X								X													
Process evaluation: fidelity, acceptability, perceived usefulness																											X	X	X	X	X	X	X	X	X	X			
Child dimensional psychopathology and school functioning	X											X							X							X											X		
Child-reported satisfaction with school	X											X							X							X											X		
Teacher wellbeing	X											X							X							X											X		
Healthcare and education resource use	X											X															X												X

For peer review only - http://bmjopen.bmj.com/site/about/guidelines.xhtml

Child quality of life	X											X															X												X

Note: Supplementary material contains information on the Toolkit steps. Measures are: ADHD symptoms: the Strengths and Weaknesses of ADHD and Normal Behavior Scale (SWAN); Classroom functioning: problem behaviour subscale of the Social Skills Improvement System (SISS); Dimensional psychopathology and school functioning: the Strengths and Difficulties Questionnaire and the academic competence and social skills subscales of the SISS; Satisfaction with school: the How I Feel About My School questionnaire; Teacher wellbeing: the Warwick-Edinburgh mental wellbeing scale 14-item Teacher survey, and the Relationship with Work survey from the Maslach Burnout Inventory- General Survey, and the Teacher Sense of Efficacy Scale; healthcare and education resource use is a bespoke questionnaire available from the authors on request; and Child quality of life is measured by child-report and parent proxy Child Health Utility-9D.

For peer review only - http://bmjopen.bmj.com/site/about/guidelines.xhtml

For peer review only

All children will be invited to an activity- based interview with the option of a paired interview, where they can bring an accompanying family member of their choosing. Paired interviews can reduce stress, mitigate child protection risks and reduce issues of running a focus group where children in the school with traits of ADHD are identified to one another. The activity interviews will be designed with the planning group and piloted with children with ADHD, for example these may involve collecting objects of different colours or taking photos around the school to represent answers to questions (such as *can you show me where you like to go if you feel you need to calm down? What are your favourite things about school? What do you not like about school?)*, and using these objects or photos as prompts to discuss thoughts.

All participants in the process evaluation will be asked what they think the most important outcome would be for a future hypothetical trial of the toolkit, and why.

Adverse effects of intervention

It is possible that the intervention will lead to adverse effects or negative impacts on participating children, such as feeling singled out due to the intervention leading to social exclusion from the peer group.[32] The study planning group will contribute to identifying potential adverse effects *(as of March 21 social exclusion is the most prominent concern*), and qualitative data will be collected from participants at the feedback and process evaluation stages to assess perceptions of negative impacts of the intervention. Modifications will be made as needed in relation to this through consultation with the planning group and the academic advisory team.

Quantitative measures

There are two quantitative outcome measures that will be assessed in order to: 1) determine which would be most suitable as the primary outcome in a future trial and 2) evaluate whether they make sense and are relevant to those completing the measures. **ADHD symptoms** will be measured using the Strengths and Weaknesses of ADHD symptoms and Normal-behaviour (SWAN) questionnaire, based on preferences of the planning group who considered several measures of ADHD symptoms,[33]

For peer review only - http://bmjopen.bmj.com/site/about/guidelines.xhtml

[34] reported by teachers; and **classroom functioning problem behaviour** subscale measured using the Social Skills Improvement System (SISS),[35] that captures social, academic and competing problem behaviours in the classroom environment, reported by parents and teachers. These will be measured every 2 weeks during the baseline and intervention period and every 3 weeks during the follow-up period. All planned quantitative measures are detailed in a table in the Supplementary material.

Other quantitative measures will be captured to assess whether there is indication of some improvement in the key areas the toolkit aims to target. Measures relating to children include **child dimensional psychopathology**, assessed using the teacher and parent-reported Strengths and Difficulties Questionnaire,[30] and **child-reported satisfaction with school** using the How I Feel About My School measure.[36] **Teacher wellbeing** will be measured using the Warwick-Edinburgh mental wellbeing scale 14-item Teacher survey [37] the Teachers' Sense of Efficacy Scale,[38] and the Relationship with Work Survey from the Maslach Burnout Inventory- General Survey.[39 40] ; and the social skills and academic competence subscales from the SISS will be completed by teachers. These will be collected at the beginning and end of the baseline period, at the end of the intervention term and at the end of the follow-up term. Other module-specific measures which are not covered by the above measures will be administered depending on the modules used by each child, e.g. the Harter Self-Perception Profile for Children to measure self-esteem.[41]

Methods for collecting data on **healthcare and education resource use** and **health-related quality of life** will also be established and tested. Child quality of life (child-report and parent-proxy) will be measured using the Child Health Utility for Economic Evaluation (CHU9D).[42] A tool to accurately capture education and healthcare resource use will be developed for the study, drawing on the Client Service Receipt Inventory [43] and measures in the Database of Instruments for Resource Use Management Repository. Participants will complete this at baseline and follow-up, and provide qualitative information on changes in resource use during the follow-up process evaluation. A data

For peer review only - http://bmjopen.bmj.com/site/about/guidelines.xhtml

collection form will also capture data on resource use and costs associated with the set-up and delivery of the intervention.

Observational measures of child behaviour in the classroom will also be tested throughout the study. These include but are not limited to the Classroom Observation Code,[44] and the Teacher-Pupil Observation Tool.[45] Observations will be carried out at school by members of the research team, trained to an acceptable reliability (0.7 or higher).

All data will be collected online via secure servers, or on paper, and manual data entry will be double checked.

Analysis

Aim 1. To assess whether the toolkit is feasible and acceptable for schools to implement , we will use the definition of acceptability proposed by Sekhon et al (2017): "a multi-faceted construct that reflects the extent to which people delivering or receiving a healthcare intervention consider it to be appropriate, based on anticipated or experienced cognitive and emotional responses to the intervention". Qualitative data from both the toolkit iteration interviews and the process evaluation interviews and focus groups will be audio recorded, transcribed and analysed using the Framework method and Thematic analysis.[46] An initial coding framework will be developed after reading and re-reading transcripts. Deductive codes derived from the research questions and inductive codes within the data will be identified. This framework will be applied independently to three transcripts; discrepancies in coding will be discussed and revisions made to the framework which will then be applied to the remaining transcripts, with further discussion and revision where necessary.

Responses relating to each component of the toolkit will be synthesised in a framework in order to assess the acceptability of the evolving and final prototype; specifically to assess whether the components and the overall toolkit are considered appropriate to support children with ADHD in school. In addition, acceptability of the toolkit will be quantitatively assessed against the study progression criteria that relate to completion of each component of the toolkit, attendance at

For peer review only - http://bmjopen.bmj.com/site/about/guidelines.xhtml

toolkit-related meetings, and the percentage of occasions the participant reports using the toolkit as instructed. The qualitative findings will be used to explain and further explore quantitative results in a mixed methods synthesis. Modifications will be made iteratively as findings emerge, and by the end of the study each indicator should be in the “green” range in order to indicate that the toolkit is acceptable.

Aim 2. To establish whether a future trial of efficacy is feasible, data on the research process and participant compliance will be evaluated. The quantitative data on recruitment and retention of participants, completion of measures by each participant group (parents, child, teacher, observed) and adherence to the intervention protocol will be assessed relative to the relevant progression criteria described in Table 2. These have been informed by existing school-based trials.[47 48] As above, qualitative findings from the process evaluation and toolkit iteration feedback will be integrated to explain quantitative findings. Should all relevant progression criteria be rated “green”, a future trial of efficacy will be considered feasible. Should criteria be amber or red as the study progresses, potential mitigating modifications will be discussed, such as removing the requirement for parents to actively engage with the toolkit.

Sub-aim a: In order to identify suitable outcome measures to assess core ADHD symptoms, child and teacher wellbeing and identify an appropriate primary outcome for a future definitive trial the acceptability and feasibility of using the individual measures will be evaluated following the steps above and in relation to the threshold set in the progression criteria. Measures will be considered suitable if there is a high level of compliance with completion (i.e. each meet the green criteria), and qualitative findings indicate no major barriers to completing the measure on repeated occasions. Should measures be identified as unsuitable between toolkit iterations, new measures will be identified with the planning group and the data collection plan modified. For example, if the ADHD symptoms measure is too burdensome to complete as frequently as required, other briefer ADHD symptom scales will be explored, or the data collection frequency may be modified. The two versions of the CHU9-D will be compared to assess whether child-report is sufficient. To **identify an**

For peer review only - http://bmjopen.bmj.com/site/about/guidelines.xhtml

appropriate primary outcome for a future trial, participants' qualitative responses to direct questions regarding what they think the most important outcome should be (and why) collected during the process evaluation will be synthesised. Findings will be discussed with the planning group and advisory team prior to deciding on the final primary outcome measure.

Sub-aim b. In order to test a framework for costing the toolkit, and for assessing cost-effectiveness in a future definitive trial, the detailed information collected from the draft framework will be assessed following baseline data collection, and modifications made to the framework where free-text responses indicate additional resources not captured in the draft framework. The process evaluation interviews and focus group data will also contribute to understanding changes in resource use, which will be aligned with the follow-up responses regarding resource use in order to ascertain whether the framework is adequate and sensitive to changes in resource use.

The data captured on a bespoke form relating to the costs of setting up and delivering the toolkit will be assessed to expand the framework for accurately costing the toolkit's delivery and potential savings, and the costs of delivering the prototype intervention will be calculated (including costs for staff time, training and delivery of components). This framework will be revised in line with study findings with the aid of health economists, with a final framework developed by the end of the study that would allow for cost-effectiveness to be formally assessed in a future definitive trial.

Sub-aim c. Assess whether measures of behaviour, classroom functioning and teacher-reported ADHD symptoms indicate improvement following use of the toolkit. **To explore whether there is indication that the toolkit may be efficacious**, quantitative data will be visually analysed within schools and participants, describing the nature of change over time and in relation to the intervention, following recommendations.[31] Non-parametric analysis of the data will be considered, for example by calculating Tau-U statistics. As the toolkit will be refined between schools' participating, it would be irrelevant to compare measures across individuals who received different iterations of the toolkit; the focus will therefore be on assessing whether there is initial indication of

For peer review only - http://bmjopen.bmj.com/site/about/guidelines.xhtml

improvement in outcomes, and whether any effect is replicated across individuals. Comparisons between the magnitude of change seen between those who receive earlier and later iterations of the toolkit will be carried out as it is anticipated that later iterations, modified to be more acceptable and feasible, would result in greater evidence of behaviour change. This will be an exploratory analysis. The core and target behaviours chosen across study participants will also be explored in a descriptive manner in order to assess the use of the behaviour web, targets and modules.

Missing, unused and spurious data and inclusion in analysis

All data will be utilised in analysis. Patterns of missing quantitative data will be evaluated as part of assessing the feasibility and acceptability of the intervention. Missing data could relate to the feasibility and acceptability of the intervention, or the utility and acceptability of the measure itself (i.e. Aim 1 and sub-aim 1), and so through the qualitative data collection we will aim to elicit how patterns of missing data relate to our aims and make modifications accordingly.

Ethics and dissemination

Ethics and governance approval

Ethical approval has been awarded by the University of Exeter College of Medicine and Health Research Ethics Committee (Ref Jan22/B/300). Informed consent will be sought from all adult participants (and by parents for child participants; an example information sheet and consent form is provided in the Supplementary Material). Children will provide assent and ongoing assent will be judged by the research team throughout. Should it become clear that a child does not want to continue to participate in data collection or the study, withdrawal will be discussed with parents and school staff. Should a child express that they wish to stop during a single instance of data collection, this will be stopped for that day. The child's feelings will be respected and discussed with the child, their parents and school staff about their future involvement. Ultimately, decisions will be made in line with the best interests of the child. All data collected will comply with General Data Protection Regulation (GDPR) data protection regulations, being stored on secure servers and only accessible by

For peer review only

the research team. Some data will be shared as part of the intervention, for example teachers' ratings of ADHD symptoms will be viewable by the SENCo and express permission will be sought for any planned data sharing between participants. Participants will be asked whether their data can be made available following the study for future analysis or related research. Those that consent will contribute their data to a fully anonymised dataset accessible to bona fide researchers. There are several operational issues and associated risks that are involved in performing this study. It is possible that the intervention will lead to adverse outcomes [32] such as study children feeling singled-out from their peers due to the intervention. The planning group will discuss anticipated potential adverse outcomes due to the intervention, and qualitative feedback will be sought from participants both during their use of the intervention and in the follow-up period. Further risks (aside from those detailed in progression criteria in Table 2) have been identified and mitigation plans detailed, as well as noting key study limitations (see Supplementary material).

Dissemination

Participants who consent to their anonymised data being shared for future research purposes will have their data archived in an open access dataset. The results from the study will be presented at relevant national and international conferences (e.g. the ADHD World Congress, Eunethydis, the Association for Child and Mental Health national conferences). Separate publications will describe: the iterative development process, the main outcomes of the case-series study, development of the resource use tool, and an in-depth qualitative analysis of data collected during the iteration and process evaluation; authorship will be determined using the ICJME criteria. A detailed dissemination plan and documents will be developed with the planning group and will include blogs and podcasts, recorded and live talks and events and engagement activities to reach a broader audience of interested stakeholders and policymakers.

For peer review only - http://bmjopen.bmj.com/site/about/guidelines.xhtml

Declarations

Data Statement

Not applicable at this time as this is a study protocol. All participants will be asked for consent to store and share their data in a publically-accessible anonymised dataset.

Should amendments be made to the study protocol after publication, we will advise the publishing journal and publish a note of amendment.

Author Contributions

AR, DM and TF conceptualised the work; AR, BD, DM, RH, ESB, LP, TF, JK designed and drafted the research programme, led by AR. AR drafted the manuscript, and BD, DM, RH, ESB, LP, TF and JK critically revised the final manuscript. This is protocol version 1.2, November 2022.

Acknowledgements

Additional contribution was made by Suzie Holt, Eleanor Bryant, Becky Gudka and Charlotte Kelman of the Exeter University ChYMe (Children and Young People's Mental Health (ChYMe) research collaboration. We would also like to thank the members of the Tools for Schools planning group for their ongoing collaboration (https://blogs.exeter.ac.uk/toolsforschools/about-us/planning-group/).

Competing Interests

All authors declare that they have no competing interests other than those disclosed in the funding section below.

Funding

This report is independent research supported by the National Institute for Health Research NIHR Advanced Fellowship - Stage 2, Dr Abigail Russell, grant ref NIHR300591. The views expressed in this publication are those of the author(s) and not necessarily those of the NHS, the National Institute for

For peer review only - http://bmjopen.bmj.com/site/about/guidelines.xhtml

Health Research or the Department of Health and Social Care. Tamsin Ford receives funding from Place2Be, a children's mental health charity (grant ref: N/A).

References

1. Parliamentary House of Commons. Children and young people's mental health - policy, services, funding and education (07196), 2017.

2. American Psychiatric Association. Diagnostic and statistical manual of mental disorders (DSM-5®): American Psychiatric Pub 2013.

3. Tarver J, Daley D, Sayal K. Attention-deficit hyperactivity disorder (ADHD): an updated review of the essential facts. *Child Care Health Dev* 2014;40(6):762-74. doi: 10.1111/cch.12139 [published Online First: 2014/04/15]

4. Gore FM, Bloem PJ, Patton GC, et al. Global burden of disease in young people aged 10–24 years: a systematic analysis. *The Lancet* 2011;377(9783):2093-102.

5. Telford C, Green C, Logan S, et al. Estimating the costs of ongoing care for adolescents with attention-deficit hyperactivity disorder. *Social psychiatry and psychiatric epidemiology* 2013;48(2):337-44.

6. Wehmeier PM, Dittmann RW, Banaschewski T. Treatment compliance or medication adherence in children and adolescents on ADHD medication in clinical practice: results from the COMPLY observational study. *ADHD Attention Deficit and Hyperactivity Disorders* 2015;7(2):165-74.

7. Taylor E. ADHD medication in the longer term. *Zeitschrift für Kinder-und Jugendpsychiatrie und Psychotherapie* 2019

8. Bates G. Drug treatments for attention-deficit hyperactivity disorder in young people. *Advances in psychiatric treatment* 2009;15(3):162-71.

9. Galvez-Contreras AY, Vargas-de la Cruz I, Beltran-Navarro B, et al. Therapeutic Approaches for ADHD by Developmental Stage and Clinical Presentation. *International Journal of Environmental Research and Public Health* 2022;19(19):12880.

For peer review only - http://bmjopen.bmj.com/site/about/guidelines.xhtml

10. Cortese S, Adamo N, Del Giovane C, et al. Comparative efficacy and tolerability of medications for attention-deficit hyperactivity disorder in children, adolescents, and adults: a systematic review and network meta-analysis. *The Lancet Psychiatry* 2018;5(9):727-38. doi: https://doi.org/10.1016/S2215-0366(18)30269-4

11. Fleming M, Fitton CA, Steiner MFC, et al. Educational and Health Outcomes of Children Treated for Attention-Deficit/Hyperactivity Disorder. *JAMA pediatrics* 2017;171(7):e170691. doi: 10.1001/jamapediatrics.2017.0691 [published Online First: 2017/05/02]

12. Excellence NIfHaC. Attention deficit hyperactivity disorder: diagnosis and management. *Attention deficit hyperactivity disorder: diagnosis and management* 2019

13. Greene RW, Beszterczey SK, Katzenstein T, et al. Are students with ADHD more stressful to teach? Patterns of teacher stress in an elementary school sample. *Journal of Emotional and Behavioral Disorders* 2002;10(2):79-89.

14. Fabiano GA, Pelham WE, Jr., Coles EK, et al. A meta-analysis of behavioral treatments for attention-deficit/hyperactivity disorder. *Clinical Psychology Review* 2009;29(2):129-40.

15. Richardson M, Moore DA, Gwernan-Jones R, et al. Non-pharmacological interventions for attention-deficit/hyperactivity disorder (ADHD) delivered in school settings: systematic reviews of quantitative and qualitative research. *Health Technology Assessment (Winchester, England)* 2015;19(45):1-470.

16. Catalá-López F, Hutton B, Núñez-Beltrán A, et al. The pharmacological and non-pharmacological treatment of attention deficit hyperactivity disorder in children and adolescents: a systematic review with network meta-analyses of randomised trials. *PloS one* 2017;12(7):e0180355.

17. Moore DA, Russell AE, Matthews J, et al. School-based interventions for attention-deficit/hyperactivity disorder: A systematic review with multiple synthesis methods. *Review of Education* 2018;6(3):209-63.

For peer review only - http://bmjopen.bmj.com/site/about/guidelines.xhtml

18. Veenman B, Luman M, Oosterlaan J. Efficacy of behavioral classroom programs in primary school. A meta-analysis focusing on randomized controlled trials. *PloS one* 2018;13(10):e0201779.

19. Fabiano GA, Pyle K. Best Practices in School Mental Health for Attention-Deficit/Hyperactivity Disorder: A Framework for Intervention. *School Mental Health* 2019;11(1):72-91.

20. Wiener J. The role of school psychologists in supporting adolescents with ADHD. *Canadian Journal of School Psychology* 2020;35(4):299-310.

21. Bartholomew LK, Parcel GS, Kok G. Intervention mapping: a process for developing theory and evidence-based health education programs. *Health education & behavior* 1998;25(5):545-63.

22. Eldredge LKB, Markham CM, Ruiter RA, et al. Planning health promotion programs: an intervention mapping approach: John Wiley & Sons 2016.

23. Kok G, Gottlieb NH, Peters G-JY, et al. A taxonomy of behaviour change methods: an Intervention Mapping approach. *Health Psychology Review* 2016;10(3):297-312. doi: 10.1080/17437199.2015.1077155

24. Perry RC, Ford TJ, O'Mahen H, et al. Prioritising Targets for School-Based ADHD Interventions: A Delphi Survey. *School Mental Health* 2021:1-15.

25. Kratochwill TR, Levin JR. Enhancing the scientific credibility of single-case intervention research: Randomization to the rescue. 2014

26. Morgan DL, Morgan RK. Multiple-baseline designs. *Single-case research methods for the behavioral and health sciences* 2009:125-61.

27. Kratochwill TR, Hitchcock J, Horner R, et al. Single-case designs technical documentation. *What works clearinghouse* 2010

28. Russell A, Ford T, Russell G. Barriers and predictors of medication use for childhood ADHD: findings from a UK population-representative cohort. *Social Psychiatry and Psychiatric Epidemiology* 2019:1-10.

For peer review only - http://bmjopen.bmj.com/site/about/guidelines.xhtml

29. Hall CL, Guo B, Valentine AZ, et al. The validity of the Strengths and Difficulties Questionnaire (SDQ) for children with ADHD symptoms. *PLOS ONE* 2019;14(6):e0218518. doi: 10.1371/journal.pone.0218518

30. Goodman R, Ford T, Simmons H, et al. Using the Strengths and Difficulties Questionnaire (SDQ) to screen for child psychiatric disorders in a community sample. *The British journal of psychiatry* 2000;177(6):534-39.

31. Morley S. Single case methods in clinical psychology: a practical guide: Routledge 2017.

32. Pfiffner LJ, DuPaul GJ. Possible adverse side effects of school behavioral interventions. *The ADHD Report* 2018;26(1):10-13.

33. Swanson JM, Schuck S, Porter MM, et al. Categorical and dimensional definitions and evaluations of symptoms of ADHD: history of the SNAP and the SWAN rating scales. *The International journal of educational and psychological assessment* 2012;10(1):51.

34. Brites C, Salgado-Azoni C, Ferreira T, et al. Development and applications of the SWAN rating scale for assessment of attention deficit hyperactivity disorder: a literature review. *Brazilian Journal of Medical and Biological Research* 2015;48(11):965-72.

35. Gresham F, Elliott SN. Social skills improvement system (SSIS) rating scales: San Antonio, TX: Pearson Education Inc 2007.

36. Allen K, Marlow R, Edwards V, et al. 'How I Feel About My School': The construction and validation of a measure of wellbeing at school for primary school children. *Clinical child psychology and psychiatry* 2018;23(1):25-41.

37. Tennant R, Hiller L, Fishwick R, et al. The Warwick-Edinburgh mental well-being scale (WEMWBS): development and UK validation. *Health and Quality of life Outcomes* 2007;5(1):1-13.

38. Tschannen-Moran M, Hoy AW. Teacher efficacy: Capturing an elusive construct. *Teaching and teacher education* 2001;17(7):783-805.

For peer review only - http://bmjopen.bmj.com/site/about/guidelines.xhtml

39. Langballe EM, Falkum E, Innstrand ST, et al. The factorial validity of the Maslach Burnout Inventory–General Survey in representative samples of eight different occupational groups. *Journal of Career Assessment* 2006;14(3):370-84.

40. Maslach C, Jackson S. Leiter MP Maslach burnout inventory manual. *Palo Alto: California Consulting Psychological Press Inc* 1996

41. Harter S. Self-perception profile for adolescents: Manual and questionnaires. *Denver, CO: Univeristy of Denver, Department of Psychology* 2012

42. Stevens K. Valuation of the child health utility 9D index. *Pharmacoeconomics* 2012;30(8):729-47.

43. Chisholm D, Knapp MRJ, Knudsen HC, et al. Client socio-demographic and service receipt inventory–European version: development of an instrument for international research: EPSILON Study 5. *The British Journal of Psychiatry* 2000;177(S39):s28-s33.

44. Abikoff H, Gittelman-Klein R, Klein DF. Validation of a classroom observation code for hyperactive children. *Journal of Consulting and Clinical Psychology* 1977;45(5):772.

45. Martin P, Daley D, Hutchings J, et al. The teacher-pupil observation tool (T-POT) development and testing of a New classroom observation measure. *School Psychology International* 2010;31(3):229-49.

46. Gale NK, Heath G, Cameron E, et al. Using the framework method for the analysis of qualitative data in multi-disciplinary health research. *BMC medical research methodology* 2013;13(1):1-8.

47. Pfiffner LJ, Rooney M, Haack L, et al. A randomized controlled trial of a school-implemented school–home intervention for attention-deficit/hyperactivity disorder symptoms and impairment. *Journal of the American Academy of Child & Adolescent Psychiatry* 2016;55(9):762-70.

48. Ford T, Hayes R, Byford S, et al. The effectiveness and cost-effectiveness of the Incredible Years® Teacher Classroom Management programme in primary school children: results of the STARS cluster randomised controlled trial. *Psychological medicine* 2019;49(5):828-42.

For peer review only - http://bmjopen.bmj.com/site/about/guidelines.xhtml

For peer review only

Figure legends

Figure 1. Case series design outline

Key- grey, baseline; green, intervention; blue, follow-up

Figure 2. Intervention Draft Logic Model

For peer review only

For peer review only - http://bmjopen.bmj.com/site/about/guidelines.xhtml

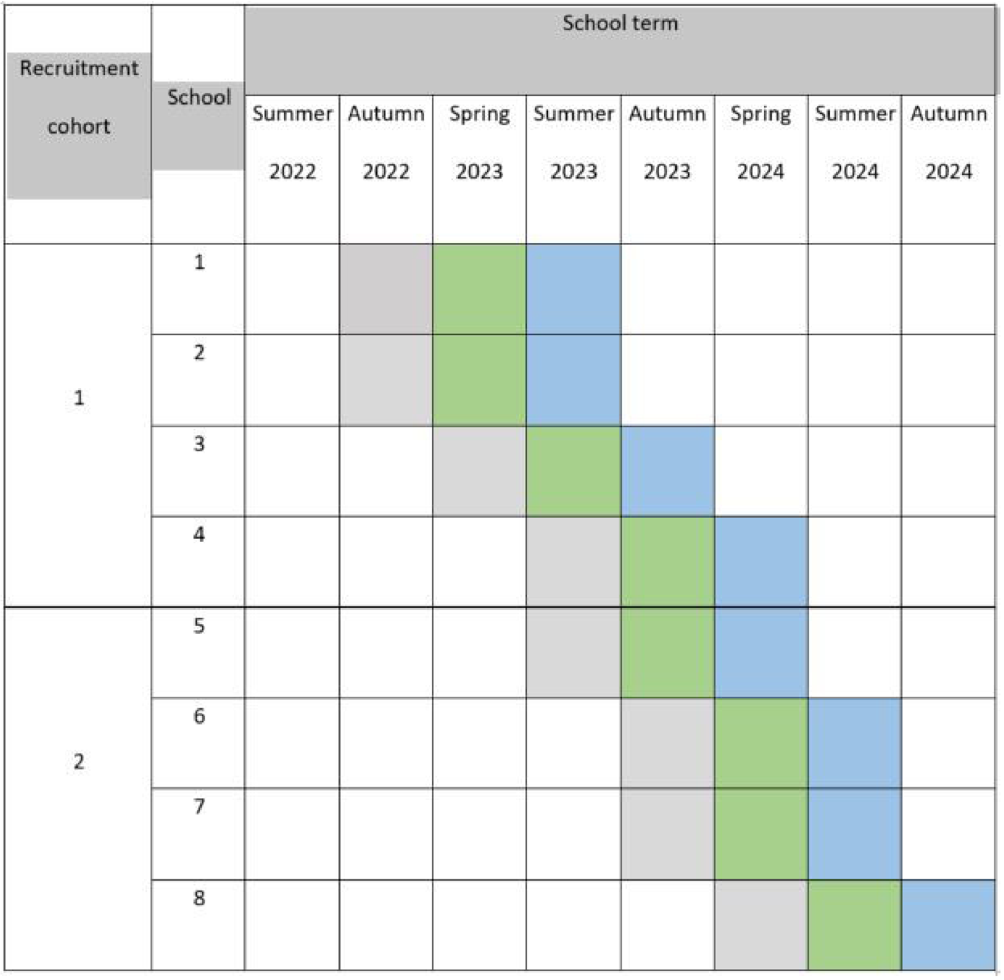

Figure 1. Case series design outline
Key- grey, baseline; green, intervention; blue, follow-up

52x51mm (300 x 300 DPI)

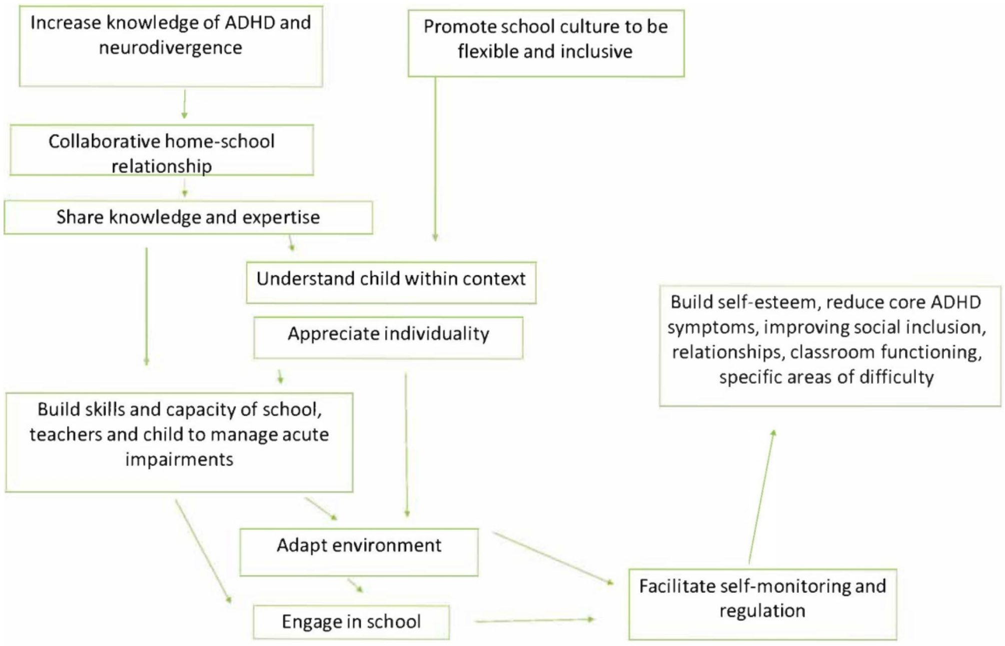

Figure 2. Intervention Draft Logic Model

169x109mm (300 x 300 DPI)

Protocol for an iterative case-series study to investigate the feasibility and acceptability of a school-based intervention for children with traits of ADHD: Supplementary Material

Full intervention description

The Tools for Schools *FLEX* toolkit will comprise a digital resource of training and resource packages and behavioural strategies organised within 'modules' that cover different classroom-based problems common to ADHD. See the main article for information on the key goals of the toolkit. Supplementary Figure 1 illustrates the participant structure and roles within a school for delivery of the toolkit.

Supplementary Figure 1. Structure of individuals within schools and participant roles in case series study

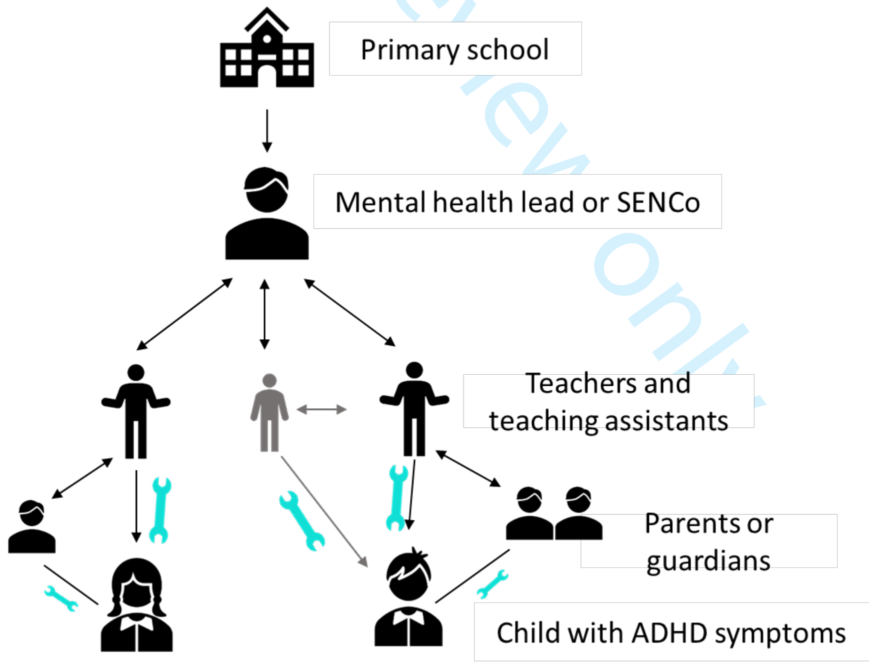

Delivery of the intervention comprises a series of steps, as indicated in the toolkit outline in Supplementary Figure 2, and described below.

For peer review only - http://bmjopen.bmj.com/site/about/guidelines.xhtml

Supplementary Figure 2. Outline of the *FLEX* Toolkit

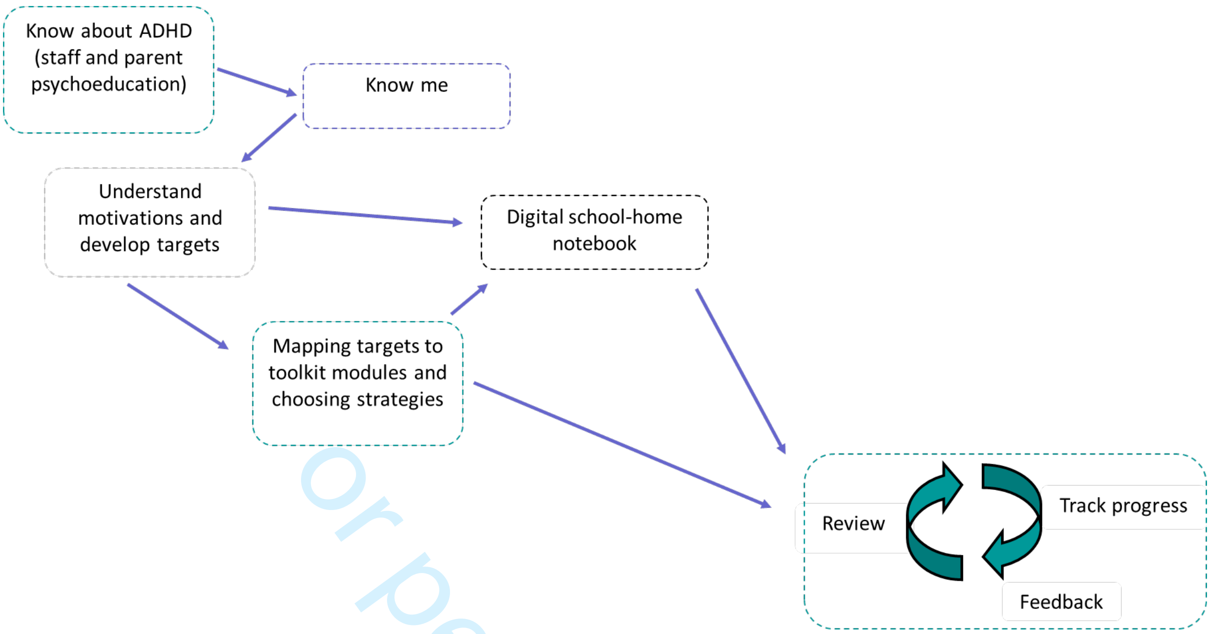

Step 1: Know ADHD

Purpose: Tailored knowledge of ADHD and relevant theory, psychoeducation. Putting staff and parents on equal footing, helping to address power dynamics between school and home and recognise expertise of each party

The toolkit will begin with online psychoeducation training for school staff and parents. This will cover ADHD as well as training on implementing the toolkit itself. Videos from people with ADHD, parents and school staff about their experiences will be included.

Key things the Know ADHD training will cover:

- ADHD is not an issue of morality or simply a deficit in attention
- Involvement of dopamine in the developing brain
- Executive functions and ADHD
- Delay aversion and ADHD
- Other theories about how ADHD traits come about
- Gender differences

For peer review only

- Sensory processing
- The variation between children with ADHD: every child with ADHD is different and unique, and their traits will affect them in different ways
- Growing up with traits of ADHD

Schools will also be asked to plan and deliver activities to promote inclusivity and the concepts of embracing individual differences and diversity at the outset of the study, through a participatory action research approach. Senior leadership and the SENCo will be asked to reflect on their school culture and inclusivity of school policies, make changes to these with the aim of improving flexibility around children with ADHD and their individual needs, and reflect on and evaluate these changes [23]. This might involve participating in national events such as Neurodiversity Celebration Week, having a school assembly or applying for the ADHD Foundation "ADHD friendly schools award" for example. Each school (senior leadership team [SLT] and SENCo) will decide on whether they want to implement any whole-school measures, and what form these will take. They will be supported by the research team but this is not an essential component of the toolkit, rather an exploratory component that will need to be led by each school themselves to be implementable or useful in future versions of the toolkit. The toolkit provides step-by-step instructions for the SLT to conduct an "Inclusion challenge" and makes suggestions as to what systems and processes within the school they may consider changing as part of this.

We will use the Participatory Action Research cycle of Plan, Act, Evaluate, Reflect to structure this. We ask the SLT to consider how their school could be more inclusive for neurodivergent children (including asking for input from children and others in the school) and plan how they could change this. Then they would implement the new ideas (act) for a given period of time; evaluate the changes by seeking staff and student views, and reflect on whether they were successful before implementing further changes. No data will be collected at awareness events or other activities the SLT decide to enact in this step, unless they collect it for their own evaluative purposes. Staff will be

For peer review only

asked to feedback about this during qualitative data collection instances. We anticipate researching the mechanisms of this potential component in further depth in separate research if participants report using this, implementing it and finding this component potentially useful.

Given the aim of this study is to assess feasibility and acceptability of implementation in a real-world context, we have made the decision not to make any component of the flex toolkit mandatory (although we strongly suggest that staff complete the ADHD training and the SLT do the inclusion challenge); the current UK school context is facing severe pressures and resource constraints, and we anticipate that uptake of components will be better and more representative of naturalistic use of the toolkit if they are not mandated. Whether or not to mandate this component in a future trial will be considered based on the findings of this study.

An additional consideration is how to present this information to parents whose child has traits of ADHD but is not diagnosed. The Step 1 learning is titled "know traits of ADHD" and uses minimal language about ADHD as a disorder, talking more about traits of poor attention or concentration, impulsive behaviour or acting without thinking, and hyperactivity. In the learning materials we include a segment on how this relates to the label or diagnosable disorder of ADHD. We are interested to obtain feedback from parents about this.

All of the parents in the study have had contact with their child's SENCo about the child's difficulties or perceived need for additional support in school prior to the study. When selecting and approaching parents, this is discussed expressly with the SENCo and they have a discussion with parents prior to the research team becoming involved. The study information sheet also talks about traits of ADHD, and parents are therefore aware of the nature of the toolkit before they get to Step 1.

For peer review only - http://bmjopen.bmj.com/site/about/guidelines.xhtml

Step 2: Know me (the child)

Purpose: Children with ADHD are more able to engage with something if it interests them. They often feel misunderstood by others and this activity is intended to allow staff to understand and empathise with the child, seeing things from their perspective. Children may not be able to verbalise their emotions and feelings and understanding how they behave when happy, sad, angry or excited is an important component of functional behaviour analysis and behaviour change approaches. This activity aims to draw these out for each individual.

Children will complete a strengths-focussed activity with the teacher or TA to identify strengths or hobbies that could be used to personalise the intervention and to help school staff understand the child from their perspective. This activity will be bespoke depending on what the child wants and likes to do and could be delivered to a whole class, but might include making a film or animation, creating a story or photo board, or building a virtual version of oneself on a computer game.

Step 3: Understand motivations and develop targets

Purpose of step: Sharing knowledge and expertise between school and home, focussing on understanding antecedents and consequences of behaviour in order to understand how best to modify. Mutually deciding on which targets are most important (and the relationships between potential targets)

At the end of the baseline term and the beginning of the intervention term, SENCos, teachers and other relevant staff will work alongside the child and family to complete a functional behaviour analysis of two problem areas where the child is currently struggling. We have chosen to constrain this to two areas in order to provide focus during the use of the toolkit in one school term for research purposes, and to support those testing the toolkit to identify the key areas for improvement and therefore observable change. Understanding the antecedents and consequences of behaviour will be crucial to identify where to intervene and to understand which modules and strategies from the toolkit are likely to be useful. The meetings will result in identifying two core

For peer review only

behaviours that will be transformed into the intervention "toolkit targets". The core problems will be operationalised into tangible and achievable goals (e.g. stay in seat for 5 minutes, with the number of minutes increasing as the child meets the target; having more than one friend to play with at lunchtime, starting with one day per week and increasing over time; having three adults at school they can go to if they have a problem, starting with one adult and knowing what and how to communicate to them if there is a problem) over the course of two meetings.

The form of these meetings may vary depending on parent engagement and preferences. One option that will be piloted is inviting families and children to join these meetings via videoconference. This would allow the child to feel involved in the process if they wish to engage, but also provides them with the option to withdraw from the conversation, and parents will make the decision as to whether they wish for their child to be involved in these meetings. Through discussions with the planning group, parents identified that they wish to be consulted and to bring their perspective to these meetings, however they may also find such meetings are difficult emotionally, as the focus will be on their child's difficulties. As such, parents may feel more empowered or comfortable joining from their home environment. Alternately, parents and children may wish to attend these meetings in person at school. Parents will make the decision as to where/through what medium the meeting takes place.

The purpose of this step is to encourage school staff and parents to become aware of the layers of context around the child, highlighting potential points for intervention that do not rely on intrinsic changes to the child themselves. This step of the intervention focusses on what achievements it is hoped the child will make, however the strategies that will be implemented to achieve these are primarily designed to adapt the environment around the child, for example scaffolding them with classroom tools and activities to improve organisation through making it clearer to the child where their possessions can be found or visual aids to demonstrate what will be needed for the next activity; introduction of whole-class games on identifying and providing positive feedback to peers

For peer review only

where all classmates are reinforced for "picking up the positives" to improve self-esteem; or incorporation of movement-based learning strategies throughout the school day to improve ability to concentrate, and the teacher changing the format of the work that the child is asked to complete.

Step 4: Digital school-home notebook

Purpose: Establishing and maintaining positive communication between teachers, TAs and parents. Research shows that conflictual and negative relationships between school and home are common for children with ADHD. Breaking negative communication cycles and establishing positive communication will improve sharing of information and ideas between home and school, and better support the child across contexts.

A communication platform for regular contact between staff and parents will be established once the child's toolkit targets have been agreed. This was initially intended to take the form of a digital daily report card (dDRC) with staff updating home daily on the child's progress with their targets, and parents reinforcing this at home. The purpose of this component is to develop habits of positive communication about progress and child strengths, rather than to function as a "behaviour log" or to report incidents during school.

The frequency and nature of communication using the notebook will be flexible to suit the needs of the individual child and participants surrounding them; the DRC format will be proposed as the suggested model as this has the most existing evidence for effectiveness [24 25]. However, other forms of successful communication may already be in place between school and home, or daily communication may be considered burdensome and not beneficial to both parents and teachers for a wide variety of reasons. As such, staff and parents will be asked to mutually agree on frequency of communication using the digital school-home notebook, and this will be reviewed and modified as required during the intervention period.

For peer review only - http://bmjopen.bmj.com/site/about/guidelines.xhtml

Step 5. Mapping targets to toolkit modules and choosing strategies

Purpose: identifying strategies within modules that are most likely to be effective based on functional behaviour analysis and understanding of the child

The SENCo and teacher will link the child's targets to the most pertinent modules and outcomes in the toolkit using a graphical map (currently called the behaviour web). This may be initially discussed in the two meetings to understand behaviour and develop targets, or may be decided following these meetings. The functional behaviour analysis will be drawn upon with a focus on the current consequences relating to the target behaviour, and alternate consequences that could encourage more adaptive behaviour.

The toolkit modules are based on individual ADHD-related outcomes selected by consensus in a Delphi study. They are:

1. Organising, planning and reminding (describe to differentiate from organising)->means self-monitoring and self-regulating
2. Paying attention
3. Feeling good (self-esteem, self-worth)
4. Belonging and relating (relationships)
5. Pausing for thought (inhibiting impulsive behaviour)
6. Channelling energy (hyperactivity)

Modules will contain a bank of approximately 10 non-pharmacological strategies that can be used at individual or classroom level to target the outcome, organised into domains such as "Behaviour management" "Training tools" and "Changing the classroom" through the co-creation process. The planning group have indicated that the focus of the toolkit should be on inclusivity: supporting children to remain in the classroom and allowing the child to access the same education and

For peer review only - http://bmjopen.bmj.com/site/about/guidelines.xhtml

opportunities as their classmates. Strategies will be developed with this goal in mind. In addition, modules will contain resources on maintaining strategies once implemented.

Step 6. Implementing and reflecting on strategies

Purpose: produce improvement in target behaviours that can be maintained

Once teachers and SENCos have determined the modules to implement through consideration of the functional behaviour analysis and the child's agreed targets, the SENCo will support the teacher and TAs to select and implement strategies. Strategies have been designed to be primarily group-based games or activities, or reminders and support for teachers to incorporate new techniques to adapt around the child, with the aim that this will lead to improved outcomes for the child. None of the strategies require the child to be removed from the classroom, and although some could be used 1:1 with the child, they are primarily presented as group-level activities to promote inclusion. A significant number of strategies focus on "belonging and relating", specifically aiming to promote peer inclusion, celebrate individual differences, and destigmatise neurodevelopmental (and other) differences.

For peer review only

We anticipate that one module will be implemented in each half-term of the intervention term, however there will not be a restriction on this, nor how many strategies within a module can be attempted; we anticipate that some strategies will have already been tried by a school or teacher, or it may be apparent that other strategies do not suit the child and so different strategies may be selected. Repeated measures of ADHD symptoms, classroom functioning, and measures specific to the outcomes relevant for each module will be collected from teachers, TAs and children as appropriate (Table 3 shows the data collection schedule relative to the stage within the study).

Co-production of the toolkit with the planning group

Parents, children with ADHD, adults with ADHD, SENCos, educational psychologists and teachers (the planning group) were initially informally interviewed about their experiences, and what they

For peer review only - http://bmjopen.bmj.com/site/about/guidelines.xhtml

perceive works for them. These findings were narratively synthesised and written up in one document.

They contributed to an exhaustive list of the kinds of behaviours that may form targets for the intervention, and the modules that these would fall within (e.g. being ‘easily led by others’ mapping to the ‘pausing for thought’ module); with some behaviours mapping to multiple modules depending on the underlying reason (e.g. ‘risky or dangerous behaviour’ mapping to either ‘paying attention and engaging’ or ‘pausing for thought’ depending on whether the primary reason is impulsivity or inattentiveness).

The intervention logic model of change was reviewed and refined with the planning group, who then had input into the performance objectives (the smallest steps of behaviour change required to meet each behavioural goal for each person e.g. teacher, child, senior leadership) through reviewing the lists in an ‘activity workbook’ format, adding thoughts and attending discussion meetings.

These performance objectives were also categorised by the determinants of behaviour (whether the change in behaviour required aligned with skills, values, beliefs, attitudes, knowledge or experience). Initial strategy ideas were then drafted by AR to match performance objectives with input from behaviour change taxonomies to select relevant delivery methods.

These draft strategy ideas were then reviewed by the planning group, and changed entirely or improved based on their input. Strategy instructions were then written by AR, reviewed by the planning group once more and refined and the prototype toolkit was produced. PDF resources to support teachers in delivering each strategy were generated by the research team, with the planning group suggesting resource ideas and providing further input and feedback at the ‘alpha testing’ stage of the prototype. Resources and strategies were then refined and finalised, prior to the beta version (to be used in the feasibility study) being rolled out.

For peer review only

For peer review only - http://bmjopen.bmj.com/site/about/guidelines.xhtml

Example questions for Toolkit iteration feedback interviews

1. *Could you tell me about how you have used the toolkit?*
2. *What have your experiences been of using the toolkit? Let's talk about it step-by-step so you can give me your reflections on each component. First, overall did you think the toolkit was useable in Primary school? Why/why not? And do you think other schools and families would find it acceptable? Why/why not? Do you think other schools and families would be able to implement it? Why/why not?*
3. *What did you like most about the toolkit? What did you like least? Could you tell me some more about that?*
4. *How was the Know ADHD component? Did you use it, what was good about it and what was difficult or challenging? What would help you to use it better in the future? Was there anything missing that you think is important for people to know about ADHD?*
5. *How was the Know Me component? What did you do with the child to complete this activity? What format did the child choose (e.g. video, storybook)? How did you feel about it, and how do you think the child felt about doing the activity? Did you find the product of this Know Me step was useful for the rest of the toolkit? Could you tell me why you think that? Would you make any changes to the Know Me step or instructions, and what would they be?*
6. *Who set up the meetings with school staff to assess and understand the two main problems? How did those meetings go and who was there? When did you have the meetings? What was good and bad about the meetings? Did you manage to define two target goals for the child? Were parents and the child involved (if so how)? Would you do anything differently if you did it again? Should the toolkit have different instructions? How did you understand the antecedents, behaviours and consequences of each behaviour? Who helped to figure those out, how and how long did it take? Was that too much time for you?*

For peer review only

Table of quantitative measures

Outcome	Measure
ADHD symptoms	Strengths and Weaknesses of ADHD and Normal Behavior Scale (SWAN)
Classroom functioning	Problem behaviour subscale of the Social Skills Improvement System (SISS)
Dimensional psychopathology	Strengths and Difficulties Questionniare
School functioning	Academic and social skills subscales of the SISS
Child-reported satisfaction with school	How I Feel About My School questionnaire
Teacher wellbeing	Warwick-Edinburgh mental wellbeing scale 14-item Teacher survey; the Relationship with Work survey from the Maslach Burnout Inventory-General Survey; the Teacher Sense of Efficacy Scale;
Health and education resource use	Bespoke questionnaire available from the authors on request
Child quality of life	Child and parent report Child Health Utility-9D
Additional module measures (where not captured by the above)	
Child self-esteem	Harter Self-Perception Profile for Children
Belonging and relating	PROMIS Pediatric Peer Relationships - Short Form 8
Organising, planning and reminding	Children's Organizational Skills Scale

For peer review only

Risks and mitigation plans

Risk 1. Schools are open but due to periods of Covid-related closure, child development and socialisation has been impacted.

Mitigation: as part of the toolkit development process we are liaising closely with primary schools, parents and teachers to monitor ongoing changes to school environments and cultures. We anticipate that, if anything, there will be greater demand for the toolkit if more pupils are struggling with relevant interpersonal and developmental challenges. There may be the need to adapt the focus of modules, for example, to support school attendance explicitly. Potential adaptations will be discussed with the academic advisory team and planning group before implementation.

Risk 2. Schools are closed due to ongoing Covid-19

Mitigation. The planning group has advised that many of the children meeting eligibility criteria for the study have attended school through prior lockdowns and closure periods due to their vulnerable status and additional needs. We will explore whether sufficient children and staff are attending school at the time of recruitment and data collection, and if so will continue as planned with implementation of the intervention. Data collection will be completed remotely through digital questionnaires and online video conferencing software meeting data protection requirements if external (i.e. research) staff are unable to access schools due to covid-19 lockdowns.

Risk 3. Unanticipated adverse effects of intervention

Mitigation: adverse effects will be monitored through qualitative data collection during the use of the intervention, and participating school staff will follow a defined procedure for reporting of negative impacts as soon as possible after they come to light. Should a child or other participant of the study report severe worsening of ADHD symptoms or distress, the PI will consult with clinically-trained members of the academic advisory board to decide on the most appropriate course of action. This may include: the clinical academic speaking directly to staff and families to ascertain whether the adverse effects are caused by the intervention or research study or are external to this, the child and family withdrawing from the study, or bespoke plans being put in place to monitor and manage the situation. Should a child or family withdraw from the study due to this, their existing data will not be used unless express consent is given at the time of withdrawal, and they will be able to continue to access the prototype intervention (as long as the study team are confident that this is not the cause of adverse effects) if they wish.

For peer review only - http://bmjopen.bmj.com/site/about/guidelines.xhtml

Limitations

This study is a feasibility study, and due to the iterative case-series design not all participants will get the same version of the study prototype. Therefore we will not be able to quantitatively measure or compare changes in our outcome measures across all study participants, instead we are restricted to examining whether individual trends of change are replicated across individuals.

In addition, due to the modular nature of the toolkit, the sample size for a future cluster randomised controlled trial may have to be large in order to obtain sufficient numbers of participants using each module.

For peer review only

Model consent form

Tools for Schools: a prototype toolkit for ADHD interventions to be used in primary school teaching.
Consent form for parents or guardians on behalf of children –Version 3: January 2022

I have read the provided information sheet, Version 4, concerning Tools for Schools project and I understand what it is about.
I fully understand what is expected of me and all my questions have been answered to my satisfaction.
I understand I am free to request further information at any stage.

As a **parent or guardian on behalf of my child,** I am aware that:

(please circle Yes or No)

1. My child's participation is entirely voluntary. Yes / No

2. I am able to withdraw my child's participation from the project without consequence or disadvantage. Yes / No

3. I understand that relevant sections of the data collected during the study may be looked at by members of the research team, and individuals from the University of Exeter where it is relevant to my taking part in this research. I give my permission for these individuals to have access to my records. Yes / No

4. The researchers do not anticipate any of the participants to experience any discomfort or harm during the project. Yes / No

5. I can contact the researchers with any questions, queries or worries at any point during the study (using the contact details provided in the information sheet). Yes / No

6. My child's data will be anonymous in any published works relating to the project. Yes / No

7. In the future, my child's anonymous data may be used in other related research. Yes / No

8. Recordings can be taken of interviews and class groups my child takes part in. Yes / No

For peer review only

For peer review only - http://bmjopen.bmj.com/site/about/guidelines.xhtml

9. My child will take part in lessons that may ask them to do things differently from other classmates. Wherever possible, this will be done in the most subtle way so as not to draw attention to my child, but I understand this may not always be possible.
   Yes / No

10. My child will be asked to complete questions that other children will not do. Yes / No

11. My child might be asked to leave the classroom to do different learning/lessons, for example daily report cards. Yes / No

12. My child might take part in an 'active interview' (such as playing together or doing an activity) at home or another suitable location. I may be asked to join.
    Yes / No

13. Records (e.g. photographs) may be taken of my child's work. Yes / No

14. In the future, researchers may contact me again to follow up on my child's progress.
    Yes / No

By signing below, I agree/consent to take part in this research project.

…………………………………..	…………………………………..	………………
(Printed name of child)	(signature of child)	(Date)
…………………………………..	…………………………………..	………………
(Printed name of parent/guardian)	(signature of parent/guardian)	(Date)
…………………………………..	…………………………………..	………………
(Printed name of researcher)	(signature of researcher)	(Date)

For peer review only - http://bmjopen.bmj.com/site/about/guidelines.xhtml

08/03/2022 Version 4

Model information sheet

For peer review o

Tools for Schools: Development and early testing of a school-based toolkit to support children in school

Information Sheet for Parents, Guardians or Carers: Version 4 Jan 2022

Tools for Schools

For peer review only - http://bmjopen.bmj.com/site/about/guidelines.xhtml

Contents

Glossary 2
Background and aims of this research study 3
Overview of the Tools for School toolkit 4
Your role in the research 5
What does your child need to know? 6
Potential Unintended Consequences 7
Withdrawing your consent 8
Confidentiality and data protection 8
Funding 9
Ethical approval 9
Contact details 10
Complaints 10

FUNDED BY

http://blogs.exeter.ac.uk/toolsforschools/

Glossary

ADHD = Attention Deficit/Hyperactivity Disorder

SEN(D)Co = Special Educational Needs (and Disabilities) Coordinator

TA = Teaching Assistant (also known as Learning Support Assistant, LSA)

For peer review only - http://bmjopen.bmj.com/site/about/guidelines.xhtml

Thank you for showing an interest in this research. It focuses on early testing of a school-based toolkit of behavioural strategies and adjustments. These will improve the school experience of students who are hyperactive, impulsive or have problems with attention, causing difficulties for the child at school.

You have received this information sheet after your child's school have approached you because they think your child might benefit from taking part in this research. Please take time to carefully read this leaflet so that you have the information you need or listen to the audio/video version here. You can also request a meeting with the research team or your school's SENCo to discuss what will happen in the study and if you would like further clarification on the overview of the toolkit and study protocols.

Do ask the research team any questions you have before signing the consent form for you or your child. It is important that you are comfortable for both you and your child to take part in this study. Any other parents, guardians or main carers of the child who might like to take part would also be welcome.

If you decide to participate, we thank you. If you choose not to, there is no disadvantage to you, and we thank you for considering our project.

Background and aims of this research study

Every classroom has at least one child (on average) who will struggle to sit still, pay attention and resist impulses to do things like jumping out of their seat. Strategies are needed to help teachers support these students, but current advice is often complicated, and no child is the same. This can lead to teachers struggling to develop the right strategies for each child.

In this project, we have worked closely alongside experts, teachers and parents to develop a prototype (early version) of a toolkit which includes a range of strategies for teachers to use to support school children. Your child's school has agreed to take part in this research already and think your child would suit this toolkit and research study, which is why we are contacting you. The toolkit will be used with your child for one school term, but we will be collecting information from you, your child and their teachers for one term before this and for one school term after the toolkit is tried.

For peer review only

The aim of this current step of the study is to see if the toolkit is acceptable and practical to school staff, children and their parents. It is important to know that we are not trying to diagnose or "treat" your child. The toolkit is at an early stage, and we are not yet sure how much it will help. But we hope that in the future this toolkit will help children to have a better primary school experience.

Overview of the Tools for School toolkit

The Tools for Schools toolkit will be mainly digital (a website), with several key steps as shown below. We want the toolkit to support your child to remain in the classroom and allow them to access the same education and opportunities as their classmates. Your child's SENCo, class teacher and teaching assistants will use the toolkit with your child in school. To keep you as parents 'in the loop' and to make the most of your knowledge of your child, you will be asked to join in for several parts of the toolkit. You can find out more information about the parts of the toolkit through the videos and descriptions on our website.

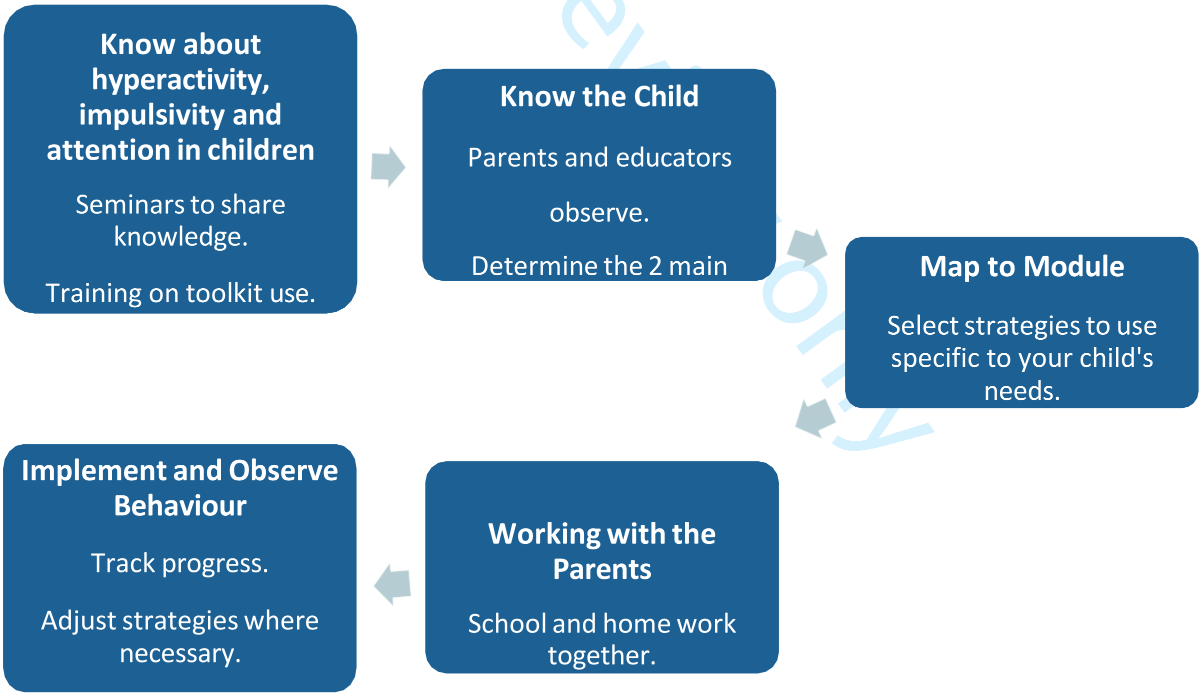

If you would like to find out more about any of these stages/the toolkit, please visit: http://blogs.exeter.ac.uk/toolsforschools/

For peer review only - http://bmjopen.bmj.com/site/about/guidelines.xhtml

Your role in the research

As a parent, guardian or carer of a child using the Tools for Schools intervention, your role will include:

- Possibly completing a brief questionnaire to see if your child is eligible to take part.
- Watching an introductory video and completing training provided by the Tools for Schools research team.
- Completing questionnaires about the healthcare, education and social services you use to support your child, and your child's wellbeing and quality of life (both before and after the toolkit is used).
- Two or three interview conversations with research staff. It is up to you where these take place (e.g. over the phone/zoom, at home or another convenient location for you). These may be recorded
- Taking part in two meetings with your child's teacher and SENDCo to help identify what the toolkit should target to best support your child (you could join these online or in person).
- Working with your child and school staff to use strategies selected in the toolkit.
- Chatting to the research team over the phone about any good and bad things you are experiencing using the toolkit so we can make changes if need be. These may be recorded.
- Using a digital home-school notebook – this will allow you and your child's teacher to share information on your child's progress and ensure strategies are reinforced at home.
- Give the research team permission to collect information about your child from you, school staff and your child.

To show our gratitude, we will provide £20 to your family (£10 for you and £10 for your child) for participating in the interviews.

For peer review only

For peer review only - http://bmjopen.bmj.com/site/about/guidelines.xhtml

We would also like you to give the following permissions on behalf of your child:

- Permission for your child to participate in the research and try the strategies in the toolkit with school staff support.
- Permission for one of the research team to observe your child in school to see how their behaviour changes with different strategies (this will be done subtly without making your child stand out from their classmates and will not disrupt your child's school day).
- Permission for us to ask your child to complete two short age-appropriate questionnaires; one about how they feel about their school, and one about their quality of life (twice during the research study).
- Permission to keep records/photographs of your child's work
- Permission for the research team to conduct an 'active interview' (such as playing together or doing an activity) at home or another suitable location with your child, finding out more about whether they liked or disliked the toolkit strategies. You, another parent/guardian or a sibling can join the child for this if they want you to.

What does your child need to know?

Your child should understand that they are participating in research but it is up to you how much you want to tell them. The toolkit is being tried as part of the usual school day, with SENCos and teachers working to support children in school as usual, so your child need not know that they are doing something new or different if you think this will make them uncomfortable.

We will need your consent for your child to participate, and their verbal agreement that they are happy to take part in a research study and to complete questionnaires. We want to ensure we are mindful of your child's "emotional thermometer" and will not be making your child do something they do not want to do. To achieve this, we will discuss with you the best way to get permission from your child to take part in certain strategies in a way that you and your child are happy with.

Tools for Schools

For peer review only - http://bmjopen.bmj.com/site/about/guidelines.xhtml

Potential Benefits

Benefits for your child include improving their relationship with their teacher and making school a more positive experience for them through:

- School staff and parents becoming better informed about how to work with children who struggle with attention, impulsiveness, or hyperactivity and what it is like for families and children living with children who have issues with this (sometimes diagnosed as ADHD).
- Improvement in teachers' awareness about attention, impulsiveness, or hyperactivity in the classroom.
- Giving teachers easy-to-use strategies to better identify and support students with their attention, impulsiveness, or hyperactivity

Benefits for you include:

- Picking up some tips from the strategies that may help you and your family at home
- Improve your communication with your child's school through the home-school notebook

We hope that access to the Tools for Schools toolkit will help your child's school experience and relationships with school staff. However, the main aim of this study is to test a prototype before it is tried with a wider range of schools and children. In the long run, we hope this will improve support (mostly at school, but also at home) for many children in education.

Potential Unintended Consequences

It is possible that the toolkit may lead to children feeling singled-out from their peers due to being treated differently. We aim to avoid this and any other negative impacts wherever possible so please talk to us about any concerns you may have. Via the toolkit children in the study will hopefully develop social skills and greater self-esteem which may help them build stronger relationships with other children. However, you and your child are free to withdraw at any time if you or they wish to.

Possible negative impacts of the toolkit will be monitored throughout the study and school staff will follow a defined procedure for reporting any negative impacts as soon as possible after they come to light. The research team will abide by any existing safeguarding protocols your child's school has as well.

For peer review only - http://bmjopen.bmj.com/site/about/guidelines.xhtml

Should a child or other participant of the study report severe worsening of symptoms or distress, you will be informed and the use of the toolkit with your child will be temporarily stopped. From there the lead researcher (Dr Abby Russell) will consult with members on the study advisory board to decide on the most appropriate course of action. This can include: a clinician speaking directly to you and the school to see whether the negative effects are caused by the toolkit or research study or are external to this; your child and family withdrawing from the study; or individual plans being put in place to monitor and manage the situation.

Withdrawing your consent

- You (and anyone else involved) may withdraw from participation in the study without needing to provide a reason at any point.
- You can choose to withdraw you or your child's participation and/or any data you and they have provided.
- Data can only be removed so long as the withdrawal is done before the data is anonymised, as at this point, we will not know which information is linked to your individual child.
- Should you withdraw from the study due to negative effects, your existing data will not be used unless you give us consent at the time that you withdraw.
- Your child will be able to continue to access the prototype intervention (as long as the study team are confident that this is not the cause of adverse effects) if they wish.

Confidentiality and data protection

The University of Exeter processes personal data for the purposes of carrying out research in the public interest. The University will endeavour to be transparent about its processing of your personal data and this information sheet should provide a clear explanation of this. If you do have any queries about the University's processing of your personal data that cannot be resolved by the research team, further information may be obtained from the University's Data Protection Officer by emailing informationgovernance@exeter.ac.uk. or at http://www.exeter.ac.uk/ig/

For peer review only - http://bmjopen.bmj.com/site/about/guidelines.xhtml

The data we will collect from you includes recordings of your interviews with the research team, notes taken by the interviewer at the time and any questionnaires that we ask you to complete. The data we will collect from your child includes a recording of their active interview, any questionnaires they complete and information from observing them at school.

Recordings will be written down word for word and anonymised before being analysed along with other interviews we are conducting. The data collected will be securely stored in such a way that only the researchers working on the project can access it. In the interview if you feel uncomfortable, you will be reminded of your right to not answer any particular question(s).

The results of this project may be published, but any data included will not be individually identifiable – so nobody will know which schools or children took part. A summary of the study findings will be sent to you. You will be provided with a copy of your interviews as well if you wish. Additionally, the anonymised data you provide may be used other related research – however this is optional.

Funding

This research is funded by the National Institute of Health Research (NIHR): the research arm of the NHS. Specifically, it is funded by an Advanced Fellowship – Stage 2 <details>. The funding currently covers the project for 5 years, which began in July 2020.

Ethical approval

This study has received ethical approval from the University of Exeter College of Medicine and Health Research Ethics Committee (reference Jan22/B/300).

For peer review only - http://bmjopen.bmj.com/site/about/guidelines.xhtml

Contact details

If you have any questions about the Tools for Schools project, either now or in the future, please feel free visit our website for further information, or to contact [removed for review] directly, who leads the research team:

[Researcher name}

Children and Young people's Mental health
research collaboration (ChYMe)
University of Exeter College of Medicine and Health

[phone numbers]

ToolsForSchools@exeter.ac.uk

[researcher email and twitter

handle]

Complaints

If you have any complaints about the way in which this study has been carried out, please contact the Chair of the University of Exeter Medical School Research Ethics Committee:

cmhethics@exeter.ac.uk

For peer review only - http://bmjopen.bmj.com/site/about/guidelines.xhtml

SPIRIT

STANDARD PROTOCOL ITEMS: RECOMMENDATIONS FOR INTERVENTIONAL TRIALS

SPIRIT 2013 Checklist: Recommended items to address in a clinical trial protocol and related documents*

Section/item	Item No	Description
**Administrative information**		
Title	1	Descriptive title identifying the study design, population, interventions, and, if applicable, trial acronym: included
Trial registration	2a	Trial identifier and registry name. If not yet registered, name of intended registry : Not applicable
	2b	All items from the World Health Organization Trial Registration Data Set: Not applicable
Protocol version	3	Date and version identifier (added in Author contributions)
Funding	4	Sources and types of financial, material, and other support: included
Roles and responsibilities	5a	Names, affiliations, and roles of protocol contributors: included
	5b	Name and contact information for the trial sponsor: funder (included)
	5c	Role of study sponsor and funders, if any, in study design; collection, management, analysis, and interpretation of data; writing of the report; and the decision to submit the report for publication, including whether they will have ultimate authority over any of these activities: in funding section
	5d	Composition, roles, and responsibilities of the coordinating centre, steering committee, endpoint adjudication committee, data management team, and other individuals or groups overseeing the trial, if applicable (see Item 21a for data monitoring committee): Not applicable for feasibility study.
**Introduction**		
Background and rationale	6a	Description of research question and justification for undertaking the trial, including summary of relevant studies (published and unpublished) examining benefits and harms for each intervention: included
	6b	Explanation for choice of comparators: added (introduction)
Objectives	7	Specific objectives or hypotheses: included

For peer review only

Trial design	8	Description of trial design including type of trial (eg, parallel group, crossover, factorial, single group), allocation ratio, and framework (eg, superiority, equivalence, noninferiority, exploratory): added in intro

Methods: Participants, interventions, and outcomes

Study setting	9	Description of study settings (eg, community clinic, academic hospital) and list of countries where data will be collected. Reference to where list of study sites can be obtained: added to methods (description of settings). Site list is NA due to research confidentiality
Eligibility criteria	10	Inclusion and exclusion criteria for participants. If applicable, eligibility criteria for study centres and individuals who will perform the interventions (eg, surgeons, psychotherapists): included
Interventions	11a	Interventions for each group with sufficient detail to allow replication, including how and when they will be administered: included
	11b	Criteria for discontinuing or modifying allocated interventions for a given trial participant (eg, drug dose change in response to harms, participant request, or improving/worsening disease): added to Intervention delivery
	11c	Strategies to improve adherence to intervention protocols, and any procedures for monitoring adherence (eg, drug tablet return, laboratory tests): process evaluation
	11d	Relevant concomitant care and interventions that are permitted or prohibited during the trial: added to procedure
Outcomes	12	Primary, secondary, and other outcomes, including the specific measurement variable (eg, systolic blood pressure), analysis metric (eg, change from baseline, final value, time to event), method of aggregation (eg, median, proportion), and time point for each outcome. Explanation of the clinical relevance of chosen efficacy and harm outcomes is strongly recommended : included (analysis, quantitative measures)
Participant timeline	13	Time schedule of enrolment, interventions (including any run-ins and washouts), assessments, and visits for participants. A schematic diagram is highly recommended (see Figure): included
Sample size	14	Estimated number of participants needed to achieve study objectives and how it was determined, including clinical and statistical assumptions supporting any sample size calculations: included
Recruitment	15	Strategies for achieving adequate participant enrolment to reach target sample size: NA

Methods: Assignment of interventions (for controlled trials) NA

Allocation:		
Sequence generation	16a	Method of generating the allocation sequence (eg, computer-generated random numbers), and list of any factors for stratification. To reduce predictability of a random sequence, details of any planned restriction (eg, blocking) should be provided in a separate document that is unavailable to those who enrol participants or assign interventions
Allocation concealment mechanism	16b	Mechanism of implementing the allocation sequence (eg, central telephone; sequentially numbered, opaque, sealed envelopes), describing any steps to conceal the sequence until interventions are assigned
Implementation	16c	Who will generate the allocation sequence, who will enrol participants, and who will assign participants to interventions
Blinding (masking)	17a	Who will be blinded after assignment to interventions (eg, trial participants, care providers, outcome assessors, data analysts), and how
	17b	If blinded, circumstances under which unblinding is permissible, and procedure for revealing a participant's allocated intervention during the trial
**Methods: Data collection, management, and analysis**		
Data collection methods	18a	Plans for assessment and collection of outcome, baseline, and other trial data, including any related processes to promote data quality (eg, duplicate measurements, training of assessors) and a description of study instruments (eg, questionnaires, laboratory tests) along with their reliability and validity, if known. Reference to where data collection forms can be found, if not in the protocol: included
	18b	Plans to promote participant retention and complete follow-up, including list of any outcome data to be collected for participants who discontinue or deviate from intervention protocols: NA, measuring this as part of study aims
Data management	19	Plans for data entry, coding, security, and storage, including any related processes to promote data quality (eg, double data entry; range checks for data values). Reference to where details of data management procedures can be found, if not in the protocol: added to measures section
Statistical methods	20a	Statistical methods for analysing primary and secondary outcomes. Reference to where other details of the statistical analysis plan can be found, if not in the protocol: included
	20b	Methods for any additional analyses (eg, subgroup and adjusted analyses): included

For peer review only - http://bmjopen.bmj.com/site/about/guidelines.xhtml

	20c	Definition of analysis population relating to protocol non-adherence (eg, as randomised analysis), and any statistical methods to handle missing data (eg, multiple imputation): Not applicable
**Methods: Monitoring**		
Data monitoring	21a	Composition of data monitoring committee (DMC); summary of its role and reporting structure; statement of whether it is independent from the sponsor and competing interests; and reference to where further details about its charter can be found, if not in the protocol. Alternatively, an explanation of why a DMC is not needed: Not applicable
	21b	Description of any interim analyses and stopping guidelines, including who will have access to these interim results and make the final decision to terminate the trial: included
Harms	22	Plans for collecting, assessing, reporting, and managing solicited and spontaneously reported adverse events and other unintended effects of trial interventions or trial conduct: included
Auditing	23	Frequency and procedures for auditing trial conduct, if any, and whether the process will be independent from investigators and the sponsor: not applicable
**Ethics and dissemination**		
Research ethics approval	24	Plans for seeking research ethics committee/institutional review board (REC/IRB) approval: included
Protocol amendments	25	Plans for communicating important protocol modifications (eg, changes to eligibility criteria, outcomes, analyses) to relevant parties (eg, investigators, REC/IRBs, trial participants, trial registries, journals, regulators): included in declarations
Consent or assent	26a	Who will obtain informed consent or assent from potential trial participants or authorised surrogates, and how (see Item 32): included
	26b	Additional consent provisions for collection and use of participant data and biological specimens in ancillary studies, if applicable: included
Confidentiality	27	How personal information about potential and enrolled participants will be collected, shared, and maintained in order to protect confidentiality before, during, and after the trial: included in ethics and dissemination
Declaration of interests	28	Financial and other competing interests for principal investigators for the overall trial and each study site: included
Access to data	29	Statement of who will have access to the final trial dataset, and disclosure of contractual agreements that limit such access for investigators: included

review only

Ancillary and post-trial care	30	Provisions, if any, for ancillary and post-trial care, and for compensation to those who suffer harm from trial participation: NA
Dissemination policy	31a	Plans for investigators and sponsor to communicate trial results to participants, healthcare professionals, the public, and other relevant groups (eg, via publication, reporting in results databases, or other data sharing arrangements), including any publication restrictions: included
	31b	Authorship eligibility guidelines and any intended use of professional writers: added
	31c	Plans, if any, for granting public access to the full protocol, participant-level dataset, and statistical code: included
**Appendices**		
Informed consent materials	32	Model consent form and other related documentation given to participants and authorised surrogates: included in supplementary material
Biological specimens	33	Plans for collection, laboratory evaluation, and storage of biological specimens for genetic or molecular analysis in the current trial and for future use in ancillary studies, if applicable: not applicable

*It is strongly recommended that this checklist be read in conjunction with the SPIRIT 2013 Explanation & Elaboration for important clarification on the items. Amendments to the protocol should be tracked and dated. The SPIRIT checklist is copyrighted by the SPIRIT Group under the Creative Commons "Attribution-NonCommercial-NoDerivs 3.0 Unported" license.

For peer review only
